# Incidence and outcome of SARS-CoV-2 reinfection in the pre-Omicron era: A global systematic review and meta-analysis

**DOI:** 10.7189/jogh.13.06051

**Published:** 2023-11-24

**Authors:** Nabihah Farhana Ismail, Ahmed Ehsanur Rahman, Durga Kulkarni, Fuyu Zhu, Xin Wang, Graciela del Carmen Morales, Amit Srivastava, Kristen E Allen, Julia Spinardi, Moe H Kyaw, Harish Nair

**Affiliations:** 1 Centre for Global Health, University of Edinburgh, Edinburgh, United Kingdom; 2 Communicable Disease Control Unit, Public Health Department, Johor State, Malaysia; 3 International Centre for Diarrhoeal Diseases Research, Bangladesh; 4 School of Public Health, Nanjing Medical University, Jiangsu, China; 5 Pfizer, Vaccines, Emerging Markets; 6 Orbital Therapeutics, United States of America; 7 MRC/Wits Rural Public Health and Health Transitions Research Unit (Agincourt), School of Public Health, Faculty of Health Sciences, University of the Witwatersrand, Johannesburg, South Africa

## Abstract

**Background:**

With the emergence of new variants and sub-lineages of severe acute respiratory syndrome coronavirus 2 (SARS-CoV-2), reinfections can significantly impact herd immunity, vaccination policies, and decisions on other public health measures. We conducted a systematic review and meta-analysis to synthesise the global evidence on SARS-CoV-2 reinfections in the pre-Omicron era.

**Methods:**

We searched five global databases (MEDLINE, Embase, CINAHL Plus, Global Health, WHO COVID-19) on 12 May 2022 and 28 July 2023 and three Chinese databases (CNKI, Wanfang, CQvip) on 16 October 2022 for articles reporting incidence and outcomes of SARS-CoV-2 reinfection before the period of Omicron (B.1.1.529) predominance. We assessed risk of bias using Joanna Briggs Institute critical appraisal tools and conducted meta-analyses with random effects models to estimate the proportion of SARS-CoV-2 reinfection among initially infected cases and hospitalisation and mortality proportions among reinfected ones.

**Results:**

We identified 7593 studies and extracted data from 64 included ones representing 21 countries. The proportion of SARS-CoV-2 reinfection was 1.16% (95% confidence interval (CI) = 1.01-1.33) based on 11 639 247 initially infected cases, with ≥45 days between the two infections. Healthcare providers (2.28%; 95% CI = 1.37-3.40) had a significantly higher risk of reinfection than the general population (1.00%; 95% CI = 0.81-1.20), while young adults aged 18 to 35 years (1.01%; 95% CI = 0.8-1.25) had a higher reinfection burden than other age groups (children <18 years old: 0.57%; 95% CI = 0.39-0.79, older adults aged 36-65 years old: 0.53%; 95% CI = 0.41-0.65, elderly >65 years old: 0.37%; 95% CI = 0.15-0.66). Among the reinfected cases, 8.12% (95% CI = 5.30-11.39) were hospitalised, 1.31% (95% CI = 0.29-2.83) were admitted to the intensive care unit, and 0.71% (95% CI = 0.02-2.01) died.

**Conclusions:**

Our data suggest a relatively low risk of SARS-CoV-2 reinfection in the pre-Omicron era, but the risk of hospitalisation was relatively high among the reinfected cases. Considering the possibility of underdiagnosis, the reinfection burden may be underestimated.

**Registration:**

PROSPERO: CRD42023449712.

The first case of severe acute respiratory syndrome coronavirus 2 (SARS-CoV-2) reinfection, which can be caused by the same or different variant from the initial infection, was diagnosed on 25 August 2020 in Hong Kong [1-3]. Such cases were assumed to be rare early on in the pandemic, as most studies reported reinfection confirmed by whole genomic sequence alone [2-4]. Several later studies reported varying rates and burdens of reinfection [5-7], potentially reflecting the inability of the primed immune system to sustain protection against subsequent infections or the higher capabilities of the new variants to evade natural immunity [8,9], as well as a wide range of risk factors and adverse outcomes [5,10,11].

As SARS-CoV-2 constantly mutates, it is important to systematically synthesise evidence related to reinfection to support effective public health measures. The Omicron (B.1.1.529) variant is phylogenetically and antigenically distinct from earlier ones, with a substantially higher risk of immune escape [12,13]. We aimed to estimate the global burden of SARS-CoV-2 reinfection before the period of Omicron predominance, stratified by age, sex, region, and country income group. We also synthesised the evidence related to its clinical severity and outcomes, including hospitalisation, intensive care unit (ICU) admission, and mortality.

## METHODS

We registered the protocol of this systematic review in the International prospective register of systematic reviews (PROSPERO) (CRD42023449712) and conducted it per the Preferred Reporting Items for Systematic Review and Meta-Analysis Protocols (PRISMA-P 2020) guidelines [14]. Since we used data from published primary studies, we did not seek formal ethical approval.

We searched five global databases (MEDLINE, Embase, Global Health, CINAHL, WHO COVID-19) on 12 May 2022 and again on 28 July 2023, and three Chinese databases (CNKI, Wanfang, CQvip) on 16 October 2022, with a broad search strategy containing keywords such as “Coronavirus disease (COVID-19)” and “Reinfection” (Table S1 in the **Online Supplementary Document**).

Our study population were individuals with real-time reverse transcription polymerase chain reaction (RT-PCR)-confirmed SARS-CoV-2 infection. The primary outcome of interest was reinfection, defined as an individual with an initial infection determined by a positive RT-PCR test for SARS-CoV-2 and a second positive RT-PCR test at least 45 days from the initial infection. Per pre-defined eligibility criteria (Table S2 in the **Online Supplementary Document**), we included observational (prospective and retrospective) studies, all controlled arms of cluster or individual randomised controlled trials, quasi-experimental studies, and population and facility-based studies conducted in all countries, irrespective of income-group status. We set no language restriction, but excluded studies published before January 2020. We excluded case studies, case series, conference abstracts, technical reports, pre-print publications, systematic reviews and studies that involved fewer than 100 patients with initial infection.

Two reviewers (NFI and AER for English language databases, FZ and XW for Chinese databases) independently screened the titles and abstracts of retrieved studies, followed by the full texts of the ones selected as possibly eligible. They then independently extracted data using a pre-designed form following an initial pilot. We assessed risk of bias using the Joanna Briggs Institute (JBI) Critical Appraisal Tools [15]. A single reviewer (NFI or DK) conducted title and abstract screening and full-text review for the English database update on 28 July 2023. A single reviewer (NFI) extracted data which were then cross-checked by a second reviewer (DK). NFI performed risk of bias assessment for the additional studies. We extracted information on the predominant SARS-CoV-2 variants in countries where the studies were conducted from the CoVariants website if these were not reported in the article [16]. When data was unavailable for a specific country, we used data from the nearest neighboring country as a proxy. The discrepancies between the two pairs of reviewers (NFI and AER, and NFI and DK) were first discussed and resolved with a third reviewer (HN) if consensus was not reached.

### Statistical analysis

We conducted all statistical analyses in Stata, version 15 (StataCorp LLC, College Station, Texas, USA). We adopted a random effects model to report the pooled proportion of SARS CoV-2 reinfection among initially infected cases (reinfection incidence proportion) [17] and further disaggregated the pooled estimates by age, sex, vaccination status, the predominant variant of circulation, study region (Asia, Europe, North and South America), the World Bank country-income group, study design (prospective and retrospective), cut-off duration (≥45 days, ≥60 days, and ≥90 days) and sample sizes (<400, 400-900, and ≥1000) based on data availability. We also reported the rate of reinfection (number of episodes) per 10 000 days after the initial infection (reinfection incidence rate) and estimated the pooled proportions of hospitalisation, ICU admission, and mortality among reinfected cases through a random effects model [17].

We conducted sensitivity analysis by estimating the hospital admission and mortality proportions by sample size (<100 or ≥100). We also did a subgroup meta-analysis for prospective design studies by population type and leave-one-out analysis to demonstrate influential studies.

We reported 95% confidence intervals (CIs) for all pooled estimates, as well as *I*^2^ and *Tao*^2^ values to demonstrate the level of heterogeneity across studies. We also conducted the meta-analyses using R, version 4.2.0. (R Core Team, Vienna, Austria) to check for the reliability of the meta-estimates.

## RESULTS

### Study selection

**Figure 1:**
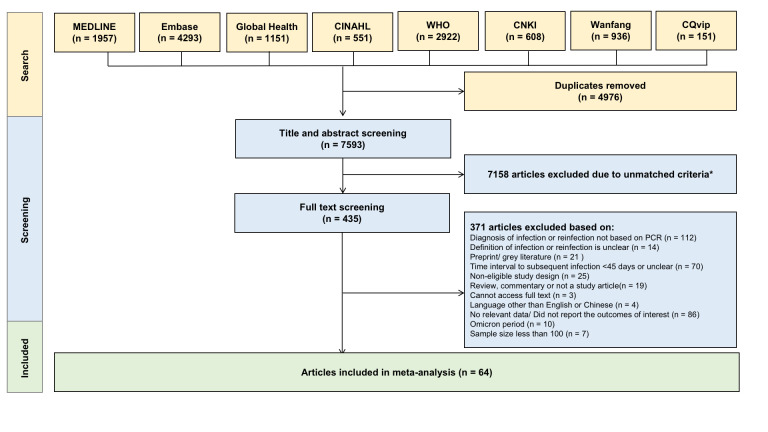
Search outputs based on PRISMA guidelines [14]. *Unmatched criteria based on the inclusion and exclusion criteria.

We identified 12 569 records in the initial literature search, of which 4976 were duplicates. We conducted title and abstract screening of 7593 records and selected 435 studies for full-text review based on pre-determined inclusion and exclusion criteria. After a full-text review, we extracted data from 64 articles (**Figure 1**). Of the excluded articles, 112 did not use an RT-PCR test to confirm the initial infection or reinfection, 86 did not provide relevant data, 70 did not have a minimum duration of 45 days between the initial infection and reinfection, 25 had the non-eligible study design, 21 were preprints or gray literature, 19 were non-original articles, 14 had an unclear definition for reinfection, ten were conducted after Omicron predominance, seven had a sample size of less than 100, four were in non-English or Chinese language, and three had no accessible full-texts (Table S3 in the **Online Supplementary Document**).

### Study characteristics

Among the 64 included studies, 19 were prospective and 45 were retrospective (Table S4 in the **Online Supplementary Document**). Most were conducted in Europe (27 studies: six from the UK [6,18-22]), six from Italy [23-28], five from Denmark [5,29-32], four from Spain [33-36], two from France [37,38], and one each from Austria [39], Poland [40], Czech [41] and Sweden [42]), followed by Asia (23 studies: seven from Turkey [43-49], four each from India [50-53] and Israel [54-57], two each from Qatar [58,59], Iran [60,61], and Saudi Arabia [62,63], and one each from Bangladesh [64] and Kuwait [65]), North and South America (13 studies: 10 from the USA [66-75] and three from Brazil [76-78]) and Africa (one study from Liberia [79]). Forty-six studies were from high-income countries (HICs), 10 from upper-middle-income countries (UMICs), seven from lower-middle income countries (LMICs) and was one from a lower-income country (LIC). Forty-seven studies were conducted among the general population, 12 among health care providers, one with a mixed population (general population and health care providers), and four were conducted among special-risk populations, such as patients with autoimmune diseases and long-term care facility residents. Twenty-six studies included the period of Delta predominance, and 15 were conducted when no Variant of Concerns (VOCs) predominated (Figure S1 in the **Online Supplementary Document**).

### Results of syntheses

We extracted incidence proportion estimates from all 64 studies and incidence rate estimates from 10, while only 15 reported on severity. Regarding SARS-CoV-2 reinfection outcomes, one study reported on oxygenation, 23 on hospitalisation, 13 on ICU admission, four on mechanical ventilation, and 23 on mortality.

The total sample comprised 11 639 247 patients with initial SARS-CoV-2 infection. Among them, 7 748 062 were from the general population, 23 931 were health service providers, 3 860 054 were mixed, and 7200 were special-risk populations. However, some studies did not have age and sex-specific reporting of incidence (Table S5 in the **Online Supplementary Document**).

#### Incidence proportion

**Figure 2:**
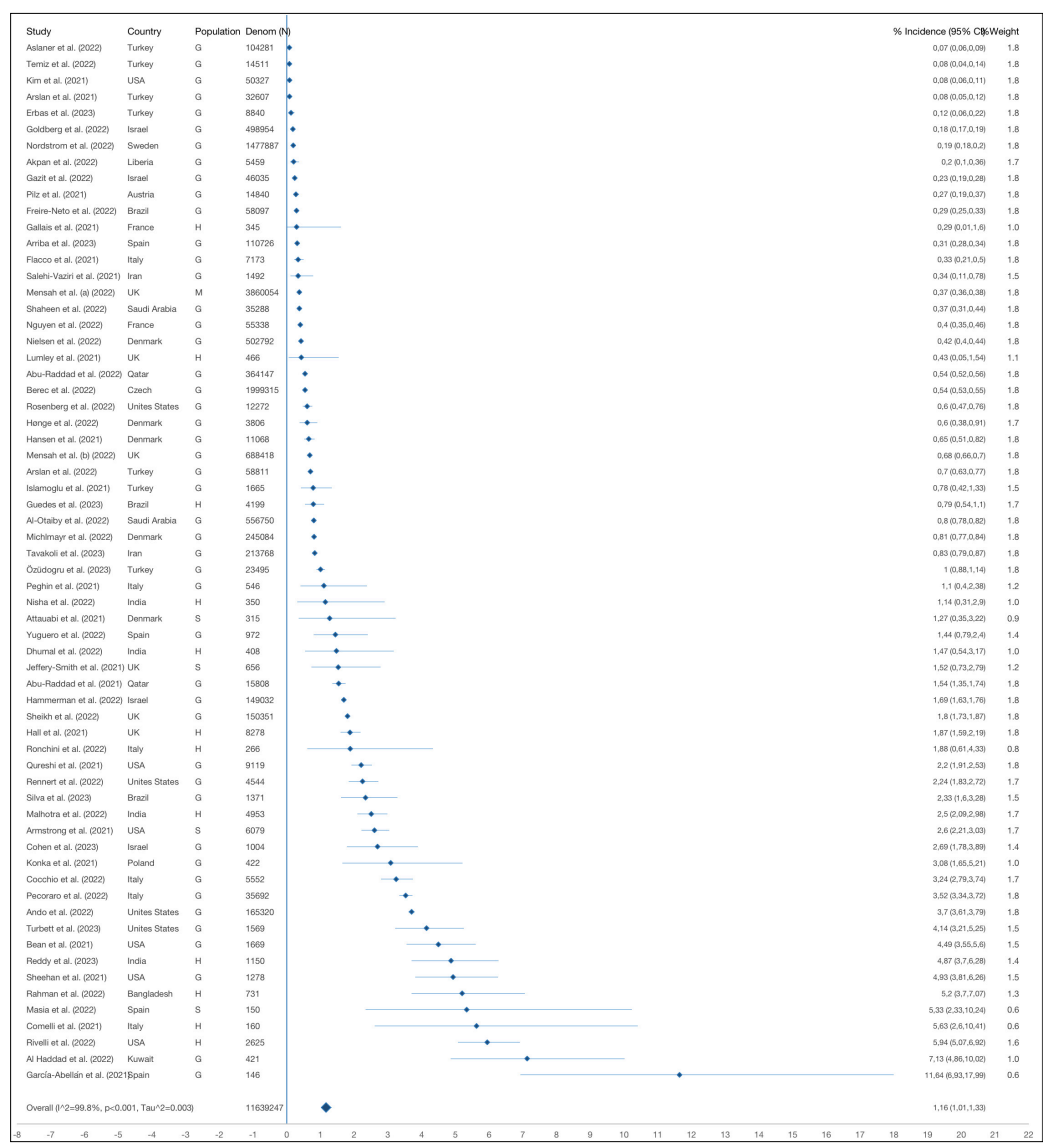
Pooled incidence proportion (with 95% CI) of SARS CoV-2 reinfection generated from random effects model. G – general population, H – health service provider, M – mixed populations, S – special risk group (long term care facilities residents and persons with autoimmune diseases).

The pooled reinfection incidence proportion was 1.16% (95% CI = 1.01-1.33) (**Figure 2**), being lowest in a study conducted among the general population in Turkey [48] (0.07%; 95% CI = 0.06-0.09) with 104 281 initially infected cases (minimum cut-off 45 days) and highest in another conducted among the general population in Spain [33] (11.64%; 95% CI = 6.93-17.99) with 146 initially infected cases (minimum cut-off 60 days).

**Figure 3:**
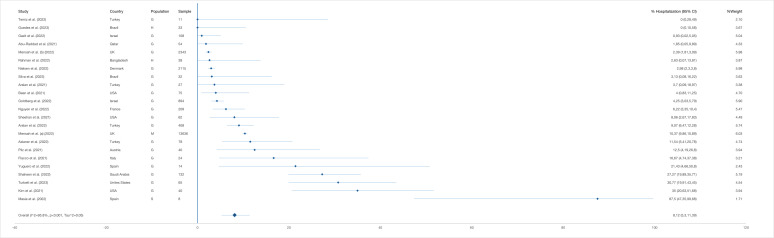
Pooled incidence proportion (with 95% CI) of SARS CoV-2 reinfection generated from random effects model by background characteristics. G – general population, H – health service provider, M – mixed populations, S – special risk group (long term care facilities residents and persons with autoimmune diseases), HIC – high-income country.

The reinfection risk was significantly higher among health care providers (2.28%; 95% CI = 1.37-3.40) than the general population (1.00%; 95% CI = 0.81-1.20) (**Figure 3**), and was also significantly higher among young adults aged 18 to 35 years (1.01%; 95% CI = 0.8-1.25) than other age groups (children <18 years old: 0.57%; 95% CI = 0.39-0.79, older adults aged 36-65 years old: 0.53%; 95% CI = 0.41-0.65, elderly >65 years old: 0.37%; 95% CI = 0.15-0.66). When we restricted the analysis to studies with only unvaccinated populations (n = 3 460 439), the reinfection incidence proportion was 0.83% (95% CI = 0.61, 1.08), with no significant difference in studies with populations with mixed vaccination status (1.36%; 95% CI = 1.09-1.66). We observed no significant difference across other background characteristics and study settings (Table S6, panels A-I in the **Online Supplementary Document**).

#### Incidence rate

The pooled reinfection incidence rate was 1.64 (95% CI = 1.41-1.87) episodes per 10 000 person-days after the initial infection (Table S7 in the **Online Supplementary Document**) and was lowest in a study conducted in Kuwait (0.10 episodes per 10 000 person-days; 95% CI = 0.07-0.15) and highest in one conducted in Israel (10.21 per 10 000 person-days; 95% CI = 9.82-10.62).

#### Hospitalisation

**Figure 4:**
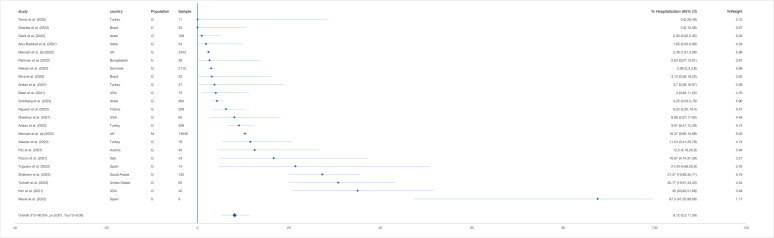
Pooled proportion of hospitalisation (with 95% CI) among SARS-CoV-2 reinfected cases generated from random effects model. G – general population, H – health service provider, M – mixed populations, S – special risk group (long term care facilities residents and persons with autoimmune diseases).

The pooled proportion of hospitalisation was 8.12% (95% CI = 5.30-11.39) among 20 446 reinfection cases (**Figure 4**). Stratified by country, it was 5.90% (95% CI = 2.10-10.99) in studies conducted in Asia (n = 1750) and 9.30% (95% CI = 4.33-15.73) in those conducted in Europe (n = 18 389). By income group, 10.00% (95% CI = 6.34-14.30) reinfected patients (n = 19 819) were hospitalised in HICs and around 4.80% (95% CI = 1.89-8.60) (n = 627) in non-HICs. Among the reinfected cases (n = 21 849), 1.31% (95% CI = 0.29-2.83) were admitted to an ICU (1.98%; 95% CI = 0.65-3.82), without the studies reporting zero events (Table S8, panels A-B in the **Online Supplementary Document**). Only one study from Bangladesh [64] reported supplemental oxygen need to be 2.63% (n = 1) among reinfected patients.

#### Mortality

**Figure 5:**
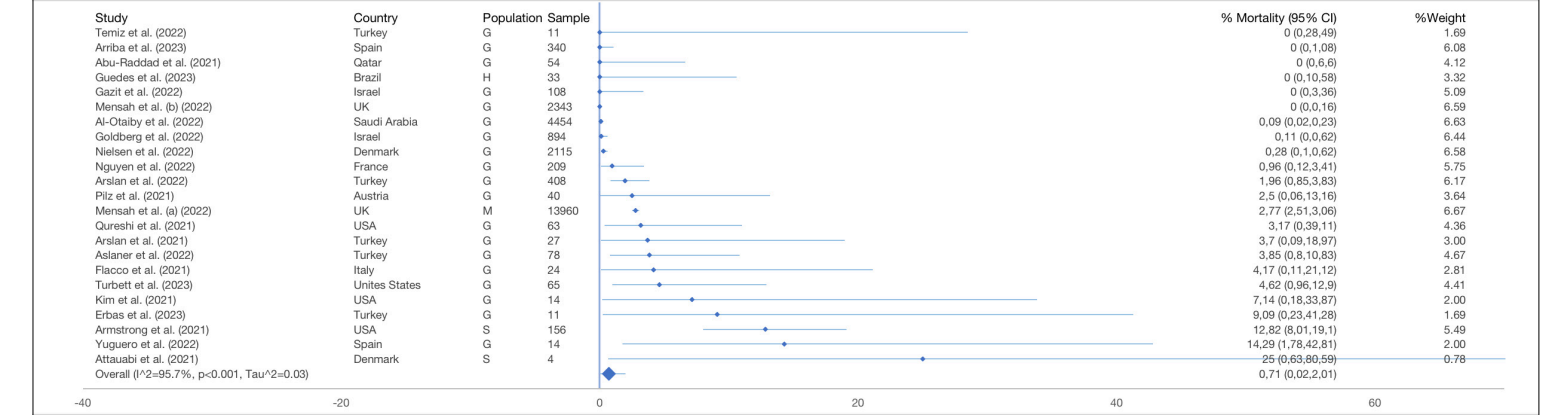
Pooled proportion of mortality (with 95% CI) among SARS-CoV-2 reinfected cases generated from random effects model. G – general population, H – health service provider, M – mixed populations, S – special risk group (long term care facilities residents and persons with autoimmune diseases).

The pooled proportion of mortality was 0.71% (95% CI = 0.02-2.01) based on 25 425 reinfected cases (**Figure 5**). Six studies reported zero mortalities, while a study in Spain reported the highest mortality among the general population (14.29%; 95% CI = 1.78-42.81) with a sample of 14 reinfected cases [34]. The pooled mortality proportion was 1.58% (95% CI = 0.29-3.53) after excluding the studies reporting zero deaths (n = 22 536) (Table S9, panels A-B in the **Online Supplementary Document**).

#### Other outcomes

We could not report pooled estimates for SARS-CoV-2 reinfection severity level and oxygenation and mechanical ventilation requirements due to the small sample size and lack of data and standardisation in reporting. We only determined that most reinfected cases were mild to moderate severity with a low requirement for mechanical ventilation (Table S10 **Online Supplementary Document**).

### Sensitivity analysis

We found higher incidence reported by prospective studies (1.96%; 95% CI = 1.43-2.57) than retrospective studies (0.97%; 95% CI = 0.81-1.14). The pooled reinfection incidence proportion was not sensitive to sample size (400, 400-900, and ≥1000) and minimum cut-off duration (≥60 and ≥90 days) (**Figure 3**). Also, we found no significant difference in other sensitivity analyses and no influential studies from the leave-one-out analysis (Table S11 and Figures S2-S3 in the **Online Supplementary Document**).

### Quality assessment

We found no low-quality studies with a high potential for bias in the outcomes we measured, so we did not do a subgroup analysis based on the quality assessment results (Table S12, panels A-B in the **Online Supplementary Document**).

## DISCUSSION

This systematic review consolidates the global evidence on the incidence and clinical outcomes of SARS-CoV-2 reinfection in the pre-Omicron period. We observed that, although the risk of SARS-CoV-2 reinfection was relatively low (less than 2%), about 9% of reinfected cases required hospitalisation, and about 2% were admitted to ICU or died. We did not observe significant differences in reinfection incidence by age groups, gender, vaccination status, region, or country income groups. However, the incidence was relatively higher among young adults and health care workers and in prospective studies.

We believe this to be the most comprehensive review on SARS-CoV-2 reinfection before the Omicron predominant period to date due to the use of a detailed search strategy within eight bibliographic databases, including three Chinese ones. We reported the pooled incidence of reinfections based on more than 11 million patients with initial SARS CoV-2 infection confirmed by the gold standard RT-PCR test. This estimate is generated based on 64 international peer-reviewed articles from 21 countries, including ones from both Asia and Europe and different country income groups.

The pooled reinfection incidence proportion in our review (1.16%; 95% CI = 1.01-1.33) is higher than a previously conducted review (0.65%; 95% CI = 0.39-0.98]) [7]. The lower estimate reported by Mao et al. [7] could be due to the included studies being conducted before the period of Delta predominance. We believe that our estimates are reliable, as the pooled reinfection proportion was around 1% in studies with large sample sizes (≥1000), and as we excluded studies with small sample sizes (less than 100 initially infected persons). We also used a minimum cut-off duration of 45 days between the initial infection and reinfection episode, which helped develop a stable estimate as the pooled incidence proportions did not change significantly with longer cut-offs, such as >60 days and >90 days, indicating that these estimates are not sensitive to analytic approaches. These data suggest that reinfection is uncommon and does not increase over time following the initial infection in the pre-Omicron period.

The relatively low reinfection incidence proportion may be related to the robust immune response developed after the initial infection [80,81] or the timing of the review. More than half of the included studies were conducted before the Delta predominant period in some countries, which was more infectious than the earlier VOCs [82]. Our findings also support the hypothesis that reinfection is a function of the risk of exposure. We found that the proportion of reinfection was significantly higher among health care providers than among the general population, which is consistent with the findings of other systematic reviews [7,10]. The higher risk in health care providers may also be explained by the study design, where eight of the 12 studies were prospective, a lower threshold for testing when being symptomatic, and more regular testing at predefined intervals even if asymptomatic. However, we could not test these factors in our analyses. However, when we restricted the analysis to only prospective studies, although health care providers appeared to have a higher risk (2.25%; 95% CI = 1.03-3.89) similar to the special risk group (2.16%; 95% CI = 0.69-4.32) than the general population (1.59%; 95% CI = 0.95-2.39), the difference was not significant. We also found that young adults had the highest reinfection risk among all age groups, even when compared to the elderly group aged 65 years and above. This increased risk may be due to the higher level of exposure that they experienced due to their involvement in income-generating activities and more frequent and higher-density social interactions [83].

We found no notable difference in reinfection incidence proportions between unvaccinated populations and populations with mixed vaccination status. This finding could be influenced by the individual studies’ data collection period. Earlier studies were primarily conducted among unvaccinated populations when the predominant SARS-CoV-2 strain was less infectious, while later ones were conducted among populations with mixed vaccination status when the predominant SARS-CoV-2 strain was more infectious [82]. Therefore, the protective effect of vaccination on the reinfection burden could have been underestimated due to the increased risk posed by the more infectious strains. In our review, the highest reinfection incidence rate (10.21 episodes of reinfection per 1000 person-years after the initial infection) was reported by Hammerman et al. [54], who conducted studies among unvaccinated individuals with SARS-CoV-2 initial infection including the period of Alpha and Delta predominance. Chen et al. [10] suggested that the natural immunity in unvaccinated individuals appeared to decrease slowly over time, and several studies suggested potentially short and dysfunctional protection from initial infection, especially against new variants [80,81,84].

In our study, the risk of hospitalisation due to SARS-Cov-2 reinfection was around 9%. As hospitalisation criteria for COVID-19 have become stricter over time [85], we believe that this proportion can be interpreted as a proxy to severity, particularly for reinfected cases which occurred later during the pandemic. Moreover, our data show that 1.3% of reinfected patients were admitted to ICUs. A recent large study in the US reported that people with SARS-CoV-2 reinfection were more likely to be hospitalised during reinfection than the initial infection (hazard ratio (HR) = 3.32; 95% CI = 3.13-3.51) [86]. Reinfection could lead to more extensive cumulative damage to the body and complications in people with underlying medical conditions like diabetes or chronic kidney and heart diseases, therefore resulting in higher hospitalisation among reinfected patients [86]. We were unable to analyse factors associated with hospitalisation due to limited data. However, when vaccination status was considered, Nordstrom et al. [42] found that both one-dose and two-dose vaccinations among reinfected individuals were associated with a lower risk of hospitalisation when compared to reinfected individuals who were unvaccinated.

In our review, the pooled proportion of mortality in patients with SARS-CoV-2 reinfection was 0.71%. However, this could be an overestimation of the true mortality risk, as most studies reported case fatality rates instead of disease-attributable mortality rates. The relatively low death proportion may again be due to the protective role of natural immunity, which leads to less severe disease during subsequent infections [80,81]. Our estimate is comparable to the case fatality rate (1.00%; 95% CI = 1.00-3.00) reported for non-hospitalised people with an initial SARS-CoV-2 infection [87]. In our review, Attauabi et al. [31], who conducted the study among patients with inflammatory bowel diseases, reported the highest proportion of mortality (25.00%), higher than that reported among patients hospitalised with SARS-CoV-2 infection by Mojtaba et al. (13.00%; 95% CI = 9.00-17.00) [87]. Additionally, Bowe et al. [86] found a higher risk (HR = 2.17; 95% CI = 1.93-2.45) of death when comparing people with at least two SARS-CoV-2 infections to the first SARS-CoV-2 infection. Therefore, special attention should be given to people with risk factors suffering from reinfection.

Our systematic review has some limitations. The included studies came from various settings during different phases of the pandemic, with different predominant SARS-CoV-2 strains and population vaccination coverage. We could not address this heterogeneity while synthesising the evidence. Furthermore, we only included studies confirming SARS-CoV-2 infections with an RT-PCR test. As reinfections tend to be milder than the initial infection and thus are less likely to get tested, which can lead to under-ascertainment and misclassification. Several nationwide surveys concluded that reinfection might be underestimated severalfold when the prevalence of antibodies against SARS-CoV-2 in the population was compared with the number of confirmed cases [88]. We could not study the effect of vaccination on reinfection at an individual level. Additionally, inconsistent definitions and criteria used across studies for reinfections, severity, outcomes, and mortality also limit our interpretation. Moreover, most studies reported severity at a one-time point, usually at diagnosis or admission, which did not necessarily reflect the whole course of the disease. Due to the limited genomic data reported in the articles, we could not analyse the relationship between SARS-CoV-2 variants and reinfection risk. Although we took the time interval of ≥45 days between the infections, we cannot exclude protracted infections that are misclassified as reinfection. As people with primary immunodeficiencies or immunocompromised due to underlying medical conditions are more at risk of having either protracted SARS-CoV-2 infections or multiple new infections with possibly worse outcomes [89,90], well-designed prospective cohort studies on these populations who have recovered from COVID-19 as confirmed by negative PCR tests or whole genome sequencing tests, together with serological studies are required to understand this factor better so it can be monitored and compiled into a coherent database.

While the global efforts to ensure vaccine equity should continue, primary and booster vaccination for individuals with prior infections should not be deprioritised, especially for those with high exposure risks. Long-term outbreak prevention measures should continue, where the public needs to be continually informed about the risk of reinfection and to continue with self-preventive measures, testing, and self-isolation. The public and clinicians should be informed of the potential for severe disease and mortality risk during reinfection, particularly in high-risk groups. There is a need to develop the capacity for enhanced genomic surveillance to tailor public health measures and allow for rapid response.

## CONCLUSIONS

Our pooled analysis showed a low risk of SARS-CoV-2 reinfection in the pre-Omicron period. However, reinfection appears to increase the risk of hospitalisation. Since many countries now advocate for self-testing, steps must be taken to ensure adequate sample collection to determine the actual burden of SARS-CoV-2 reinfection and its implications on patients and health care systems. Improved national epidemiological surveillance, including genomic sequencing of SARS-CoV-2 variants with a well-established linkage to clinical databases across different regions and countries, would improve public health planning and provide robust data for research.

## Additional material


Online Supplementary Document
