# Supplementary material for: Incidence and outcome of SARS-CoV-2 reinfection in the pre-Omicron era: A global systematic review and meta-analysis
Source: J Glob Health. 2023 Nov 24;13:06051. doi: 10.7189/jogh.13.06051 (PMC10667793; doi:10.7189/jogh.13.06051)
Supplement: Online Supplementary Document [file jogh-13-06051-s001.pdf]

Supplementary materials

Table S1: Search strategy and search strings

Five English language databases: The searches were run on 12/05/2022 and updated on 28/07/2023.

CINAHL

551

#	**Query**
S1	(MH "COVID-19") OR (MH "SARS-CoV-2")
S2	TI ((COVID OR "COVID-19*" or "Coronavirus disease 2019*" or "Coronavirus 2019*" or "COVID-2019*" or "SARS-CoV-2" or "SARS CoV 2" or "SARS-CoV-2019")) OR AB ((COVID or "COVID-19*" or "Coronavirus disease 2019*" or "Coronavirus 2019*" or "COVID-2019*" or "SARS-CoV-2" or "SARS CoV 2" or "SARS-<br>CoV-2019"))
S3	S1 OR S2
S4	(MH "Reinfection")
S5	(MH "Recurrence")
S6	TI (reinfect* or "re-infect*") OR AB ((reinfect* or "re-infect*")
S7	TI ("repeat infection*" or "repeated infection*") OR AB ("repeat infection*" or "repeated infection*")
S8	(TI recurre* OR AB recurre*) N3 (TI ((Covid or "Covid-19*" or "Sars-Cov-2" or PCR or PCRs or "RT-PCR" or "RT-PCRs" or positiv*)) OR AB ((Covid or "Covid-19*" or "Sars-Cov-2" or PCR or PCRs or "RT-PCR" or "RT-PCRs" or positiv*)))
S9	((TI (reactivat* or re-activat*)) or (AB (reactivat* or re-activat*)) N5 (TI (Covid or "Covid-19*" or "Sars-Cov-2") OR AB (Covid or "Covid-19*" or "Sars-Cov-2"))
S10	TI ("Re-positiv*" or repositiv* or "repeat positiv*" or "repeated positiv*") OR AB ("Re-positiv*" or repositiv* or "repeat positiv*" or<br>"repeated positiv*")
S11	S4 OR S5 OR S6 OR S7 OR S8 OR S9 or S10
S12	S3 and S11
S13	Limiters - Published<br>Date: 20200101-20220512

EMBASE
4293

#	Query
1	Coronavirus Disease 2019/ or exp Severe acute respiratory syndrome coronavirus 2/
2	(Covid or COVID-19* or Coronavirus disease 2019* or Coronavirus 2019* or COVID-2019* or SARS-CoV-2 or SARS CoV 2 or SARS-CoV-2019).ti,ab.
3	1 or 2
4	reinfection/ or recurrent infection/
5	recurrent disease/
6	(reinfect* or re-infect*).ti,ab.
7	(repeat infection* or repeated infection*).ti,ab.
8	(recurre* adj3 (Covid or Covid-19* or Sars-Cov-2 or PCR or PCRs or RT-PCR or RT-PCRs or positiv*)).ti,ab.
9	((reactivat* or re-activat*) adj5 (Covid or Covid-19* or Sars-Cov-2)).ti,ab.
10	(Re-positiv* or repositiv* or repeat positiv* or repeated positiv*).ti,ab.
11	4 or 5 or 6 or 7 or 8 or 9 or 10
12	3 and 11
13	(2020* or 2021* or 2022*).em.
14	12 and 13

Global Health
1151

#	Query
1	Betacoronavirus/ or Human Coronaviruses/ or exp severe acute respiratory syndrome-related coronavirus/
2	(Covid or COVID-19* or Coronavirus disease 2019* or Coronavirus 2019* or COVID-2019* or SARS-CoV-2 or SARS CoV 2 or SARS-CoV-2019).ti,ab.
3	1 or 2
4	reinfection/
5	(reinfect* or re-infect*).ti,ab.
6	(repeat infection* or repeated infection*).ti,ab.
7	(recurre* adj3 (Covid or Covid-19* or Sars-Cov-2 or PCR or PCRs or RT-PCR or RT-PCRs or positiv*)).ti,ab.
8	((reactivat* or re-activat*) adj5 (Covid or Covid-19* or Sars-Cov-2)).ti,ab.
9	(Re-positiv* or repositiv* or repeat positiv* or repeated positiv*).ti,ab.
10	4 or 5 or 6 or 7 or 8 or 9
11	3 and 10
12	("2020" or "2021" or "2022").dp.
13	11 and 12

Ovid MEDLINE(R) and Epub Ahead of Print, In-Process, In-Data-Review & Other Non-Indexed Citations, Daily and Versions

1957

#	Query
1	COVID-19/ or SARS-CoV-2/
2	(COVID or COVID-19* or Coronavirus disease 2019* or Coronavirus 2019* or COVID-2019* or SARS-CoV-2 or SARS CoV 2 or SARS-CoV-2019).ti,ab.
3	1 or 2
4	reinfection/
5	recurrence/
6	(reinfect* or re-infect*).ti,ab.
7	(repeat infection* or repeated infection*).ti,ab.
8	(recurre* adj3 (Covid or Covid-19* or Sars-Cov-2 or PCR or PCRs or RT-PCR or RT-PCRs or positiv*)).ti,ab.
9	((reactivat* or re-activat*) adj5 (Covid or Covid-19* or Sars-Cov-2)).ti,ab.
10	(Re-positiv* or repositiv* or repeat positiv* or repeated positiv*).ti,ab.
11	4 or 5 or 6 or 7 or 8 or 9 or 10
12	3 and 11
13	limit 12 to dt="20200101-20220512"

WHO COVID

2922

(Title, abstract, subject)<br><br>(((ti:(reinfect* OR "re-infect" OR "re-infected" OR "re-infection" OR "re-infections")) OR (ab:(reinfect* OR "re-infect" OR "re-infected" OR "re-infection" OR "re-infections")) OR (ti:("repeat infection" OR "repeat infections" OR "repeated infection" OR "repeated infections")) OR (ab:("repeat infection" OR "repeat infections" OR "repeated infection" OR "repeated infections")) OR (ti:("recurring infection" OR "recurrent infection")) OR (ab:("recurring infection" OR "recurrent infection")) OR (ti:("recurrent Covid" OR "recurrent Covid-19" OR "recurrent Sars-Cov-2" OR "recurrent PCR" OR "recurrent PCRs" OR "recurrent RT-PCR" OR "recurrent RT-PCRs" OR "recurrent positive" OR "recurrent positives" OR "recurrent positivity")) OR (ab:("recurrent Covid" OR "recurrent Covid-19" OR "recurrent Sars-Cov-2" OR "recurrent PCR" OR "recurrent PCRs" OR "recurrent RT-PCR" OR "recurrent RT-PCRs" OR "recurrent positive" OR "recurrent positives" OR "recurrent positivity")) OR (ti:("recurring Covid" OR "recurring Covid-19" OR "recurring Sars-Cov-2" OR "recurring PCR" OR "recurring PCRs" OR "recurring RT-PCR" OR "recurring RT-PCRs" OR "recurring positive" OR "recurring positives" OR "recurring positivity")) OR (ab:("recurring Covid" OR "recurring Covid-19" OR "recurring Sars-Cov-2" OR "recurring PCR" OR "recurring PCRs" OR "recurring RT-PCR" OR "recurring RT-PCRs" OR "recurring positive" OR "recurring positives" OR "recurring positivity")) OR (ti:("reactivate Covid" OR "reactivate Covid-19" OR "reactivate Sars-Cov-2" OR "reactivating Covid" OR "reactivating Covid-19" OR "reactivating Sars-Cov-2" OR "reactivated Covid" OR "reactivated Covid-19" OR "reactivated Sars-Cov-2" OR "reactivation of Covid" OR "reactivation of Covid-19" OR "reactivation of Sars-Cov-2")) OR (ab:("reactivate Covid" OR "reactivate Covid-19" OR "reactivate Sars-Cov-2" OR "reactivating Covid" OR "reactivating Covid-19" OR "reactivating Sars-Cov-2" OR "reactivated Covid" OR "reactivated Covid-19" OR "reactivated Sars-Cov-2" OR "reactivation of Covid" OR "reactivation of Covid-19" OR "reactivation of Sars-Cov-2")) OR (ti:("re-activate Covid" OR "re-activate Covid-19" OR "re-activate Sars-Cov-2" OR "re-activating Covid" OR "re-activating Covid-19" OR "re-activating Sars-Cov-2" OR "re-activated Covid" OR "re-activated Covid-19" OR "re-activated Sars-Cov-2" OR "re-activation of Covid" OR "re-activation of Covid-19" OR "re-activation of Sars-Cov-2")) OR (ab:("re-activate Covid" OR "re-activate Covid-19" OR "re-activate Sars-Cov-2" OR "re-activating Covid" OR "re-activating Covid-19" OR "re-activating Sars-Cov-2" OR "re-activated Covid" OR "re-activated Covid-19" OR "re-activated Sars-Cov-2" OR "re-activation of Covid" OR "re-activation of Covid-19" OR "re-activation of Sars-Cov-2")) OR (ti:(repositiv* OR "re-positive" OR "re-positives" OR "re-positivity" OR "repeated positive" OR "repeated positives" OR "repeat positive" OR "repeat positives" OR "repeat positivity" OR "repeated positivity")) OR (ab:(repositiv* OR "re-positive" OR "re-positives" OR "re-positivity" OR "repeated positive" OR "repeated positives" OR "repeat positive" OR "repeat positives" OR "repeat positivity" OR "repeated positivity"))) AND ((entry_date:2020* OR entry_date:2021* OR entry_date:2022*))) AND la:("en") AND year_cluster:("2021" OR "2020" OR "2022")

Chinese databases: The searches were run on 16/10/2022

Chinese version
三个数据库共计 1695

CNKI

(主题=新型冠状病毒 + 新型冠状病毒感染 + 新型冠状病毒肺炎 + 新型冠状病毒核酸检测) OR (主题=新冠病毒 + 新冠病毒肺炎 + 新冠病毒感染 + 新冠病毒(covid-19) + 新冠病毒检测 + 新冠病毒(sars-cov-2)) OR (主题=covid-19 + covid-19 + covid-19 患者 + covid-19 流行 + covid-19 感染 + covid-19 肺炎) OR (主题=sars-cov-2 + sars-cov-2 + sars-cov-2 病毒 + sars-cov-2 感染)

AND

(全文=重复感染 + 重复感染率) OR (全文=再感染 + 再感染率 + 再感染力) OR (全文=二次感染)

2020-01-01 to 2022-10-16

608 records

Wanfang

(主题:(新型冠状病毒) or 主题:(新冠) or 主题:(新型冠状病毒肺炎) or 主题:(covid-19) or 主题:(sars-cov-2)) and (全部:(重复感染) or 全部:(再次感染) or 全部:(二次感染) or 全部:(再感染)) and Date:2020-2022

中英文扩展、主题词扩展

学科分类：医药、卫生

2020-01-01 to 2022-10-16

936 records

CQvip

((((题名或关键词=新型冠状病毒 OR 题名或关键词=新冠) OR (题名或关键词=covid AND (NOT 题名或关键词=19))) OR ((题名或关键词=sars AND (NOT 题名或关键词=cov)) AND (NOT 题名或关键词=2))) AND (((任意字段=重复感染 OR 任意字段=二次感染) OR 任意字段=再感染) OR 任意字段=再次感染)) AND (years:[2020 TO 2022])

2020-01-01 to 2022-10-16

151 records

English version

A total of 1695 records in three databases.

CNKI

Topic: COVID-19 or SARS-CoV-2 or related terms in database AND

Terms: re-infection or related terms in database;

2020-01-01 to 2022-10-16

608 records

Wanfang

Topic: COVID-19 or SARS-CoV-2, expanding to include related search terms AND

Topic: reinfection, expanding to include related search terms AND

Subject: medicine and health

2020-01-01 to 2022-10-16

936 records

CQvip

Title or Keywords: COVID-19 or SARS-CoV-2 or related terms in database AND

Title or Keywords: reinfection or related terms in the database AND

2020-01-01 to 2022-10-16

151 records

Table S2: The modified PECO(S) framework to formulate the research question and eligibility criteria for inclusion of studies.

Items	Definition
**Population (P)**	Persons with at least one documented episode of SARS-CoV-2 infection confirmed by RT-PCR. If studies included cases ascertained by multiple diagnostic tests, only cases confirmed by PCR were included.
	All age groups
	Regardless of vaccination status
**Exposure (E)**	At least one episode of prior SARS-CoV-2 infection (termed as the initial infection in this review)
**Control (C)**	Not applicable as per the objective of the study
**Context (C)**	All countries
	All settings (general population, healthcare, educational, workplace, and other clinical settings).
	Only studies published from 2020 onwards
**Outcome (O)**	Primary outcome:<br>• Incidence of SARS-CoV-2 reinfection which is defined as any subsequent documented episodes of SARS-CoV-2 infection in persons who have had an initial infection confirmed by RT-PCR tests.<br>• The consecutive two infections are at least 45 days apart.<br>• Regardless of the total number of infections per person.<br>• Regardless of any negative PCR results between the two positive results or any baseline positive serology (antibody test).<br>• During the Pre-omicron period: only reinfection that occurred before the period of Omicron predominance

	Secondary outcomes: The proportion or risk of hospitalization, oxygen supplemental requirement, ICU admission, and death among the reinfected cases.
**Study designs (S)**	Prospective and retrospective cohort studies that report on the cumulative incidence of reinfection or provide data that enable its calculation.
	The control arm of randomized control trials or quasi-experimental studies (individual or cluster) reporting on the cumulative incidence of reinfection.
	Case-control studies if they report relevant data to answer the secondary outcomes.

Table S3: List of studies excluded from meta-analysis of SARS-CoV-2 reinfection during the full-text review phase.

Number	Study first author	Published Year	Title	Reason(s) for Exclusion
1	Abdelrahman	2021	Persistence of symptoms after improvement of acute COVID19 infection, a longitudinal study	no relevant data
2	Abo-Leyah	2021	The protective effect of sars-cov-2 antibodies in scottish healthcare workers	Diagnosis of infection not based on PCR
3	Abu-Raddad	2021	SARS-CoV-2 antibody-positivity protects against reinfection for at least seven months with 95% efficacy	Diagnosis of reinfection not based on PCR
4	Abu-Raddad	2021	Association of Prior SARS-CoV-2 Infection With Risk of Breakthrough Infection Following mRNA Vaccination in Qatar	Time interval to subsequent infection <45 days
5	Abu-Raddad	2021	Introduction and expansion of the SARS-CoV-2 B.1.1.7 variant and reinfections in Qatar: A nationally representative cohort study	Time interval to subsequent infection not reported
6	Addetia	2020	Neutralizing Antibodies Correlate with Protection from SARS-CoV-2 in Humans during a Fishery Vessel Outbreak with a High Attack Rate	Time interval to subsequent infection <45 days

7	Akca	2022	Evaluation of the presence of reinfection in patients presenting to the emergency department with COVID-19 symptoms after recovery	Interval between infections not mentioned
8	Akinbami	2021	Reinfection with SARS-CoV-2 among previously infected healthcare personnel and first responders	Diagnosis of infection not based on PCR
9	Akinbami	2022	Reinfection With Severe Acute Respiratory Syndrome Coronavirus 2 Among Previously Infected Healthcare Personnel and First Responders	Diagnosis of infection not based on PCR
10	Alebouyeh	2021	Re-positive PCR of SARS-CoV-2 in health care persons during COVID-19 pandemic	Diagnosis of infection not based on PCR
11	Alebouyeh	2022	Re-positive PCR of SARS-CoV-2 in health care persons during COVID-19 pandemic	Diagnosis of infection not based on PCR
12	Ali	2021	SARS-CoV-2 reinfection in patients negative for immunoglobulin G following recovery from COVID-19	non eligible study design design
13	Almadhi	2022	Epidemiological assessment of SARS-CoV-2 reinfection	no relevant data
14	Al-Saray	2022	Role of Antibodies against SARS-COV-2 in the Detection of Corona Virus, its Transmissibility and Immunological Status Determination among Different Population in Babylon Province	Diagnosis of infection not based on PCR
15	Alzaabi	2021	Longitudinal changes in IgG levels among COVID-19 recovered patients: A prospective cohort study	Diagnosis of infection not based on PCR
16	Apurva	2022	Comparative study of clinical features and vaccination status in Omicron and non-omicron infected patients during the third wave in Mumbai, India	Omicron period
17	Armagan	2023	COVID-19 disease frequency, risk factors, and re-infection rates in patients with autoimmune rheumatic disease receiving rituximab	small sample size
18	Ashish	2023	Clinical presentation and course of SARS-CoV-2 infection in health-care personnel working in dedicated COVID-19 hospital during 2 pandemic waves in India	Diagnosis of infection not based on PCR

19	Atef	2023	Susceptibility to reinfection with SARS-CoV-2 virus relative to existing antibody concentrations and T cell response	Diagnosis of infection not based on PCR
20	Bakasis	2022	COVID-19: Clinical features and outcomes in unvaccinated 2-dose and 3-dose vaccinated against SARS-CoV-2 patients with systemic autoimmune and autoinflammatory rheumatic diseases	Definition of reinfection is unclear
21	Banham	2021	Hemodialysis Patients Make Long-Lived Antibodies against SARS-CoV-2 that May Be Associated with Reduced Reinfection	Diagnosis of infection not based on PCR
22	Barbui	2021	Long-term follow-up of recovered MPN patients with COVID-19	Wrong outcomes
23	Barzegar	2022	Risk and severity of SARS-CoV-2 reinfection among patients with multiple sclerosis vs. the general population: a population-based study	Diagnosis of infection not based on PCR, study design
24	Bastard	2022	Impact of the Omicron variant on SARS-CoV-2 reinfections in France, March 2021 to February 2022	Diagnosis of infection not based on PCR
25	Bianchi	2023	Burden of COVID-19 disease and vaccine coverages in Apulian splenectomized patients: A retrospective observational study	Definition of reinfection is unclear
26	Biggerstaff	2021	Duration of Viral Nucleic Acid Shedding and Early Reinfection With Severe Respiratory Syndrome Coronavirus 2 in Healthcare Workers and First Responders	no relevant data
27	Bindra	2021	Clinical Presentation and Course of SARS-CoV-2 Infection in Health-Care Personnel Working in Dedicated COVID-19 Hospital During 2 Pandemic Waves in India	Diagnosis of infection not based on PCR
28	Borgonovo	2021	Is COVID-19 severity associated with anti-spike antibody duration? Data from the ARCOVID prospective observational study	Diagnosis of infection not based on PCR
29	Bowe	2022	Acute and postacute sequelae associated with SARS-CoV-2 reinfection	Diagnosis of infection not based on PCR
30	Breathnach	2021	Prior COVID-19 significantly reduces the risk of subsequent infection, but reinfections are seen after eight months	Diagnosis of infection not based on PCR

31	Brown	2022	Treatment of chronic or relapsing COVID-19 in immunodeficiency	no relevant data
32	Brumboiu	2023	Effectiveness and Protection Duration of Anti-COVID-19 Vaccinations among Healthcare Personnel in Cluj-Napoca, Romania	Definition of reinfection is unclear
33	Burkholz	2023	Analysis of well-annotated next-generation sequencing data reveals increasing cases of SARS-CoV-2 reinfection with Omicron	Definition of reinfection is unclear, time interval between 2 infections were not defined
34	Buskermolen	2021	Relapse in the first 8 weeks after onset of COVID-19 disease in outpatients: Viral reactivation or inflammatory rebound?	no relevant data
35	Cai	2023	The Associated Factors of SARS-CoV-2 Reinfection by Omicron Variant - Guangdong Province, China, December 2022 to January 2023	Diagnosis of infection not based on PCR
36	Cao	2020	Post-lockdown SARS-CoV-2 nucleic acid screening in nearly ten million residents of Wuhan, China	non eligible study design design
37	Carazo	2023	Protection against omicron (B.1.1.529) BA.2 reinfection conferred by primary omicron BA.1 or pre-omicron SARS-CoV-2 infection among health-care workers with and without mRNA vaccination: a test-negative case-control study	Interval between infections less than 45 days
38	Casado	2022	Risk of SARS-CoV-2 Reinfections in a Prospective Inception Cohort Study: Impact of COVID-19 Vaccination	Diagnosis of infection not based on PCR
39	Cavanaugh	2021	Reduced Risk of Reinfection with SARS-CoV-2 After COVID-19 Vaccination - Kentucky, May-June 2021	Diagnosis of infection not based on PCR
40	Cerqueira-Silva	2022	Effectiveness of CoronaVac, ChAdOx1 nCoV-19, BNT162b2, and Ad26.COV2.S among individuals with previous SARS-CoV-2 infection in Brazil: a test-negative, case-control study	Diagnosis of infection not based on PCR
41	Cerutti	2020	Clinical immunity in discharged medical patients withCOVID-19	non eligible study design design
42	Chaklader	2022	SARS CoV-2 Re infection after natural infection compared with previously sero-negative: Descriptive longitudinal study	Diagnosis of infection not based on PCR

43	Chan	2021	Development of antibody response to SARS-CoV-2 following asymptomatic infection in patients with plasma cell disorders on immunomodulatory therapy	non eligible study design design
44	Chawki	2022	Long-term impact of COVID-19 among maintenance haemodialysis patients	Diagnosis of infection not based on PCR
45	Chemaitelly	2022	Protection from previous natural infection compared with mRNA vaccination against SARS-CoV-2 infection and severe COVID-19 in Qatar: a retrospective cohort study	Diagnosis of infection not based on PCR
46	Chemaitelly	2022	Duration of immune protection of SARS-CoV-2 natural infection against reinfection	Diagnosis of infection not based on PCR
47	Chen	2020	Clinical course and risk factors for recurrence of positive SARS-CoV-2 RNA: a retrospective cohort study from Wuhan, China	Time interval to subsequent infection <45 days
48	Ciuffreda	2023	Reinfection rate and disease severity of the BA.5 Omicron SARS-CoV-2 lineage compared to previously circulating variants of concern in the Canary Islands (Spain)	no relevant data
49	Clarke	2021	Longevity of SARS-CoV-2 immune responses in hemodialysis patients and protection against reinfection	Diagnosis of infection not based on PCR
50	Cohen	2021	Antibody Status, Disease History, and Incidence of SARS-CoV-2 Infection Among Patients on Chronic Dialysis	Diagnosis of infection not based on PCR
51	Cohen	2022	SARS-CoV-2 incidence, transmission, and reinfection in a rural and an urban setting: results of the PHIRST-C cohort study, South Africa, 2020-21	Diagnosis of infection not based on PCR
52	Cokic	2023	Three Outbreaks of COVID-19 in a Single Nursing Home over Two Years of the SARS-CoV-2 Pandemic	Diagnosis of infection not based on PCR
53	Coleman	2022	Implementation of novel and conventional outbreak control measures in managing COVID-19 outbreaks in a large UK prison	no relevant data
54	Collarino	2022	Persistence of Neutralizing Antibodies and Clinical Protection up to 12 Months After Severe Acute Respiratory Syndrome Coronavirus 2 Infection in the Elderly	Diagnosis of infection not based on PCR

55	Comba	2022	Clinical Characteristics and Outcomes of Patients With SARS-CoV-2 Reinfection	Diagnosis of infection not based on PCR unclear
56	Coppola	2021	Durability of Humoral Immune Responses to SARS-CoV-2 in Citizens of Ariano Irpino (Campania, Italy): A Longitudinal Observational Study With an 11.5-Month Follow-Up	Diagnosis of infection not based on PCR
57	Corrao	2022	Protective action of natural and induced immunization against the occurrence of delta or alpha variants of SARS-CoV-2 infection: a test-negative case-control study	no relevant data
58	Cortellini	2022	Natural immunity to SARS-CoV-2 and breakthrough infections in vaccinated and unvaccinated patients with cancer	Diagnosis of infection not based on PCR unclear, time interval not defiined
59	Daga	2021	SARS-CoV-2 Immunoglobulin g (IgG) Kinetics in Healthcare Workers and Their Close Contacts Reduced Risk of Re-infection: South-East Asian Region	Preprint/ grey literature
60	Dai	2022	Identification of severe acute respiratory syndrome coronavirus 2 breakthrough infections by anti-nucleocapsid antibody among fully vaccinated non-healthcare workers during the transition from the delta to omicron wave	Definition of reinfection is unclear
61	daSilva	2022	Cluster of SARS-CoV-2 Gamma Variant Infections, Parintins, Brazil, March 2021	non eligible study design design
62	de La Vega	2023	SARS-CoV-2 infection-induced immunity reduces rates of reinfection and hospitalization caused by the Delta or Omicron variants	Diagnosis of infection not based on PCR
63	deMalherbe	2022	COVID-19 Prevalence in UNIVI Group Nursing Homes and Multilevel Geriatric Hospitals: Epidemiological Study of Immunological Status with Rapid Serological Tests for Diagnostic Guidance and Follow Up	Diagnosis of infection not based on PCR
64	Deshpande	2021	Longitudinal clinico-serological analysis of anti-nucleocapsid and anti-receptor binding domain of spike protein antibodies against SARS-CoV-2	no relevant data

65	Dhar	2021	Genomic characterization and epidemiology of an emerging SARS-CoV-2 variant in Delhi, India	Diagnosis of reinfection not based on PCR
66	Dimeglio	2021	Protection of Healthcare Workers Against Severe Acute Respiratory Syndrome Coronavirus 2 (SARS-CoV-2) Reinfection	Diagnosis of infection not based on PCR
67	Divya	2021	Development and longevity of antibodies against SARS-CoV-2 in kidney transplant recipients after symptomatic COVID-19	Time interval to subsequent infection <45 days
68	Dominech-Montoliu	2021	Persistence of Anti-SARS-CoV-2 Antibodies Six Months After Infection in an Outbreak With Five Hundred COVID-19 Cases in Borriana (Spain): A Prospective Cohort Study	Preprint/ grey literature
69	Dong	2021	Retrospective analysis on the clinical characteristics of patients who were reinfected with the Corona virus in 2019	Time interval to subsequent infection <45 days
70	Dong	2021	Comprehensive Characterization of COVID-19 Patients with Repeatedly Positive SARS-CoV-2 Tests Using a Large U.S. Electronic Health Record Database	Small sample size
71	Edelstein	2022	Antibody-Mediated Immunogenicity Against Severe Acute Respiratory Syndrome Coronavirus 2 (SARS-CoV-2) Following Priming, Boosting, and Hybrid Immunity: Insights From 11 Months of Follow-up of a Healthcare Worker Cohort in Israel, December 2020-October 2021	Diagnosis of infection not based on PCR
72	Ernst	2023	A Molecular and Epidemiological Investigation of a Large SARS-CoV-2 Outbreak in a Long-Term Care Facility in Luxembourg, 2021	Small sample size
73	Flury	2022	Risk and symptoms of COVID-19 in health professionals according to baseline immune status and booster vaccination during the Delta and Omicron waves in Switzerland-A multicentre cohort study	Diagnosis of infection not based on PCR
74	Gazit	2022	The Incidence of SARS-CoV-2 Reinfection in Persons With Naturally Acquired Immunity With and Without Subsequent Receipt of a Single Dose of BNT162b2 Vaccine	Time interval to subsequent infection not reported

75	Gazit	2022	Severe Acute Respiratory Syndrome Coronavirus 2 (SARS-CoV-2) Naturally Acquired Immunity versus Vaccine-induced Immunity, Reinfections versus Breakthrough Infections: A Retrospective Cohort Study	Time interval to subsequent infection not reported
76	Gazit	2023	Hybrid immunity against reinfection with SARS-CoV-2 following a previous SARS-CoV-2 infection and single dose of the BNT162b2 vaccine in children and adolescents: a target trial emulation	Diagnosis of infection not based on PCR
77	Goldberg	2021	Protection and waning of natural and hybrid COVID-19 immunity	Preprint/ grey literature
78	Goldberg	2022	Protection of previous SARS-CoV-2 infection is similar to that of BNT162b2 vaccine protection: A three-month nationwide experience from Israel	no relevant data
79	Goldberg	2022	Protection and Waning of Natural and Hybrid Immunity to SARS-CoV-2	No relevant data
80	Gong	2021	Characteristics of Viral Shedding in Respiratory Samples and Specific Antibodies Production in 564 COVID-19 Patients	Time interval to subsequent infection <45 days
81	Gonzales	2023	Durability and extent of protection of SARS-CoV-2 antibodies among patients with COVID-19 in Metro Manila, Philippines	Diagnosis of infection not based on PCR for reinfection
82	Good	2022	Severe Acute Respiratory Syndrome Coronavirus 2 (SARS-CoV-2) Infections and Reinfections Among Fully Vaccinated and Unvaccinated University Athletes—15 States, January–November 2021	Diagnosis of infection not based on PCR
83	Graham	2021	Changes in symptomatology, reinfection, and transmissibility associated with the SARS-CoV-2 variant B.1.1.7: an ecological study	Diagnosis of infection not based on PCR
84	Griffante	2021	Persistence of Neutralizing Antibodies to SARS-CoV-2 in First Wave Infected Individuals at Ten Months Post-Infection: The UnIRSA Cohort Study	no relevant data
85	Guevara	2022	Occurrence of SARS-CoV-2 reinfections at regular intervals in Ecuador	Interval between infections less than 45 days

86	Hall	2022	Protection against SARS-CoV-2 after Covid-19 Vaccination and Previous Infection	Diagnosis of reinfection not based on PCR
87	Hamed	2021	Rates of recurrent positive SARS-CoV-2 swab results among patients attending primary care in Qatar	Time interval to subsequent infection <45 days
88	Hanrath	2021	Prior SARS-CoV-2 infection is associated with protection against symptomatic reinfection	Diagnosis of infection not based on PCR
89	Harvey	2021	Association of SARS-CoV-2 Seropositive Antibody Test with Risk of Future Infection	Wrong outcomes
90	Havervall	2021	Robust humoral and cellular immune responses and low risk for reinfection at least 8 months following asymptomatic to mild COVID-19	Diagnosis of infection not based on PCR
91	Havervall	2022	Robust humoral and cellular immune responses and low risk for reinfection at least 8 months following asymptomatic to mild COVID-19	Diagnosis of infection not based on PCR
92	Hawken	2022	Longitudinal SARS-CoV-2 Testing among the Unvaccinated Is Punctuated by Intermittent Positivity and Variable Rates of Increasing Cycle Threshold Values	Time interval to subsequent infection <45 days
93	He	2020	Positive RT-PCR Test Results in 420 Patients Recovered From COVID-19 in Wuhan: An Observational Study	Time interval to subsequent infection <45 days
94	Henein	2021	Obesity strongly predicts covid-19-related major clinical adverse events in coptic clergy	no relevant data
95	Hicks	2021	Characteristics of Persons with Secondary Detection of SARS-CoV-2 >=90 days After First Detection - New Mexico, 2020	Diagnosis of infection not based on PCR
96	Hoang	2021	Re-positive testing, clinical evolution and clearance of infection: results from COVID-19 cases in isolation in Viet Nam	Time interval to subsequent infection <45 days
97	Hu	2023	Characteristics of patients with SARS-COV-2 PCR re-positivity after recovering from COVID-19	no relevant data

98	Huang	2020	Kinetics of SARS-CoV-2 positivity of infected and recovered patients from a single center	Time interval to subsequent infection <45 days
99	Huang	2022	Elevated levels of fructosamine are independently associated with SARS-CoV-2 reinfection: A 12-mo follow-up study	Interval between infections not mentioned
100	Hurtado	2022	Reinfection by SARS CoV2 in Valle Del Cauca, Colombia: A Descriptive Retrospective Study	Diagnosis of infection not based on PCR
101	Hussein	2021	The Risk of SARS-CoV-2 Reinfection in Duhok city, Kurdistan Region of Iraq	Scientific letter
102	Iacute	2021	Sociodemographic and Clinical Features of COVID-19 Reinfection Cases Among Healthcare Workers in Turkey	Preprint/ grey literature
103	Iversen	2022	Seroprevalence of SARS-CoV-2 antibodies and reduced risk of reinfection through 6 months: a Danish observational cohort study of 44 000 healthcare workers	Diagnosis of infection not based on PCR
104	Iyer	2022	COVID-19 outcomes in persons with multiple sclerosis treated with rituximab	Diagnosis of reinfection not based on PCR
105	Jain	2022	Seroprevalence of SARS-CoV-2 among potential convalescent plasma donors and analysis of their deferral pattern: Experience from tertiary care hospital in western India	Diagnosis of infection not based on PCR
106	Jaspe	2021	Introduction and rapid dissemination of SARS-CoV-2 Gamma Variant of Concern in Venezuela	non eligible study design design
107	Jeffery-Smith	2021	Antibodies to SARS-CoV-2 protect against re-infection during outbreaks in care homes, September and October 2020	Diagnosis of infection not based on PCR
108	Katzenstein	2022	Outcome of SARS-CoV-2 infection among patients with common variable immunodeficiency and a matched control group: A Danish nationwide cohort study	Diagnosis of infection not based on PCR
109	Kazanci	2023	COVID-19 infection in children with leukemia: a single-center retrospective study	Interval between infections not mentioned

110	Keeling	2023	Patterns of reported infection and reinfection of SARS-CoV-2 in England	Diagnosis of infection not based on PCR
111	Kim	2021	Viral Shedding with Recurrent SARS-CoV-2 Positive Cases in the Republic of Korea	Preprint/ grey literature
112	Kim	2021	Viral Shedding among Re-Positive Severe Acute Respiratory Syndrome Coronavirus-2 Positive Individuals in Republic of Korea	Time interval to subsequent infection <45 days
113	Kim	2021	Neutralizing Antibody Responses to SARS-CoV-2 in Korean Patients Who Have Recovered from COVID-19	non eligible study design design
114	Klaser	2021	COVID-19 due to the B.1.617.2 (Delta) variant compared to B.1.1.7 (Alpha) variant of SARS-CoV-2: two prospective observational cohort studies	Preprint/ grey literature
115	Klaser	2022	COVID-19 due to the B.1.617.2 (Delta) variant compared to B.1.1.7 (Alpha) variant of SARS-CoV-2: a prospective observational cohort study	Diagnosis of infection not based on PCR
116	Kohler	2021	Impact of baseline SARS-CoV-2 antibody status on syndromic surveillance and the risk of subsequent COVID-19-a prospective multicenter cohort study	Diagnosis of reinfection not based on PCR
117	Kojima	2022	Incidence of SARS-CoV-2 infection among previously infected or vaccinated employees	Time interval to subsequent infection <45 days
118	Krutikov	2021	Incidence of SARS-CoV-2 infection according to baseline antibody status in staff and residents of 100 long-term care facilities (VIVALDI): a prospective cohort study	Diagnosis of infection not based on PCR
119	Kucinskaite-Kodze	2021	Persistence of SARS-CoV-2-Specific Antibodies for 13 Months after Infection	no relevant data
120	Kute	2021	A Multicenter Cohort Study of Indian Centers on Reoccurring SARS-CoV-2 Infections in Kidney Transplant Recipients	non eligible study design design
121	Lawandi	2022	Suspected Severe Acute Respiratory Syndrome Coronavirus 2 (SARS-COV-2) Reinfections: Incidence, Predictors, and Healthcare	Brief report

			Use Among Patients at 238 US Healthcare Facilities, 1 June 2020 to 28 February 2021	
122	Lee	2021	Clinical and Laboratory Findings in Patients With Potential Severe Acute Respiratory Syndrome Coronavirus 2 (SARS-CoV-2) Reinfection, May-July 2020	Time interval to subsequent infection <45 days
123	Lee	2021	Follow-up of hospitalized adults with post-acute COVID-19 syndrome	no relevant data
124	Leidi	2022	Occupational risk of SARS-CoV-2 infection and reinfection during the second pandemic surge: a cohort study	Diagnosis of infection not based on PCR
125	Leidi	2022	Risk of Reinfection After Seroconversion to Severe Acute Respiratory Syndrome Coronavirus 2 (SARS-CoV-2): A Population-based Propensity-score Matched Cohort Study	Diagnosis of infection not based on PCR
126	Leon	2022	COVID-19 Cases and Hospitalizations by COVID-19 Vaccination Status and Previous COVID-19 Diagnosis - California and New York, May-November 2021	Diagnosis of infection not based on PCR
127	Leshem	2021	Population immunity and vaccine protection against infection	non eligible study design design
128	Letizia	2021	SARS-CoV-2 seropositivity and subsequent infection risk in healthy young adults: a prospective cohort study	Time interval to subsequent infection <45 days
129	Levi	2021	Dynamics of SARS-CoV-2 Variants of Concern in Brazil, Early 2021	non eligible study design design
130	Levin-Rector	2022	Reduced odds of Severe acute respiratory syndrome coronavirus 2 reinfection after vaccination among New York City adults, July 2021-November 2021	Diagnosis of infection not based on PCR
131	Levin-Rector	2023	Reduced Odds of Severe Acute Respiratory Syndrome Coronavirus 2 Reinfection After Vaccination Among New York City Adults, July 2021-November 2021	diagnostic test other than PCR
132	Lewis	2022	Effectiveness Associated With Vaccination After COVID-19 Recovery in Preventing Reinfection	diagnostic test other than PCR

133	Li	2021	Analysis of Symptomology, Infectiveness, and Reinfections between Male and Female COVID-19 Patients: Evidence from Japanese Registry Data	Time interval to subsequent infection <45 days
134	Li	2021	Eosinophil: A Nonnegligible Predictor in COVID-19 Re-Positive Patients	Time interval to subsequent infection <45 days
135	Li	2022	Factors Associated with SARS-CoV-2 Repeat Positivity - Beijing, China, June-September 2020	Time interval to subsequent infection <45 days
136	Li	2022	Adverse Events Associated with Nirmatrelvir/Ritonavir: A Pharmacovigilance Analysis Based on FAERS	cut-off duration <45 days or not reported
137	Lin	2023	Effects of COVID-19 vaccination and previous SARS-CoV-2 infection on omicron infection and severe outcomes in children under 12 years of age in the USA: an observational cohort study	diagnostic test other than PCR
138	Lin	2022	Association of Primary and Booster Vaccination and Prior Infection with SARS-CoV-2 Infection and Severe COVID-19 Outcomes	diagnostic test other than PCR
139	Liotti	2021	Assessment of SARS-CoV-2 RNA Test Results Among Patients Who Recovered From COVID-19 With Prior Negative Results	Time interval to subsequent infection <45 days
140	Liu	2020	Recurrent positive SARS-CoV-2: Immune certificate may not be valid	Time interval to subsequent infection <45 days
141	Liu	2021	Clinical characteristics and follow-up analysis of 324 discharged COVID-19 patients in Shenzhen during the recovery period	Time interval to subsequent infection <45 days
142	Longo	2021	COVID-19 prevalence and mortality in a large italian hemoglobinopathies cohort	Diagnosis of infection not based on PCR
143	Lopez-Azor	2023	Triple Positive Effect? Impact of Vaccination, Early Remdesivir Treatment and Omicron Expansion on the Evolution of Covid-19 Infection in Heart Transplant Recipients	review, commentary or not a study article
144	Losada-Castillo	2022	[Impact of COVID-19 pandemic in terms of incidence and lethality in nursing homes in Galicia (Spain)]	language

145	Louis	2022	Early SARS-CoV-2 reinfections within 60 days highlight the need to consider antigenic variations together with duration of immunity in defining retesting policies	Pre-print or grey literature
146	Lusida	2022	Viral shedding and the durability of immunoglobulin G antibodies to severe acute respiratory syndrome coronavirus 2	no relevant data
147	Ma	2023	Trends in Laboratory-Confirmed SARS-CoV-2 Reinfections and Associated Hospitalizations and Deaths Among Adults Aged >=18 Years - 18 U.S. Jurisdictions, September 2021-December 2022	diagnostic test other than PCR
148	Mack	2022	Severe Acute Respiratory Syndrome Coronavirus 2 Reinfection: A Case Series From a 12-Month Longitudinal Occupational Cohort	sample size
149	Mahfuz	2022	COVID-19 among staff and their family members of a healthcare research institution in Bangladesh between March 2020 and April 2021: a test-negative case-control study	cut-off duration <45 days or not reported
150	Maier	2021	Clinical spectrum of SARS-CoV-2 infection and protection from symptomatic re-infection	Diagnosis of infection not based on PCR
151	Maier	2023	SARS-CoV-2 infection-induced immunity and the duration of viral shedding: Results from a Nicaraguan household cohort study	no relevant data/ wrong outcomes
152	Malhotra	2022	COVID-19 infection, and reinfection, and vaccine effectiveness against symptomatic infection among health care workers in the setting of omicron variant transmission in New Delhi, India	Omicron
153	Manica	2022	Risk of Symptomatic Infection During a Second Coronavirus Disease 2019 Wave in Severe Acute Respiratory Syndrome Coronavirus 2-Seropositive Individuals	Diagnosis of infection not based on PCR
154	Manley	2023	SARS-COV-2 REINFECTION AMONG MAINTENANCE DIALYSIS PATIENTS: REPORT OF 471 CASES	review, commentary or not a study article
155	Marc	2022	Occurrence and significance of Omicron BA.1 infection followed by BA.2 reinfection	Pre-print or grey literature

156	Marincu	2022	Clinical Characteristics and Outcomes of COVID-19 Hospitalized Patients: A Comparison between Complete mRNA Vaccination Profile and Natural Immunity	Time interval to subsequent infection <45 days
157	Masia	2021	Incidence of delayed asymptomatic COVID-19 recurrences in a 6-month longitudinal study	Time interval to subsequent infection <45 days
158	Mathew	2023	Clinico-epidemiological profile and outcome of infected health care workers during the three consecutive waves of COVID-19 pandemic: a longitudinal cohort study	cut-off duration <45 days or not reported, unclear definition for reinfection
159	Medic	2023	Incidence, Risk, and Severity of SARS-CoV-2 Reinfections in Children and Adolescents Between March 2020 and July 2022 in Serbia	diagnostic test other than PCR
160	Medic	2022	Risk and severity of SARS-CoV-2 reinfections during 2020-2022 in Vojvodina, Serbia: A population-level observational study	diagnostic test other than PCR
161	Mehboob	2022	Incidence of SARS-CoV-2 re-infection in anti-nucleocapsid IgG-positive healthcare workers: a prospective cohort study	Diagnosis of infection not based on PCR
162	Mellou	2022	Rise of COVID-19 Re-infections as Omicron Variant Prevailed: Implications on Monitoring the Course of the Pandemic	review, commentary or not a study article
163	Mencacci	2022	Immediate reinfection with Omicron variant after clearance of a previous SARS-CoV-2 infection	cut-off duration <45 days or not reported
164	Merzon	2022	Clinical and Socio-Demographic Variables Associated with the Diagnosis of Long COVID Syndrome in Youth: A Population-Based Study	cut-off duration <45 days or not reported
165	Miao	2022	Effects of SARS-CoV-2 Vaccination on Outcomes of COVID-19 in ESKD Patients on Dialysis	no relevant data/ wrong outcomes
166	Monaco	2022	SARS-CoV-2 and Its Variants in Thrice-Infected Health Workers: A Case Series from an Italian University Hospital	sample size
167	Montes-Gonzalez	2023	Protection of hybrid immunity against SARS-CoV-2 reinfection and severe COVID-19 during periods of Omicron variant predominance in Mexico	diagnostic test other than PCR

168	Moradveisi	2021	Evaluation of Anti-SARS-CoV-2 IgG Antibody in Healthcare Professionals Infected with COVID-19	Diagnosis of reinfection not based on PCR
169	Morawiec	2023	Reinfections from SARS-CoV-2: A Retrospective Study from the Gyncentrum Genetic Laboratory in Sosnowiec, Poland, April 2020 to July 2022	no relevant data/ wrong outcomes
170	Morris	2022	Large Scale SARS-CoV-2 Molecular Testing and Genomic Surveillance Reveal Prolonged Infections, Protracted RNA shedding, and Viral Reinfections	non eligible study design design
171	Morris	2022	Re-Infection with SARS-CoV-2 in Solid-Organ Transplant Recipients: Incidence Density and Convalescent Immunity Prior to Re-Infection	Abstract, not full article
172	Morris	2022	SARS-CoV-2 reinfections during the Delta and Omicron waves	Wrong outcomes, no relevant data (no denominator)
173	Moschetta	2021	COVID-19 Reinfection by the Gamma Variant in Kidney Transplant Recipients	non eligible study design design
174	Muir	2021	Neutralizing Antibody Responses After SARS-CoV-2 Infection in End-Stage Kidney Disease and Protection Against Reinfection	Diagnosis of infection not based on PCR
175	Mukherjee	2021	SARS-CoV-2 re-infection: development of an epidemiological definition from India	Diagnosis of infection not based on PCR
176	Mumoli	2020	Clinical immunity in discharged medical patients with COVID-19	Time interval to subsequent infection <45 days
177	Munir	2022	Impact of Vaccination on Symptomatology among COVID-19 Infection and Re-infection	cut-off duration <45 days or not reported
178	Munoz-Lopez	2023	IDENTIFICATION OF CLINICAL FEATURES ASSOCIATED WITH SARS-CoV-2 REINFECTIONS	no relevant data
179	Murillo-Zamora	2021	Predictors of severe symptomatic laboratory-confirmed SARS-CoV-2 reinfection	Time interval to subsequent infection <45 days
180	Murillo-Zamora	2021	Symptomatic SARS-COV-2 reinfection: healthcare workers and immunosuppressed individuals at high risk	Time interval to subsequent infection <45 days

181	Murillo-Zamora	2022	COVID-19 vaccines provide better protection against related pneumonia than previous symptomatic infection	Diagnosis of infection not based on PCR
182	Murugesan	2021	Protective Effect Conferred by Prior Infection and Vaccination on COVID-19 in a Healthcare Worker Cohort in South India	Preprint/ grey literature
183	Murugesan	2022	Protective effect conferred by prior infection and vaccination on COVID-19 in a healthcare worker cohort in South India	cut-off duration <45 days or not reported
184	Naderi	2022	Recurrent viral RNA positivity and candidiasis findings in hospitalized patients with COVID-19	non eligible study design design
185	Nagao	2023	Incidence of and risk factors for suspected COVID-19 reinfection in Kyoto City: a population-based epidemiological study	diagnostic test other than PCR
186	Narrainen	2021	The protective effect of previous COVID-19 infection in a high-prevalence hospital setting	Diagnosis of infection not based on PCR
187	Naveca	2023	SARS-CoV-2 intra-host diversity, antibody response, and disease severity after reinfection by the variant of concern Gamma in Brazil	study design (case series)/ small sample size
188	Nevejan	2022	Early SARS-CoV-2 Reinfections within 60 Days and Implications for Retesting Policies	cut-off duration <45 days or not reported
189	Nicolete	2022	Epidemiology of COVID-19 after Emergence of SARS-CoV-2 Gamma Variant, Brazilian Amazon, 2020-2021	Diagnosis of reinfection not based on PCR
190	Nitipir	2022	Infection and reinfection with SARS-CoV-2 in cancer patients: A cohort study	Time interval to subsequent infection <45 days
191	Ntziora	2022	Protection of vaccination versus hybrid immunity against infection with COVID-19 Omicron variants among Health-Care Workers	Omicron
192	Nunes	2022	SARS-CoV-2 Omicron Symptomatic Infections in Previously Infected or Vaccinated South African Healthcare Workers	Time interval to subsequent infection <45 days
193	Ochoa-Hein	2022	Significant Rise in SARS-CoV-2 Reinfection Rate in Vaccinated Hospital Workers during the Omicron Wave: A Prospective Cohort Study	diagnostic test other than PCR

194	Page	2021	Large-scale sequencing of SARS-CoV-2 genomes from one region allows detailed epidemiology and enables local outbreak management	Time interval to subsequent infection <45 days
195	Pagnano	2021	COVID-19 in chronic myeloid leukemia patients in Latin America	Diagnosis of infection not based on PCR
196	Palstam	2021	Recurrent sick leave after COVID-19: investigating the first wave of the pandemic in a comprehensive Swedish registry-based study	Diagnosis of infection not based on PCR
197	Pampa-Espinoza	2022	Confirmed Severe Acute Respiratory Syndrome Coronavirus 2 Reinfections After a Second Wave With Predominance of Lambda in Lima and Callao, Peru	diagnostic test other than PCR for first episode, no denominator for PCR test
198	Pan	2021	Clinical characteristics of re-hospitalized COVID-19 patients with recurrent positive SARS-CoV-2 RNA: a retrospective study	Time interval to subsequent infection <45 days
199	Pannu	2023	Adverse Events of SARS-CoV-2 Therapy: A Pharmacovigilance Study of the FAERS Database	cut-off duration <45 days or not reported
200	Papasavas	2021	Seroprevalence of SARS-CoV-2 antibodies, associated epidemiological factors and antibody kinetics among healthcare workers in Connecticut	Diagnosis of infection not based on PCR
201	Parker	2022	SARS-CoV-2 transmission: time to rethink public health strategy	review, commentary or not a study article
202	Patalon	2023	Dynamics of Naturally Acquired Immunity Against Severe Acute Respiratory Syndrome Coronavirus 2 in Children and Adolescents	Wrong outcomes, no relevant data (no denominator)
203	Patwardhan	2020	Sustained Positivity and Reinfection With SARS-CoV-2 in Children: Does Quarantine/Isolation Period Need Reconsideration in a Pediatric Population?	Time interval to subsequent infection <45 days
204	Pecoraro	2022	IL RISCHIO DI REINFEZIONE DA SARS-COV-2 E LE IMPLICAZIONI PER LA DIAGNOSTICA	language
205	Pecoraro	2022	The risk of SARS-CoV-2 reinfection and the diagnostics implications	Cannot access full text

206	Peebles	2023	Pfizer-BioNTech Coronavirus Disease 2019 Vaccine Effectiveness against Severe Acute Respiratory Syndrome Coronavirus 2 Infection among Long-term Care Facility Staff with and without Prior Infection in New York City, January-June 2021	Type of test/ Unclear definition of interval between the two infections is explicit.
207	Peltan	2021	Evaluation of potential COVID-19 recurrence in patients with late repeat positive SARS-CoV-2 testing	Did not report any definite reinfection rates because of the different classification for reinfection
208	Pena	2023	Prevalence of symptoms, comorbidities, and reinfections in individuals infected with Wild-Type SARS-CoV-2, Delta, or Omicron variants: a comparative study in western Mexico	Definition of reinfection is unclear
209	Peng	2021	Clinical course and management of 73 hospitalized moderate patients with COVID-19 outside Wuhan	Time interval to subsequent infection <45 days
210	Peng	2023	What contributes to the re-positive nucleic acid test results for the omicron variant of SARS-CoV-2 in the shelter cabin hospital in Shanghai, China?	Omicron
211	Peretz	2023	Maternal and neonatal outcomes of Covid-19 re-infection in pregnancy: a national observational study in israel	review, commentary or not a study article
212	Petras	2022	The Effectiveness of Post-Vaccination and Post-Infection Protection in the Hospital Staff of Three Prague Hospitals: A Cohort Study of 8-Month Follow-Up from the Start of the COVID-19 Vaccination Campaign (COVANESS)	Wrong study outcomes
213	Phan	2022	Incidence of SARS-CoV-2 Infection during the Omicron Variant Emergence in Southern Vietnam: Prior Infection versus Third-Dose Vaccination	diagnostic test other than PCR
214	Piazza	2022	Who Is at Higher Risk of SARS-CoV-2 Reinfection? Results from a Northern Region of Italy	cut-off duration <45 days or not reported
215	Plumb	2022	Effectiveness of COVID-19 mRNA Vaccination in Preventing COVID-19-Associated Hospitalization Among Adults with Previous SARS-CoV-2 Infection - United States, June 2021-February 2022	Diagnosis of infection not based on PCR

216	Pooja	2023	Infection Transmission Factors and their Perception amongst Healthcare Workers infected with SARS-CoV-2 in a Tertiary Care Hospital	review, commentary or not a study article
217	Powell	2022	Protection against symptomatic infection with delta (B.1.617.2) and omicron (B.1.1.529) BA.1 and BA.2 SARS-CoV-2 variants after previous infection and vaccination in adolescents in England, August, 2021-March, 2022: a national, observational, test-negative, case-control study	wrong outcomes,non eligible study design design
218	Prete	2022	Reinfection by the SARS-CoV-2 Gamma variant in blood donors in Manaus, Brazil	Diagnosis of infection not based on PCR
219	Primorac	2022	Cellular Immunity-The Key to Long-Term Protection in Individuals Recovered from SARS-CoV-2 and after Vaccination	Diagnosis of infection not based on PCR
220	Provencio	2022	Seroprevalence and immunological memory against SARS-CoV-2 in lung cancer patients: the SOLID study	no relevant data
221	Pulliam	2022	Increased risk of SARS-CoV-2 reinfection associated with emergence of Omicron in South Africa	Diagnosis of infection not based on PCR
222	Pusdekar	2022	Risk factors for novel corona virus (COVID-19) re-infections among health care workers at tertiary care center: A case control study	Cannot access full text
223	Quattrocchi	2022	Effect of vaccination on SARS-CoV-2 reinfection risk: a case-control study in the Republic of Cyprus	Diagnosis of infection not based on PCR
224	Rahi	2023	Vaccination coverage and breakthrough infections of COVID-19 during the second wave among staff of selected medical institutions in India	cut-off duration <45 days or not reported
225	Ramos	2022	Hospitalization, death, and probable reinfection in Peruvian healthcare workers infected with SARS-CoV-2: a national retrospective cohort study	diagnostic test other than PCR
226	Rennert	2022	Risk of Severe Acute Respiratory Syndrome Coronavirus 2 (SARS-CoV-2) Reinfection in a University Student Population	Diagnosis of infection not based on PCR

227	Reynolds	2023	Risk of and duration of protection from SARS-CoV-2 reinfection assessed with real-world data	diagnostic test other than PCR
228	Rodriguez-Grande	2022	Systematic Genomic and Clinical Analysis of Severe Acute Respiratory Syndrome Coronavirus 2 Reinfections and Recurrences Involving the Same Strain	non eligible study design design, and the detection of reinfection was not similar in comparison with other studies included in the systematic review and meta-analysis.
229	Rothberg	2022	Protection against the omicron variant offered by previous severe acute respiratory syndrome coronavirus 2 infection: a retrospective cohort study	Omicron
230	Ruff	2022	Rapid Increase in Suspected SARS-CoV-2 Reinfections, Clark County, Nevada, USA, December 2021	Omicron
231	Saad	2022	Long-term Consequences, Chances of Re-infection, and Outcomes among Cases Recovered with Severe COVID-19 at a Tertiary Care Centre in Central India	Unclear definition of reinfection/ time interval, wrong outcomes
232	Sabetian	2021	High Post-infection Protection after COVID-19 Among Healthcare Workers: A Population-Level Observational Study Regarding SARS-CoV-2 Reinfection, Reactivation, and Re-positivity and its Severity	Preprint/ grey literature
233	Sabino	2021	Resurgence of COVID-19 in Manaus, Brazil, despite high seroprevalence	non eligible study design design
234	Sacco	2022	Risk and protective factors for SARS-CoV-2 reinfections, surveillance data, Italy, August 2021 to March 2022	diagnostic test other than PCR
235	Sadr	2021	SARS-CoV-2 re-positivity within the first 3 months of COVID-19 recovery;probable re-infection	Preprint/ grey literature
236	Sadr	2021	Distinguishing repeated polymerase chain reaction positivity from re-infections in COVID-19	Small sample size
237	Salah	2022	Covid-19 in recipients of living donor liver transplantation: a worse or an equivalent outcome?	non eligible study design design

238	Samir	2021	COVID-19 in Egypt after a year: the first and second pandemic waves from the radiological point of view; multi-center comparative study on 2000 patients	non eligible study design design
239	Sanchez-Montalva	2021	Risk of sars-cov-2 infection in previously infected and non-infected cohorts of health workers at high risk of exposure	Time interval to subsequent infection <45 days
240	Sanchez-Varela	2022	[Reinfection by the Omicron variant in patients previously infected with the Delta variant of the SARS-CoV-2 coronavirus: An increasingly frequent reality in Primary Care]	language
241	Santeusanio	2021	Kidney transplantation in patients with prior coronavirus disease 2019 (COVID-19)	Time interval to subsequent infection <45 days
242	Santiago-Espinosa	2021	Laboratory-con√Ø¬¨Årmed SARS-CoV-2 reinfection in the population treated at social security	non eligible study design design
243	Schuler	2021	Mild SARS-CoV-2 Illness Is Not Associated with Reinfections and Provides Persistent Spike, Nucleocapsid, and Virus-Neutralizing Antibodies	Time interval to subsequent infection <45 days
244	Segaloff	2021	Risk Factors for Severe Acute Respiratory Syndrome Coronavirus 2 (SARS-CoV-2) Infection and Presence of Anti-SARS-CoV-2 Antibodies among University Student Dormitory Residents, September-November 2020	no relevant data
245	Sevda	2022	Time to reinfection and vaccine breakthrough SARS-CoV-2 infections: a retrospective cohort study	Pre-print or grey literature
246	Shah	2020	Poor outcome and prolonged persistence of SARS-CoV-2 RNA in COVID-19 patients with haematological malignancies; King's College Hospital experience	no relevant data
247	Shankar	2021	Poor Antibody Responses to SARS-CoV-2 Infection or Vaccination Are Associated With High Re-Infection Rates in Haemodialysis and Renal Transplant Patients	Preprint/ grey literature
248	Shankar	2022	SARS-CoV-2-Specific T Cell Responses Are Not Associated with Protection against Reinfection in Hemodialysis Patients	Diagnosis of infection not based on PCR

249	Shannon	2022	Duration of Protection Against SARS-CoV-2 Reinfection and Associated Risk of Reinfection Assessed with Real-World Data	Pre-print or grey literature
250	Shoaib	2021	The Long-Term Characteristics of Immunity Conferred by COVID-19 Using Antibody Tests	Diagnosis of infection not based on PCR
251	Shui	2020	Characteristics of recovered COVID-19 patients with recurrent positive RT-PCR findings in Wuhan, China: a retrospective study	Time interval to subsequent infection <45 days
252	Sieber	2023	Clinical course of SARS-CoV-2 infections of paediatric patients with cystic fibrosis- a single retrospective centre experience	review, commentary or not a study article
253	Silva	2023	Contamination by COVID-19 in professionals of a reference hospital in Para's state	language
254	Simonenko	2021	Covid-19 management in patients after heart transplantation	Time interval to subsequent infection <45 days
255	Sindu	2023	Prior SARS-CoV-2 infection may not alter the clinical course of COVID-19 in lung transplant recipients: A single-center experience	Omicron
256	Singh	2022	Clinico-Epidemiological Characteristics of Healthcare Workers with SARS-CoV-2 Infection during the First and Second Waves in a Teaching Hospital from Eastern India: A Comparative Analysis	diagnostic test other than PCR
257	Singla	2022	Dynamics of SARS-CoV-2 Antibody Response in a Longitudinal Cohort of Healthcare Workers from India	no relevant data
258	Slezak	2021	Rate and severity of suspected SARS-Cov-2 reinfection in a cohort of PCR-positive COVID-19 patients	Research note
259	Smolenov	2022	Impact of previous exposure to SARS-CoV-2 and of S-Trimer (SCB-2019) COVID-19 vaccination on the risk of reinfection: a randomised, double-blinded, placebo-controlled, phase 2 and 3 trial	Diagnosis of infection not based on PCR
260	Smolenov	2022	COVID-19 Reinfection and Disease Severity in the New York City Health + Hospitals System	review, commentary or not a study article

261	Snezana	2022	Risk and severity of SARS-CoV-2 reinfections during 2020-2022 in Vojvodina, Serbia: a population-level study	Pre-print or grey literature
262	Soriano	2021	Main differences between the first and second waves of COVID-19 in Madrid, Spain	Diagnosis of infection not based on PCR
263	Soriano	2021	Third wave of COVID-19 in Madrid, Spain	non eligible study design design
264	Spicer	2022	Protective Immunity after Natural Infection with Severe Acute Respiratory Syndrome Coronavirus-2 (SARS-CoV-2) - Kentucky, USA, 2020	Diagnosis of infection not based on PCR
265	Spicer	2022	Association of Severe Acute Respiratory Syndrome Coronavirus 2 Vaccination or a Prior Positive Test Result in Adolescents during the Delta Variant Surge in Kentucky	diagnostic test other than PCR
266	Sree Sudha	2022	Identification of spectrum of persistent post-COVID-19 symptoms and their duration in central India: a pilot study	Unclear definition of reinfection/ time interval, wrong outcomes
267	Sughayer	2022	Comparison of the effectiveness and duration of anti-RBD SARS-CoV-2 IgG antibody response between different types of vaccines: Implications for vaccine strategies	non eligible study design design
268	Suleyman	2023	Outcomes associated with SARS-CoV-2 reinfection in individuals with natural and hybrid immunity	no relevant data/ wrong outcomes
269	Sullivan	2021	Follow-Up SARS-CoV-2 PCR Testing Outcomes From a Large Reference Lab in the US	non eligible study design design
270	Sullivan	2023	Antibody titer levels and the effect on subsequent SARS-CoV-2 infection in a large US-based cohort	diagnostic test other than PCR
271	Sun	2022	[Clinical characteristics of convalescent children infected with SARS-CoV-2 Omicron variant in Tianjin]	Wrong definition of reinfection
272	Syed	2022	SARS-CoV-2 seropositivity and subsequent infection risk: a prospective cohort study	Diagnosis of infection not based on PCR

273	Tan	2023	Results from a systematic programme of evaluating COVID-19 reinfection cases in the early phase of the pandemic, Singapore	cut-off duration <45 days or not reported
274	Tan	2023	Infectiousness of SARS-CoV-2 breakthrough infections and reinfections during the Omicron wave	Omicron
275	Tan	2022	[Analysis on characteristics and influencing factors of COVID-19 confirmed cases with viral nucleic acid re-positive after discharge in Guangdong Province]	Wrong definition of reinfection
276	Tanunliong	2021	Persistence of Anti-SARS-CoV-2 Antibodies in Long Term Care Residents Over Seven Months After Two COVID-19 Outbreaks	Diagnosis of infection not based on PCR
277	Thompson	2021	Rapid Emergence and Epidemiologic Characteristics of the SARS-CoV-2 B.1.526 Variant - New York City, New York, January 1-April 5, 2021	Diagnosis of infection not based on PCR
278	Tomanoski	2023	WCN23-0981 RISK FACTORS FOR COVID-19 MORTALITY IN HEMODIALYSIS PATIENTS	review, commentary or not a study article
279	Tomassini	2021	Setting the criteria for SARS-CoV-2 reinfection - six possible cases	non eligible study design design
280	Tomic*	2021	Divergent trajectories of antiviral memory after SARS-Cov-2 infection	Preprint/ grey literature
281	Trujillo	2023	Predictors of Recurrent Laboratory-Confirmed Symptomatic SARS-CoV-2 Infections in a Cohort of Healthcare Workers	diagnostic test other than PCR
282	Tu	2023	SARS-CoV-2 Infection, Hospitalization, and Death in Vaccinated and Infected Individuals by Age Groups in Indiana, 2021–2022	cut-off duration <45 days or not reported
283	Turkkan	2021	COVID-19 in lung transplant recipients: A single-center experience	non eligible study design design
284	Tzitzi	2023	Casivirimab/Imdevimab Effect on COVID-19 Outcome and Reinfection in a Real-World SARS-COV-2 Variant Transition Period Setting	Cannot access full text

285	Umar	2021	SARS-CoV-2 spike antibody levels trend among Sinopharm vaccinated people	Diagnosis of infection not based on PCR
286	Unsal	2022	Clinical and laboratory outcomes of the solid cancer patients reinfected with SARS-CoV-2	Time interval to subsequent infection <45 days
287	Uysal	2022	Measurement of antibody levels in patients with COVID-19 over time by immunofluorescence assay: a longitudinal observational study	no relevant data
288	Varela	2023	Genomic evidence of SARS-CoV-2 reinfection cases in southern Brazil	Brief report, wrong outcomes
289	Veronica	2021	Incidence of COVID-19 recurrence among large cohort of healthcare employees	Diagnosis of infection not based on PCR
290	Veterini	2021	Probiotics intake as adjunct therapy for infected health-care with sars cov-2	Time interval to subsequent infection <45 days
291	Vicentini	2023	Risk of SARS-CoV-2 reinfection by vaccination status, predominant variant and time from prior infection: a cohort study, Reggio Emilia province, Italy, February 2020 to February 2022	diagnostic test other than PCR
292	Virk	2023	Hybrid Immunity Provides Protective Advantage Over Vaccination or Prior Remote Coronavirus Disease 2019 Alone	Wrong outcomes, no relevant data (no nominator)
293	Vitale	2021	Assessment of SARS-CoV-2 Reinfection 1 Year After Primary Infection in a Population in Lombardy, Italy	Research letter
294	Vo	2022	Robust and Functional Immune Memory Up to 9 Months After SARS-CoV-2 Infection: A Southeast Asian Longitudinal Cohort	no relevant data
295	Wack	2021	No SARS-CoV-2 reinfection among staff health-care workers: Prospective hospital-wide screening during the first and second waves in Paris	Time interval to subsequent infection <45 days
296	Wajiha	2022	Safety Profile of Sinopharm COVID-19 Vaccine and Breakthrough Infections in Pakistan	Pre-print or grey literature

297	Wang	2023	SARS-CoV-2 Reinfection With Different SARS-CoV-2 Variants in Children, Ohio, United States	diagnostic test other than PCR
298	Wei	2020	Re-positive discharged COVID-19 patients are at low transmission risk for SARS-CoV-2 infection,Äì a finding from recovered COVID-19 patients in Wuhan, China	Preprint/ grey literature
299	Wilkins	2021	Serologic status and SARS COV-2 infection over 6-months of follow-up in healthcare workers in Chicago: A cohort study	Diagnosis of infection not based on PCR
300	Wu	2021	A follow-up study shows that recovered patients with re-positive PCR test in Wuhan may not be infectious	Time interval to subsequent infection <45 days
301	Wu	2022	Follow-up of patients with COVID-19 by the Delta variant after hospital discharge in Guangzhou, Guandong, China	Wrong study outcomes
302	Wu	2023	Effect of the incremental protection of previous infection against Omicron infection among individuals with a hybrid of infection- and vaccine-induced immunity: a population-based cohort study in Canada	Omicron period
303	Xu	2020	No evidence of Re-infection or¬†Human-to-Human Transmission¬†in Cured COVID-19 Patients, a Retrospective Cohort Study	Preprint/ grey literature
304	Xu	2020	No Evidence of Re-infection or Person-to-Person Transmission in Cured COVID-19 Patients in Guangzhou, a Retrospective Observational Study	Time interval to subsequent infection <45 days
305	Yang	2022	SARS-CoV-2 in Malaysia: A surge of reinfection during the predominantly Omicron period	diagnostic test other than PCR
306	Ye	2020	Clinical characteristics of severe acute respiratory syndrome coronavirus 2 reactivation	Time interval to subsequent infection <45 days
307	Yi	2021	Risk factors for recurrent positive results of the nucleic acid amplification test for COVID-19 patients: a retrospective study	Time interval to subsequent infection not mentioned
308	Yu	2020	Clinical Characteristics of the Discharged COVID-19 Patients with Re-Positive Nucleic Acid Test	Preprint/ grey literature

309	Yuan	2020	Polymerase Chain Reaction Assays Reverted to Positive in 25 Discharged Patients with COVID-19	Time interval to subsequent infection <45 days
310	Zare	2021	COVID-19 re-infection in Shahroud, Iran: a follow-up study	Time interval to subsequent infection <45 days
311	Zhang	2020	The N gene of SARS-CoV-2 was the main positive component in repositive samples from a cohort of COVID-19 patients in Wuhan, China	no relevant data
312	Zhang	2021	The characteristics of 527 discharged COVID-19 patients undergoing long-term follow-up in China	Time interval to subsequent infection <45 days
313	Zhang	2021	COVID-19 reinfection in the presence of neutralizing antibodies	Time interval to subsequent infection <45 days
314	Zhang	2022	Development and multi-center clinical trials of an up-converting phosphor technology-based point-of-care (UPT-POCT) assay for rapid COVID-19 diagnosis and prediction of protective effects	non eligible study design design
315	Zhang	2023	The Infection of Healthcare Workers and the Reinfection of Patients by Omicron Variant - Jiangsu Province, China, December 2022 to January 2023	Omicron period
316	Zhao	2021	The relationship between SARS-COV-2 RNA positive duration and the risk of recurrent positive	no relevant data
317	Zhao	2022	A comparison of clinical characteristics between COVID-19 patients with and without re-positive nucleic acid test	small sample size
318	Zheng	2020	Incidence, clinical course and risk factor for recurrent PCR positivity in discharged COVID-19 patients in Guangzhou, China: A prospective cohort study	Time interval to subsequent infection <45 days
319	Ziaul	2022	Severity of COVID-19 reinfection and associated risk factors: findings of a cross-sectional study in Bangladesh	Pre-print or grey literature
320	代晓朋, 盖丽娜	2022	新型冠状病毒肺炎出院患者核酸复阳研究进展	review

321	代艳, 唐宁宁	2020	广西儿童新型冠状病毒肺炎 11 例临床分析	no relevant data
322	佟乐, 张崇唯	2020	免疫功能与新型冠状病毒肺炎康复患者核酸检测结果复阳的关系研究	no relevant data
323	傅昭仪	2020	新冠肺炎二次感染,你了解多少?	review/comment
324	刘宁, 娄靖	2020	北京市通州区急救转运新型冠状病毒肺炎病例的临床特征分析	comment
325	刘晓芳,陈美平	2022	17 例新型冠状病毒 Omicron 变异株境外输入感染者的流行病学特征	no relevant data
326	刘洁, 罗万军	2020	91 例儿童新型冠状病毒肺炎确诊病例临床及流行病学特征	no relevant data
327	刘芳, 兰为顺	2020	孕妇感染新型冠状病毒肺炎的临床特点及影像学表现	no relevant data
328	向柯华,姚媛贞	2021	张家界市 76 例新型冠状病毒 Delta 变异株感染病例的临床特征	no relevant data
329	吴小兰	2021	新型冠状病毒肺炎恢复期 PCR 核酸复阳患者临床转归追踪研	no relevant data
330	周灵, 刘威,	2020	出院后新型冠状病毒核酸复阳的新型冠状病毒肺炎患者 11 例临床特征分析	no relevant data
331	姜芬, 丁坤	2021	SARS-CoV-2 核酸复阳患者临床特点及不同标本核酸检测结果分析	no relevant data
332	孔祥亘，布学慧	2020	新型冠状病毒病患者病毒核酸反复阳性超 60 天 2 例报告	no relevant data
333	宋旭辉, 林胜	2020	多部位样本核酸检测持续阳性的新型冠状病毒肺炎患者 1 例分析	no relevant data

334	小林鉄郎	2021	新型コロナウィルスの数理モデル?日本人モ デラーがやったこと?	no relevant data
335	岳勇, 梁娴	2021	新型冠状病毒疫苗接种对成都市境外输入病例流行病学及临床特征的影响分析	no relevant data
336	张华, 龚利强	2021	常熟市 1 例新型冠状病毒肺炎出院复阳病例的调查报告	no relevant data
337	张永栋, 郗爱旗	2020	青海省 18 例确诊新型冠状病毒肺炎流行病学和感染特征	no relevant data
338	张淑香,纳建荣	2021	宁夏回族自治区 2019 冠状病毒病 75 例临床特征及预后研究	no relevant data
339	张莉, 张年萍	2021	大同市新型冠状病毒肺炎患者临床特征分析	no relevant data
340	彭佳华, 周昌静	2022	新型冠状病毒性肺炎核酸"复阳"再入院患者临床特征分析	no relevant data
341	时正雨, 杨铭	2021	成都市 147 例新型冠状病毒感染者的流行病学特征	no relevant data
342	李娇, 毛毅	2022	不同基因型新型冠状病毒感染病例的特征分析	no relevant data
343	李志丽, 李昱	2021	复检核酸阳性的新型冠状病毒感染者分布及传染性特征分析	no relevant data
344	李泽成, 朱继旭	2022	陕西省 2020 年 245 例新型冠状病毒肺炎流行病学特征及其影响因素	no relevant data
345	李浩, 王晓辉	2020	深圳新冠肺炎出院病例核酸复检阳性特征分析	no relevant data
346	李盼盼, 吴彪	2020	61 例新型冠状病毒肺炎患者出院后随访调查分析	no relevant data

347	杨艳娟, 魏晓娴	2021	云南省瑞丽市新型冠状病毒感染/肺炎成人和儿童本土病例的流行病学及临床特征	no relevant data
348	梁继仁, 韦松华	2020	14 例新型冠状病毒肺炎病例流行病学和临床特征	no relevant data
349	梁联哨, 林艳荣	2020	南宁市 50 例新型冠状病毒肺炎疑似病例临床分析	no relevant data
350	梁运光, 谢周华	2020	58 例新型冠状病毒肺炎患者的临床特征及转归	no relevant data
351	欧阳晓春, 文吉秋	2020	21 例新型冠状病毒肺炎核酸持续阳性超过 30 d 患者临床特征分析	no relevant data
352	毛欣, 付士祥	2020	对 23 例新型冠状病毒肺炎患者临床资料的分析	no relevant data
353	汪晓凤, 卢刚	2020	新型冠状病毒肺炎患者实验室检查临床特点分析	no relevant data
354	王伟,李俊	2020	385 例确诊新型冠状病毒肺炎的临床及流行病学特征	no relevant data
355	王少博	2021	165 例新型冠状病毒肺炎的临床特征及重症化的危险因素分析	no relevant data
356	王海峰, 李亚飞	2022	2022 年一起由新型冠状病毒 Delta 变异株引起 365 例感染者的暴发疫情特征	no relevant data
357	王瑞瑞	2022	新型冠状病毒肺炎患者临床特征分析及相关危险因素的探讨	no relevant data
358	田继东, 谢敏	2021	轻症 COVID-19 患儿流行病学和临床特征分析	no relevant data
359	程倩, 陈少云	2021	35 例长时间核酸阳性患者的临床特征及转归分析	no relevant data

360	罗东霞, 刘大凤	2020	成都市 1 例输入性新型冠状病毒肺炎病例诊治及两次复阳过程探讨	no relevant data
361	罗纯, 温学良,	2020	广州地区新型冠状病毒肺炎出院病例核酸再次阳性的临床特征	no relevant data
362	胡敏, 岳勇	2022	成都市复检核酸阳性新冠肺炎患者流行特征分析	no relevant data
363	艾香英, 傅晓霞	2020	30 例新型冠状病毒核酸复阳返院患者的病例特点	no relevant data
364	贾海梅, 王清华	2022	福州市新型冠状病毒感染患者出院后核酸"复阳"情况分析	no relevant data
365	赵文恒, 马钰	2022	新型冠状病毒肺炎患者出院后核酸检测阳性临床特征及原因综述	no relevant data
366	赵磊, 薛剑	2020	石家庄地区新型冠状病毒肺炎患者的临床特征及影像学分析	no relevant data
367	路素坤, 牛波	2021	石家庄地区 133 例儿童 SARS-CoV-2 感染流行病学特征	no relevant data
368	郑文彬,汤建桥	2020	武汉市儿童 2019 新型冠状病毒感染 146 例的临床特征分析	no relevant data
369	陈芳, 赖家佳	2020	重庆地区 172 例新型冠状病毒感染患者的肺部 CT 表现分析	no relevant data
370	雒丽, 张浩利	2020	老年新型冠状病毒肺炎患者的临床特征及护理策略	no relevant data
371	龙秋月,郑雅莉,高占成	2021	新型冠状病毒再感染病例临床特点	review

Note: RT-PCR= Reverse transcriptase-polymerase chain reaction

Table S4: Data mapping of studies included in the systematic review.

			Incidence		Severity				Outcome						
Study	Country	Income group	Proportion	Rate	Mild	Moderate	Severe	Moderate-severe	Oxygenation	Hospitalization	ICU	Ventilation	Mortality	Study design	Minimum cut-off duration
Abu-Raddad et al. (2021)	Qatar	HIC	Yes	Yes	Yes	No	No	No	No	Yes	No	No	Yes	Retrospective	45+ days
Armstrong et al. (2021)	USA	HIC	Yes	No	No	No	No	No	No	No	No	No	Yes	Retrospective	90+ days
Arslan et al. (2021)	Turkey	UMIC	Yes	No	No	No	No	No	No	Yes	No	No	Yes	Retrospective	90+ days
Bean et al. (2021)	USA	HIC	Yes	No	No	No	No	No	No	Yes	Yes	Yes	No	Retrospective	90+ days
Comelli et al. (2021)	Italy	HIC	Yes	No	No	No	No	No	No	No	No	No	No	Retrospective	90+ days
Flacco et al. (2021)	Italy	HIC	Yes	No	No	No	No	No	No	Yes	No	No	Yes	Retrospective	90+ days
Gallais et al. (2021)	France	HIC	Yes	Yes	No	No	No	No	No	No	No	No	No	Prospective	90+ days
Garcia-Abellan et al. (2021)	Spain	HIC	Yes	No	No	No	No	Yes	No	No	No	No	No	Prospective	60+ days
Hall et al. (2021)	UK	HIC	Yes	Yes	No	No	No	No	No	No	No	No	No	Prospective	90+ days
Hansen et al. (2021)	Denmark	HIC	Yes	No	No	No	No	No	No	No	No	No	No	Retrospective	90+ days
Islamoglu et al. (2021)	Turkey	UMIC	Yes	No	No	No	No	No	No	No	No	No	No	Prospective	60+ days
Jeffery-Smith et al. (2021)	UK	HIC	Yes	No	Yes	No	No	No	No	No	No	No	No	Prospective	90+ days
Kim et al. (2021)	USA	HIC	Yes	No	No	No	No	No	No	Yes	Yes	Yes	Yes	Retrospective	90+ days
Konka et al. (2021)	Poland	HIC	Yes	No	No	No	No	No	No	No	No	No	No	Retrospective	60+ days
Lumley et al. (2021)	UK	HIC	Yes	No	No	No	No	No	No	No	No	No	No	Prospective	60+ days
Peghin et al. (2021)	Italy	HIC	Yes	No	Yes	No	No	No	No	No	No	No	No	Prospective	90+ days
Pilz et al. (2021)	Austria	HIC	Yes	No	No	No	No	No	No	Yes	No	No	Yes	Retrospective	90+ days
Qureshi et al. (2021)	USA	HIC	Yes	No	No	No	No	No	No	No	No	Yes	Yes	Retrospective	45+ days
Salehi-Vaziri et al. (2021)	Iran	LMIC	Yes	No	No	Yes	No	No	No	No	No	No	No	Prospective	60+ days
Sheehan et al. (2021)	USA	HIC	Yes	No	No	No	No	No	No	Yes	Yes	Yes	No	Retrospective	90+ days
Abu-Raddad et al. (2022)	Qatar	HIC	Yes	No	No	No	No	No	No	No	No	No	No	Retrospective	90+ days
Akpan et al. (2022)	Liberia	LIC	Yes	No	No	No	No	No	No	No	No	No	No	Retrospective	90+ days
Arslan et al. (2022)	Turkey	UMIC	Yes	No	No	No	No	No	No	Yes	Yes	No	Yes	Retrospective	90+ days
Dhumal et al. (2022)	India	LMIC	Yes	No	No	No	No	No	No	No	No	No	No	Retrospective	45+ days
Hammerman et al. (2022)	Israel	HIC	Yes	Yes	No	No	No	No	No	No	No	No	No	Retrospective	90+ days
Hønge et al. (2022)	Denmark	HIC	Yes	No	No	No	No	No	No	No	No	No	No	Retrospective	90+ days
Malhotra et al. (2022)	India	LMIC	Yes	Yes	No	No	No	No	No	No	No	No	No	Retrospective	90+ days
Mensah et al. (a) (2022)	UK	HIC	Yes	No	No	No	No	No	No	Yes	Yes	No	Yes	Retrospective	90+ days
Mensah et al. (b) (2022)	UK	HIC	Yes	No	No	No	No	No	No	Yes	Yes	No	Yes	Prospective	90+ days
Nguyen et al. (2022)	France	HIC	Yes	No	No	No	No	No	No	Yes	Yes	No	Yes	Retrospective	90+ days
Nisha et al. (2022)	India	LMIC	Yes	No	No	No	No	No	No	No	No	No	No	Retrospective	90+ days
Nordstrom et al. (2022)	Sweden	HIC	Yes	Yes	No	No	No	No	No	No	No	No	No	Retrospective	90+ days
Rahman et al. (2022)	Bangladesh	LMIC	Yes	Yes	No	No	Yes	No	Yes	Yes	Yes	No	No	Prospective	45+ days
Rivelli et al. (2022)	USA	HIC	Yes	Yes	No	No	No	No	No	No	No	No	No	Prospective	90+ days
Ronchini et al. (2022)	Italy	HIC	Yes	No	No	No	No	No	No	No	No	No	No	Prospective	60+ days
Shaheen et al. (2022)	Saudi Arabia	HIC	Yes	No	No	No	No	Yes	No	Yes	Yes	No	No	Retrospective	90+ days
Sheikh et al. (2022)	UK	HIC	Yes	No	No	No	No	No	No	No	No	No	No	Prospective	90+ days
Al-Otaiby et al. (2022)	Saudi Arabia	HIC	Yes	No	No	No	Yes	No	No	No	Yes	No	Yes	retrospective	90+ days
Al Haddad et al. (2022)	Kuwait	HIC	Yes	Yes	Yes	No	Yes	No	No	No	No	No	No	retrospective	45+ days
Ando et al. (2022)	Unites States	HIC	Yes	No	No	No	No	No	No	No	No	No	No	retrospective	90+ days
Aslaner et al. (2022)	Turkey	UMIC	Yes	No	Yes	Yes	Yes	No	No	Yes	Yes	No	Yes	retrospective	45+ days
Attauabi et al. (2021)	Denmark	HIC	Yes	No	Yes	No	Yes	No	No	No	No	No	Yes	Prospective	90+ days
Berec et al. (2022)	Czech	HIC	Yes	No	No	No	No	No	No	No	No	No	No	retrospective	60+ days
Cocchio et al. (2022)	Italy	HIC	Yes	No	No	No	No	No	No	No	No	No	No	retrospective	90+ days
Cohen et al. (2023)	Israel	HIC	Yes	No	No	No	No	No	No	No	No	No	No	Prospective	90+ days
Arriba et al. (2023)	Spain	HIC	Yes	No	No	No	No	No	No	No	No	No	Yes	retrospective	90+ days
Erbas et al. (2023)	Turkey	UMIC	Yes	No	Yes	Yes	Yes	No	No	No	No	No	Yes	retrospective	90+ days
Freire-Neto et al. (2022)	Brazil	UMIC	Yes	No	No	No	No	No	No	No	No	No	No	retrospective	90+ days
Gazit et al. (2022)	Israel	HIC	Yes	No	No	No	No	No	No	Yes	No	No	Yes	retrospective	90+ days
Goldberg et al. (2022)	Israel	HIC	Yes	No	No	No	Yes	No	No	Yes	No	No	Yes	retrospective	90+ days
Guedes et al. (2023)	Brazil	UMIC	Yes	No	Yes	No	No	No	No	Yes	No	No	Yes	Prospective	90+ days
Masia et al. (2022)	Spain	HIC	Yes	No	No	No	No	No	No	Yes	No	No	No	Prospective	90+ days
Michlmayr et al. (2022)	Denmark	HIC	Yes	No	No	No	No	No	No	No	No	No	No	retrospective	90+ days
Nielsen et al. (2022)	Denmark	HIC	Yes	No	No	No	No	No	No	Yes	No	No	Yes	retrospective	90+ days
Özüdogru et al. (2023)	Turkey	UMIC	Yes	No	No	No	No	No	No	No	No	No	No	retrospective	90+ days
Pecoraro et al. (2022)	Italy	HIC	Yes	No	No	No	No	No	No	No	No	No	No	retrospective	90+ days
Reddy et al. (2023)	India	LMIC	Yes	No	Yes	Yes	Yes	No	No	No	No	No	No	Prospective	90+ days
Rennert et al. (2022)	Unites States	HIC	Yes	No	No	No	No	No	No	No	No	No	No	retrospective	90+ days
Rosenberg et al. (2022)	Unites States	HIC	Yes	Yes	No	No	No	No	No	No	No	No	No	retrospective	60+ days
Silva et al. (2023)	Brazil	UMIC	Yes	No	No	No	No	No	No	Yes	No	No	No	Prospective	90+ days
Tavakoli et al. (2023)	Iran	LMIC	Yes	No	No	No	No	No	No	No	No	No	No	retrospective	45+ days
Temiz et al. (2022)	Turkey	UMIC	Yes	No	No	No	No	No	No	Yes	Yes	No	Yes	retrospective	60+ days
Turbett et al. (2023)	Unites States	HIC	Yes	No	No	No	No	No	No	Yes	Yes	No	Yes	retrospective	45+ days
Yuguero et al. (2022)	Spain	HIC	Yes	No	No	No	No	No	No	Yes	No	No	Yes	retrospective	90+ days

Note: HIC: High-Income Countries, UMIC: Upper-middle Income Countries, LMIC: Lower-middle Income Countries, LIC: Lower Income Countries

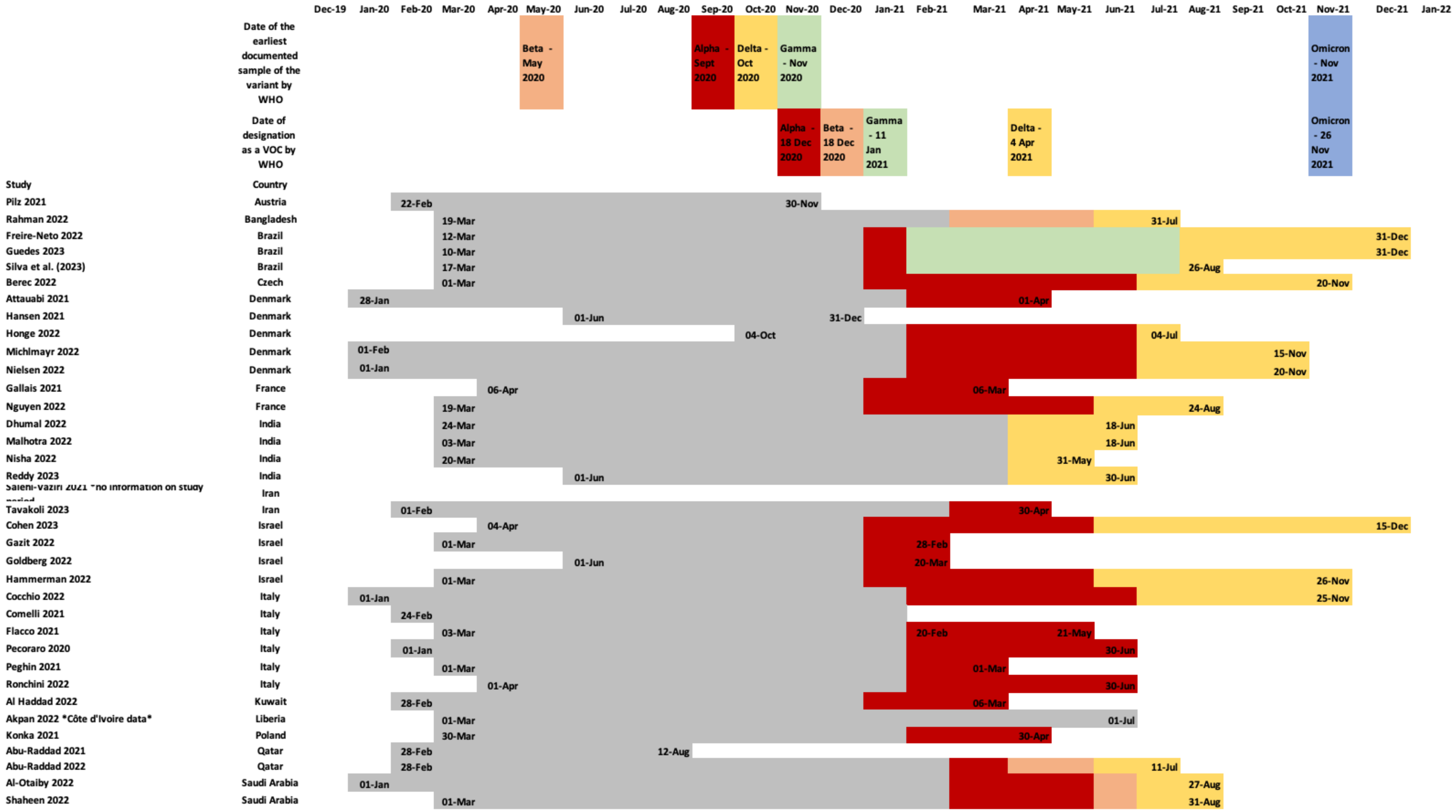

Dec-19 Jan-20 Feb-20 Mar-20 Apr-20 May-20 Jun-20 Jul-20 Aug-20 Sep-20 Oct-20 Nov-20 Dec-20 Jan-21 Feb-21 Mar-21 Apr-21 May-21 Jun-21 Jul-21 Aug-21 Sep-21 Oct-21 Nov-21 Dec-21 Jan-22
Date of the earliest documented sample of the variant by WHO
Beta - May 2020
Alpha - Sept 2020
Delta - Oct 2020
Gamma - Nov 2020
Omicron - Nov 2021
Date of designation as a VOC by WHO
Alpha - 18 Dec 2020
Beta - 18 Dec 2020
Gamma - 11 Jan 2021
Delta - 4 Apr 2021
Omicron - 26 Nov 2021
Study Country
Pilz 2021 Austria 22-Feb 30-Nov
Rahman 2022 Bangladesh 19-Mar 31-Jul
Freire-Neto 2022 Brazil 12-Mar 31-Dec
Guedes 2023 Brazil 10-Mar 31-Dec
Silva et al. (2023) Brazil 17-Mar 26-Aug
Berec 2022 Czech 01-Mar 20-Nov
Attauabi 2021 Denmark 28-Jan 01-Apr
Hansen 2021 Denmark 01-Jun 31-Dec
Honge 2022 Denmark 04-Oct 04-Jul
Michlmayr 2022 Denmark 01-Feb 15-Nov
Nielsen 2022 Denmark 01-Jan 20-Nov
Gallais 2021 France 06-Apr 06-Mar
Nguyen 2022 France 19-Mar 24-Aug
Dhumal 2022 India 24-Mar 18-Jun
Malhotra 2022 India 03-Mar 18-Jun
Nisha 2022 India 20-Mar 31-May
Reddy 2023 India 01-Jun 30-Jun
Salehi-Vaziri 2021 *no information on study period Iran
Tavakoli 2023 Iran 01-Feb 30-Apr
Cohen 2023 Israel 04-Apr 15-Dec
Gazit 2022 Israel 01-Mar 28-Feb
Goldberg 2022 Israel 01-Jun 20-Mar
Hammerman 2022 Israel 01-Mar 26-Nov
Cocchio 2022 Italy 01-Jan 25-Nov
Comelli 2021 Italy 24-Feb
Flacco 2021 Italy 03-Mar 20-Feb 21-May
Pecoraro 2020 Italy 01-Jan 30-Jun
Peghin 2021 Italy 01-Mar 01-Mar
Ronchini 2022 Italy 01-Apr 30-Jun
Al Haddad 2022 Kuwait 28-Feb 06-Mar
Akpan 2022 *Côte d'Ivoire data* Liberia 01-Mar 01-Jul
Konka 2021 Poland 30-Mar 30-Apr
Abu-Raddad 2021 Qatar 28-Feb 12-Aug
Abu-Raddad 2022 Qatar 28-Feb 11-Jul
Al-Otaiby 2022 Saudi Arabia 01-Jan 27-Aug
Shaheen 2022 Saudi Arabia 01-Mar 31-Aug

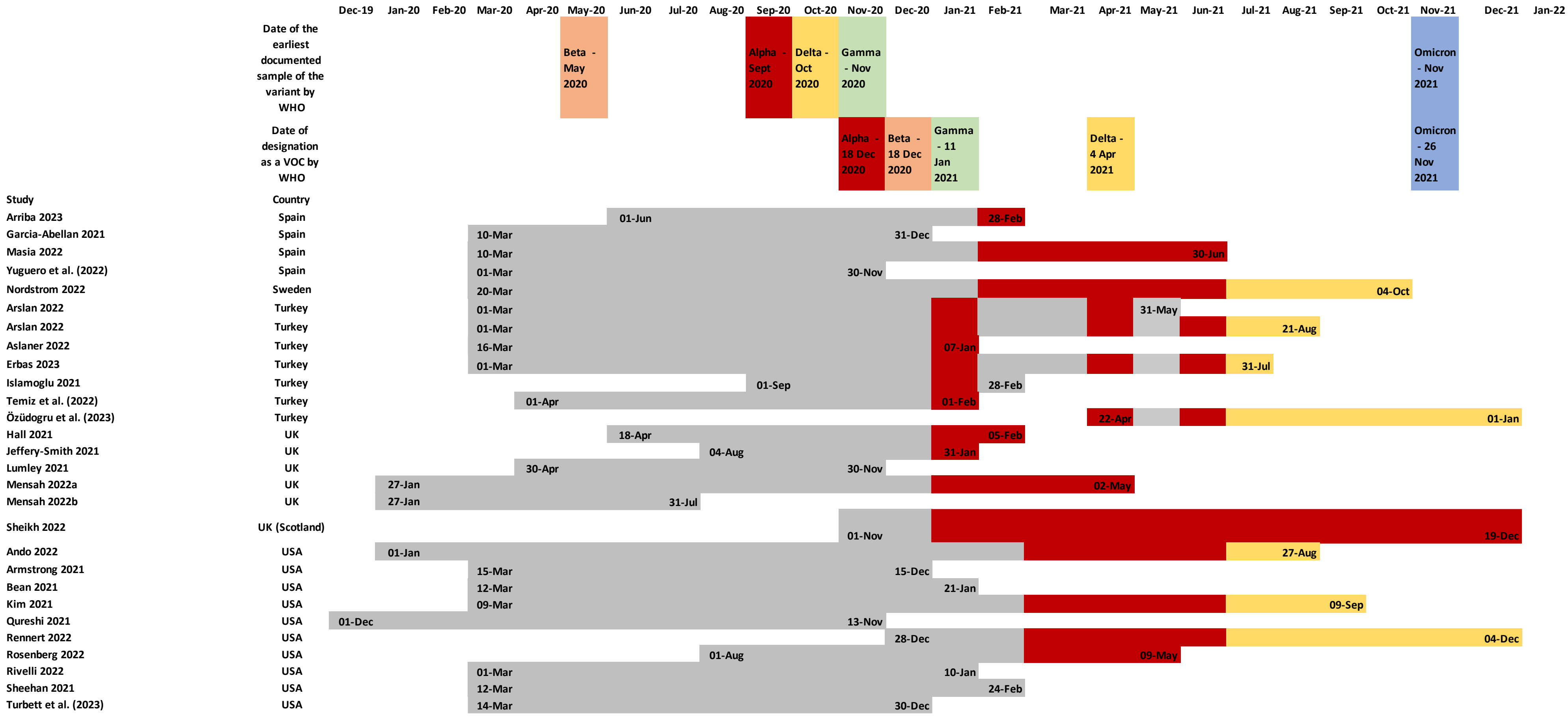

Figure S1: Mapping to predominant variant of SARS CoV-2 during the time of data collection of each study.

VOC: Variant of Concern, WHO: World Health Organization

Alpha B.1.1.7 | **Beta B.1.351** | **Gamma P.1** | **Delta B.1.617.2** | **Omicron B.1.1.529**

Table S5: Mapping of age-specific and gender-specific reporting of incidence proportion in studies included in the meta-analysis for age and gender.

Study	Age disaggregation												Gender disaggregation
	C1	C2	C3	C4	C5	C6	C7	C8	C9	C10	C11	C12	
Al Haddad et al. (2022)	<=12	N/A	N/A	N/A	N/A	N/A	N/A	N/A	N/A	N/A	N/A	N/A	No
Arriba et al. (2023)	12-17	18-49	50-69	70+	N/A	N/A	N/A	N/A	N/A	N/A	N/A	N/A	Yes
Cohen et al. (2023)	18-34	35+	N/A	N/A	N/A	N/A	N/A	N/A	N/A	N/A	N/A	N/A	Yes
Erbas et al. (2023)	<18	N/A	N/A	N/A	N/A	N/A	N/A	N/A	N/A	N/A	N/A	N/A	No
Flacco et al. (2021)	<18	18-59	60+	N/A	N/A	N/A	N/A	N/A	N/A	N/A	N/A	N/A	No
Hall et al. (2021)	N/A	N/A	N/A	N/A	N/A	N/A	N/A	N/A	N/A	N/A	N/A	N/A	Yes
Hammerman et al. (2022)	16-64	>=65	N/A	N/A	N/A	N/A	N/A	N/A	N/A	N/A	N/A	N/A	No
Kim et al. (2021)	0-17	18-34	35-50	51-64	65+	N/A	N/A	N/A	N/A	N/A	N/A	N/A	No
Konka et al. (2021)	N/A	N/A	N/A	N/A	N/A	N/A	N/A	N/A	N/A	N/A	N/A	N/A	Yes
Malhotra et al. (2022)	<25	25-44	>=45	N/A	N/A	N/A	N/A	N/A	N/A	N/A	N/A	N/A	Yes
Mensah et al. (a) (2022)	<20	20-29	30-39	40-49	50-59	60-69	70-79	80+	N/A	N/A	N/A	N/A	Yes
Mensah et al. (b) (2022)	<5	5-11	12-16	N/A	N/A	N/A	N/A	N/A	N/A	N/A	N/A	N/A	Yes
Nordstrom et al. (2022)	<50	50-64	65-79	>=80	N/A	N/A	N/A	N/A	N/A	N/A	N/A	N/A	Yes
Pecoraro et al. (2022)	0-14	15-29	30-49	50-69	70+	N/A	N/A	N/A	N/A	N/A	N/A	N/A	Yes
Qureshi et al. (2021)	<35	35-49	50-65	>65	N/A	N/A	N/A	N/A	N/A	N/A	N/A	N/A	Yes
Rennert et al. (2022)	18-24	N/A	N/A	N/A	N/A	N/A	N/A	N/A	N/A	N/A	N/A	N/A	No
Rivelli et al. (2022)	18-24	25-34	35-44	45-54	55-64	65+	N/A	N/A	N/A	N/A	N/A	N/A	Yes
Rosenberg et al. (2022)	<30	30-40	>40	N/A	N/A	N/A	N/A	N/A	N/A	N/A	N/A	N/A	Yes
Silva et al. (2023)	N/A	N/A	N/A	N/A	N/A	N/A	N/A	N/A	N/A	N/A	N/A	N/A	Yes

Table S6a: Incidence proportions of SARS CoV-2 reinfection generated from random effects model by background characteristics by age.

Study	Country	Population Type	Sample Size	Incidence (%)	95% CI	Weight
**Child (<18 years)**						
Flacco et al. (2021)	Italy	General population	832	0,00	0 (0,0.44)	1,46
Kim et al. (2021)	USA	General population	3156	0,10	0.1 (0.02,0.28)	2,36
Erbas et al. (2023)	Turkey	General population	8840	0,12	0.12 (0.06,0.22)	2,74
Mensah et al. (a) (2022)	UK	Mixed	587910	0,15	0.15 (0.14,0.16)	3,01
Mensah et al. (b) (2022)	UK	General population	109080	0,18	0.18 (0.16,0.21)	2,99
Mensah et al. (b) (2022)	UK	General population	256292	0,24	0.24 (0.22,0.26)	3,00
Arriba et al. (2023)	Spain	General population	7500	0,32	0.32 (0.21,0.48)	2,70
Mensah et al. (b) (2022)	UK	General population	314802	0,49	0.49 (0.47,0.52)	3,00
Malhotra et al. (2022)	India	Health service providers	459	3,27	3.27 (1.84,5.33)	1,03
Pecoraro et al. (2022)	Italy	General population	4926	4,22	4.22 (3.68,4.82)	2,56
Al Haddad et al. (2020)	Kuwait	General population	421	7,13	7.13 (4.86,10.02)	0,98
**Overall (I^2=99.3%, p<0.001)**			**1294218**	**0,57**	**0.57 (0.39,0.79)**	
**Young adults (18-35 years)**						
Kim et al. (2021)	USA	General population	11614	0,05	0.05 (0.02,0.11)	2,80
Mensah et al. (a) (2022)	UK	Mixed	707423	0,31	0.31 (0.3,0.32)	3,01
Mensah et al. (a) (2022)	UK	Mixed	689352	0,31	0.31 (0.3,0.32)	3,01
Rosenberg et al. (2022)	Unites States	General population	10801	0,65	0.65 (0.51,0.82)	2,79
Rennert et al. (2021)	Unites States	General population	4544	2,24		2,52
Malhotra et al. (2022)	India	Health service providers	3273	2,54	2.54 (2.02,3.13)	2,37
Cohen et al. (2023)	Israel	General population	1004	2,59	2.59 (1.7,3.77)	1,61
Rivelli et al. (2022)	USA	Health service providers	1040	4,90	4.9 (3.67,6.4)	1,63
Rivelli et al. (2022)	USA	Health service providers	200	8,00	8 (4.64,12.67)	0,56
**Overall (I^2=98.6%, p<0.001)**			**1429251**	**1,01**	**1.01 (0.8,1.25)**	
**Old adults (36-65 years)**						
Kim et al. (2021)	USA	General population	11785	0,06	0.06 (0.02,0.12)	2,80
Kim et al. (2021)	USA	General population	11618	0,09	0.09 (0.04,0.16)	2,80
Nordstrom et al. (2022)	Sweden	General population	274315	0,20	0.2 (0.18,0.22)	3,00
Mensah et al. (a) (2022)	UK	Mixed	601279	0,34	0.34 (0.33,0.36)	3,01

Mensah et al. (a) (2022)	UK	Mixed	576145	0,40	0.4 (0.38,0.42)	3,01
Mensah et al. (a) (2022)	UK	Mixed	308304	0,41	0.41 (0.39,0.43)	3,00
Qureshi et al. (2021)	USA	General population	2761	0,87	0.87 (0.56,1.29)	2,28
Qureshi et al. (2021)	USA	General population	1593	0,94	0.94 (0.53,1.55)	1,94
Malhotra et al. (2022)	India	Health service providers	1221	2,13	2.13 (1.4,3.1)	1,75
Rivelli et al. (2022)	USA	Health service providers	306	4,58	4.58 (2.52,7.56)	0,78
Rivelli et al. (2022)	USA	Health service providers	417	6,71	6.71 (4.51,9.56)	0,97
Rivelli et al. (2022)	USA	Health service providers	634	7,41	7.41 (5.5,9.74)	1,26
**Overall (I^2=98.4%, p<0.001)**			**1790378**	**0,53**	**0.53 (0.41,0.65)**	
**Elderly (65+ years)**						
Rivelli et al. (2022)	USA	Health service providers	28	0,00	0 (0,12.34)	0,09
Kim et al. (2021)	USA	General population	12154	0,10	0.1 (0.05,0.17)	2,81
Nordstrom et al. (2022)	Sweden	General population	74090	0,14	0.14 (0.11,0.17)	2,98
Arriba et al. (2023)	Spain	General population	7091	0,14	0.14 (0.07,0.26)	2,68
Nordstrom et al. (2022)	Sweden	General population	27305	0,36	0.36 (0.29,0.44)	2,92
Qureshi et al. (2021)	USA	General population	3072	0,42	0.42 (0.23,0.72)	2,34
Flacco et al. (2021)	Italy	General population	1963	0,51	0.51 (0.24,0.93)	2,08
Mensah et al. (a) (2022)	UK	Mixed	174848	0,52	0.52 (0.49,0.55)	3,00
Hammerman et al. (2022)	Israel	Health service providers	14183	0,54	0.54 (0.43,0.68)	2,84
Mensah et al. (a) (2022)	UK	Mixed	220339	1,00	1 (0.96,1.04)	3,00
Pecoraro et al. (2022)	Italy	General population	4765	2,90	2.9 (2.44,3.41)	2,54
**Overall (I^2=99.2%, p<0.001)**			**539838**	**0,37**	**0.37 (0.15,0.66)**	

Table S6b: Incidence proportions of SARS CoV-2 reinfection generated from random effects model by background characteristics by gender.

Study	Country	Popuilation Type	Sample Size	Incidence (%)	95% CI	Weight
**Male**						
Nordstrom et al. (2022)	Sweden	General population	722657	0,16	0.16 (0.15,0.17)	5,08
Mensah et al. (a) (2022)	UK	Mixed	1785745	0,25	0.25 (0.24,0.26)	5,09
Arriba et al. (2023)	Spain	General population	49542	0,26	0.26 (0.22,0.31)	5,01
Mensah et al. (b) (2022)	UK	General population	343141	0,34	0.34 (0.32,0.36)	5,08
Rosenberg et al. (2022)	Unites States	General population	5673	0,62	0.62 (0.43,0.86)	4,49
Qureshi et al. (2021)	USA	General population	4285	0,65	0.65 (0.43,0.94)	4,32
Hall et al. (2021)	UK	Health service providers	1425	2,11	2.11 (1.42,2.99)	3,32
Silva et al. (2023)	Brazil	General population	677	2,51	2.51 (1.47,3.99)	2,40
Cohen et al. (2023)	Israel	General population	789	2,66	2.66 (1.66,4.04)	2,60
Pecoraro et al. (2022)	Italy	General population	17719	3,30	3.3 (3.04,3.58)	4,88
Rivelli et al. (2022)	USA	Health service providers	361	3,88	3.88 (2.14,6.42)	1,65
Konka et al. (2021)	Poland	General population	219	4,11	4.11 (1.9,7.66)	1,14
Malhotra et al. (2022)	India	Health service providers	2717	5,59	5.59 (4.76,6.53)	3,98
**Overall (I^2=99.5%, p<0.001)**			**2934950**	**1,19**	**1.19 (0.95,1.46)**	
**Female**						
Nordstrom et al. (2022)	Sweden	General population	755230	0,22	0.22 (0.21,0.23)	5,08
Arriba et al. (2023)	Spain	General population	61185	0,34	0.34 (0.3,0.39)	5,03
Mensah et al. (b) (2022)	UK	General population	341202	0,35	0.35 (0.33,0.37)	5,08
Mensah et al. (a) (2022)	UK	Mixed	2060245	0,45	0.45 (0.44,0.46)	5,09
Rosenberg et al. (2022)	Unites States	General population	6601	0,59	0.59 (0.42,0.81)	4,56
Qureshi et al. (2021)	USA	General population	4789	0,73	0.73 (0.51,1.02)	4,39
Hall et al. (2021)	UK	Health service providers	6840	1,83	1.83 (1.52,2.17)	4,58
Konka et al. (2021)	Poland	General population	203	1,97	1.97 (0.54,4.97)	1,08
Silva et al. (2023)	Brazil	General population	696	2,16	2.16 (1.21,3.53)	2,44
Cohen et al. (2023)	Israel	General population	216	2,78	2.78 (1.03,5.95)	1,13
Pecoraro et al. (2022)	Italy	General population	17975	3,74	3.74 (3.47,4.03)	4,88
Rivelli et al. (2022)	USA	Health service providers	2264	6,27	6.27 (5.31,7.35)	3,81
Malhotra et al. (2022)	India	Health service providers	2236	6,71	6.71 (5.71,7.83)	3,80
**Overall (I^2=99.6%, p<0.001)**			**3259682**	**1,45**	**1.45 (1.16,1.78)**	

Table S6c: Incidence proportions of SARS CoV-2 reinfection generated from random effects model by background characteristics by population type.

Study	Country	Sample Size	Incidence (%)	95% CI	Weight
**General Population**					
Aslaner et al. (2022)	Turkey	104281	0.07	0.07 (0.06,0.09)	1.90
Temiz et al. (2022)	Turkey	14511	0.08	0.08 (0.04,0.14)	1.87
Kim et al. (2021)	USA	50327	0.08	0.08 (0.06,0.11)	1.89
Arslan et al. (2021)	Turkey	32607	0.08	0.08 (0.05,0.12)	1.89
Erbas et al. (2023)	Turkey	8840	0.12	0.12 (0.06,0.22)	1.85
Goldberg et al. (2022)	Israel	498954	0.18	0.18 (0.17,0.19)	1.90
Nordstrom et al. (2022)	Sweden	1477887	0.19	0.19 (0.18,0.2)	1.90
Akpan et al. (2022)	Liberia	5459	0.20	0.2 (0.1,0.36)	1.82
Gazit et al. (2022)	Israel	46035	0.23	0.23 (0.19,0.28)	1.89
Pilz et al. (2021)	Austria	14840	0.27	0.27 (0.19,0.37)	1.87
Freire-Neto et al. (2022)	Brazil	58097	0.29	0.29 (0.25,0.33)	1.90
Arriba et al. (2023)	Spain	110726	0.31	0.31 (0.28,0.34)	1.90
Salehi-Vaziri et al. (2021)	Iran	1492	0.34	0.34 (0.11,0.78)	1.64
Flacco et al. (2021)	Italy	7173	0.33	0.33 (0.21,0.5)	1.84
Shaheen et al. (2022)	Saudi Arabia	35288	0.37	0.37 (0.31,0.44)	1.89
Nguyen et al. (2022)	France	55338	0.40	0.4 (0.35,0.46)	1.90
Nielsen et al. (2022)	Denmark	502792	0.42	0.42 (0.4,0.44)	1.90
Abu-Raddad et al. (2022)	Qatar	364147	0.54	0.54 (0.52,0.56)	1.90
Berec et al. (2022)	Czech	1999315	0.54	0.54 (0.53,0.55)	1.90
Rosenberg et al. (2022)	Unites States	12272	0.60	0.6 (0.47,0.76)	1.87
Hønge et al. (2022)	Denmark	3806	0.60	0.6 (0.38,0.91)	1.79
Hansen et al. (2021)	Denmark	11068	0.65	0.65 (0.51,0.82)	1.86
Mensah et al. (b) (2022)	UK	688418	0.68	0.68 (0.66,0.7)	1.90
Arslan et al. (2022)	Turkey	58811	0.70	0.7 (0.63,0.77)	1.90
Islamoglu et al. (2021)	Turkey	1665	0.78	0.78 (0.42,1.33)	1.67
Al-Otaiby et al. (2022)	Saudi Arabia	556750	0.80	0.8 (0.78,0.82)	1.90
Michlmayr et al. (2022)	Denmark	245084	0.81	0.81 (0.77,0.84)	1.90
Tavakoli et al. (2023)	Iran	213768	0.83	0.83 (0.79,0.87)	1.90
Özüdogru et al. (2023)	Turkey	23495	1.00	1 (0.88,1.14)	1.88
Peghin et al. (2021)	Italy	546	1.10	1.1 (0.4,2.38)	1.33

Yuguero et al. (2022)	Spain	972	1.44	1.44 (0.79,2.4)	1.53
Abu-Raddad et al. (2021)	Qatar	15808	1.54	1.54 (1.35,1.74)	1.88
Hammerman et al. (2022)	Israel	149032	1.69	1.69 (1.63,1.76)	1.90
Sheikh et al. (2022)	UK	150351	1.80	1.8 (1.73,1.87)	1.90
Qureshi et al. (2021)	USA	9119	2.20	2.2 (1.91,2.53)	1.86
Rennert et al. (2022)	Unites States	4544	2.24	2.24 (1.83,2.72)	1.81
Silva et al. (2023)	Brazil	1371	2.33	2.33 (1.6,3.28)	1.62
Cohen et al. (2023)	Israel	1004	2.69	2.69 (1.78,3.89)	1.54
Konka et al. (2021)	Poland	422	3.08	3.08 (1.65,5.21)	1.22
Cocchio et al. (2022)	Italy	5552	3.24	3.24 (2.79,3.74)	1.83
Pecoraro et al. (2022)	Italy	35692	3.52	3.52 (3.34,3.72)	1.89
Ando et al. (2022)	Unites States	165320	3.70	3.7 (3.61,3.79)	1.90
Turbett et al. (2023)	Unites States	1569	4.14	4.14 (3.21,5.25)	1.65
Bean et al. (2021)	USA	1669	4.49	4.49 (3.55,5.6)	1.67
Sheehan et al. (2021)	USA	1278	4.93	4.93 (3.81,6.26)	1.61
Al Haddad et al. (2022)	Kuwait	421	7.13	7.13 (4.86,10.02)	1.22
García-Abellán et al. (2021)	Spain	146	11.64	11.64 (6.93,17.99)	0.73
**Overall (I^2=99.8%, p<0.001)**		**7748062**	**1.00**	1 (0.81,1.2)	
**Health service providers**					
Gallais et al. (2021)	France	345	0.29	0.29 (0.01,1.6)	1.13
Lumley et al. (2021)	UK	466	0.43	0.43 (0.05,1.54)	1.26
Guedes et al. (2023)	Brazil	4199	0.79	0.79 (0.54,1.1)	1.80
Nisha et al. (2022)	India	350	1.14	1.14 (0.31,2.9)	1.14
Dhumal et al. (2022)	India	408	1.47	1.47 (0.54,3.17)	1.21
Hall et al. (2021)	UK	8278	1.87	1.87 (1.59,2.19)	1.85
Ronchini et al. (2022)	Italy	266	1.88	1.88 (0.61,4.33)	1.01
Malhotra et al. (2022)	India	4953	2.50	2.5 (2.09,2.98)	1.82
Reddy et al. (2023)	India	1150	4.87	4.87 (3.7,6.28)	1.58
Rahman et al. (2022)	Bangladesh	731	5.20	5.2 (3.7,7.07)	1.44
Comelli et al. (2021)	Italy	160	5.63	5.63 (2.6,10.41)	0.77
Rivelli et al. (2022)	USA	2625	5.94	5.94 (5.07,6.92)	1.75
**Overall (I^2=99.4%, p<0.001)**		**23931**	**2.28**	2.28 (1.37,3.4)	

Table S6d: Incidence proportions of SARS CoV-2 reinfection generated from random effects model for studies containing only unvaccinated samples.

Study	Country	Population Type	Sample Size	Incidence (%)	95% CI	Weight
Aslaner et al. (2022)	Turkey	General population	104281	0,07	0.07 (0.06,0.09)	7,31
Erbas et al. (2023)	Turkey	General population	8840	0,12	0.12 (0.06,0.22)	6,99
Goldberg et al. (2022)	Israel	General population	498954	0,18	0.18 (0.17,0.19)	7,33
Nordstrom et al. (2022)	Sweden	General population	1477887	0,19	0.19 (0.18,0.2)	7,34
Gazit et al. (2022)	Israel	General population	46035	0,23	0.23 (0.19,0.28)	7,27
Abu-Raddad et al. (2022)	Qatar	General population	364147	0,54	0.54 (0.52,0.56)	7,33
Rosenberg et al. (2022)	Unites States	General population	12272	0,60	0.6 (0.47,0.76)	7,08
Hønge et al. (2022)	Denmark	General population	3806	0,60	0.6 (0.38,0.91)	6,57
Mensah et al. (b) (2022)	UK	General population	688418	0,68	0.68 (0.66,0.7)	7,34
Michlmayr et al. (2022)	Denmark	General population	245084	0,81	0.81 (0.77,0.84)	7,33
Yuguero et al. (2022)	Spain	General population	972	1,44	1.44 (0.79,2.4)	5,04
Ronchini et al. (2022)	Italy	Health service providers	266	1,88	1.88 (0.61,4.33)	2,76
Armstrong et al. (2021)	USA	Special risk group	6079	2,60	2.6 (2.21,3.03)	6,84
Turbett et al. (2023)	Unites States	General population	1569	4,14	4.14 (3.21,5.25)	5,72
Bean et al. (2021)	USA	General population	1669	4,49	4.49 (3.55,5.6)	5,80
Comelli et al. (2021)	Italy	Health service providers	160	5,63	5.63 (2.6,10.41)	1,95
**Overall (I^2=99.7%, p<0.001, Tau^2=0.002)**				**0,83**	**0.83 (0.61,1.08)**	

Table S6e: Incidence proportions of SARS CoV-2 reinfection generated from random effects model by background characteristics by regions.

Study	Country	Population Type	Sample Size	Incidence (%)	95% CI	Weight
**Asia**						
Aslaner et al. (2022)	Turkey	General population	104281	0.07	0.07 (0.06,0.09)	1.86
Temiz et al. (2022)	Turkey	General population	14511	0.08	0.08 (0.04,0.14)	1.82
Arslan et al. (2021)	Turkey	General population	32607	0.08	0.08 (0.05,0.12)	1.85
Erbas et al. (2023)	Turkey	General population	8840	0.12	0.12 (0.06,0.22)	1.80
Goldberg et al. (2022)	Israel	General population	498954	0.18	0.18 (0.17,0.19)	1.86
Gazit et al. (2022)	Israel	General population	46035	0.23	0.23 (0.19,0.28)	1.85
Salehi-Vaziri et al. (2021)	Iran	General population	1492	0.34	0.34 (0.11,0.78)	1.53
Shaheen et al. (2022)	Saudi Arabia	General population	35288	0.37	0.37 (0.31,0.44)	1.85
Abu-Raddad et al. (2022)	Qatar	General population	364147	0.54	0.54 (0.52,0.56)	1.86
Arslan et al. (2022)	Turkey	General population	58811	0.70	0.7 (0.63,0.77)	1.85
Islamoglu et al. (2021)	Turkey	General population	1665	0.78	0.78 (0.42,1.33)	1.56
Al-Otaiby et al. (2022)	Saudi Arabia	General population	556750	0.80	0.8 (0.78,0.82)	1.86
Tavakoli et al. (2023)	Iran	General population	213768	0.83	0.83 (0.79,0.87)	1.86
Özüdogru et al. (2023)	Turkey	General population	23495	1.00	1 (0.88,1.14)	1.84
Nisha et al. (2022)	India	Health service providers	350	1.14	1.14 (0.31,2.9)	0.97
Dhumal et al. (2022)	India	Health service providers	408	1.47	1.47 (0.54,3.17)	1.05
Abu-Raddad et al. (2021)	Qatar	General population	15808	1.54	1.54 (1.35,1.74)	1.83
Hammerman et al. (2022)	Israel	General population	149032	1.69	1.69 (1.63,1.76)	1.86
Malhotra et al. (2022)	India	Health service providers	4953	2.50	2.5 (2.09,2.98)	1.75
Cohen et al. (2023)	Israel	General population	1004	2.69	2.69 (1.78,3.89)	1.41
Reddy et al. (2023)	India	Health service providers	1150	4.87	4.87 (3.7,6.28)	1.46
Rahman et al. (2022)	Bangladesh	Health service providers	731	5.20	5.2 (3.7,7.07)	1.30
Al Haddad et al. (2022)	Kuwait	General population	421	7.13	7.13 (4.86,10.02)	1.06
**Overall (I^2=99.7%, p<0.001)**			**2134501**	**0.94**	**0.94 (0.69,1.21)**	
**Europe**						
Nordstrom et al. (2022)	Sweden	General population	1477887	0.19	0.19 (0.18,0.2)	1.86
Pilz et al. (2021)	Austria	General population	14840	0.27	0.27 (0.19,0.37)	1.82
Gallais et al. (2021)	France	Health service providers	345	0.29	0.29 (0.01,1.6)	0.97
Arriba et al. (2023)	Spain	General population	110726	0.31	0.31 (0.28,0.34)	1.86
Flacco et al. (2021)	Italy	General population	7173	0.33	0.33 (0.21,0.5)	1.78
Mensah et al. (a) (2022)	UK	Mixed	3860054	0.37	0.37 (0.36,0.38)	1.86

Nguyen et al. (2022)	France	General population	55338	0.40	0.4 (0.35,0.46)	1.85
Nielsen et al. (2022)	Denmark	General population	502792	0.42	0.42 (0.4,0.44)	1.86
Lumley et al. (2021)	UK	Health service providers	466	0.43	0.43 (0.05,1.54)	1.11
Berec et al. (2022)	Czech	General population	1999315	0.54	0.54 (0.53,0.55)	1.86
Hønge et al. (2022)	Denmark	General population	3806	0.60	0.6 (0.38,0.91)	1.72
Hansen et al. (2021)	Denmark	General population	11068	0.65	0.65 (0.51,0.82)	1.81
Mensah et al. (b) (2022)	UK	General population	688418	0.68	0.68 (0.66,0.7)	1.86
Michlmayr et al. (2022)	Denmark	General population	245084	0.81	0.81 (0.77,0.84)	1.86
Peghin et al. (2021)	Italy	General population	546	1.10	1.1 (0.4,2.38)	1.18
Attauabi et al. (2021)	Denmark	Special risk group	315	1.27	1.27 (0.35,3.22)	0.93
Yuguero et al. (2022)	Spain	General population	972	1.44	1.44 (0.79,2.4)	1.40
Jeffery-Smith et al. (2021)	UK	Special risk group	656	1.52	1.52 (0.73,2.79)	1.25
Sheikh et al. (2022)	UK	General population	150351	1.80	1.8 (1.73,1.87)	1.86
Hall et al. (2021)	UK	Health service providers	8278	1.87	1.87 (1.59,2.19)	1.79
Ronchini et al. (2022)	Italy	Health service providers	266	1.88	1.88 (0.61,4.33)	0.85
Konka et al. (2021)	Poland	General population	422	3.08	3.08 (1.65,5.21)	1.06
Cocchio et al. (2022)	Italy	General population	5552	3.24	3.24 (2.79,3.74)	1.76
Pecoraro et al. (2022)	Italy	General population	35692	3.52	3.52 (3.34,3.72)	1.85
Masia et al. (2022)	Spain	Special risk group	150	5.33	5.33 (2.33,10.24)	0.60
Comelli et al. (2021)	Italy	Health service providers	160	5.63	5.63 (2.6,10.41)	0.62
García-Abellán et al. (2021)	Spain	General population	146	11.64	11.64 (6.93,17.99)	0.59
**Overall (I^2=99.8%, p<0.001)**			**9180818**	**0.93**	**0.93 (0.77,1.12)**	
**America**						
Kim et al. (2021)	USA	General population	50327	0.08	0.08 (0.06,0.11)	1.85
Freire-Neto et al. (2022)	Brazil	General population	58097	0.29	0.29 (0.25,0.33)	1.85
Rosenberg et al. (2022)	USA	General population	12272	0.60	0.6 (0.47,0.76)	1.82
Guedes et al. (2023)	Brazil	Health service providers	4199	0.79	0.79 (0.54,1.1)	1.73
Qureshi et al. (2021)	USA	General population	9119	2.20	2.2 (1.91,2.53)	1.80
Rennert et al. (2022)	USA	General population	4544	2.24	2.24 (1.83,2.72)	1.74
Silva et al. (2023)	Brazil	General population	1371	2.33	2.33 (1.6,3.28)	1.51
Armstrong et al. (2021)	USA	Special risk group	6079	2.60	2.6 (2.21,3.03)	1.77
Ando et al. (2022)	USA	General population	165320	3.70	3.7 (3.61,3.79)	1.86
Turbett et al. (2023)	USA	General population	1569	4.14	4.14 (3.21,5.25)	1.55
Bean et al. (2021)	USA	General population	1669	4.49	4.49 (3.55,5.6)	1.56
Sheehan et al. (2021)	USA	General population	1278	4.93	4.93 (3.81,6.26)	1.49

Rivelli et al. (2022)	USA	Health service providers	2625	5.94	5.94 (5.07,6.92)	1.66
**Overall (I^2=99.8%, p<0.001)**			**318469**	**2.21**	**2.21 (1.02,3.84)**	

Study	Population Type	Sample Size	Incidence (%)	95% CI	Weight
**North America**					
Kim et al. (2021)	General population	50327	0.08	0.08(0.06,0.11)	7.76
Rosenberg et al. (2022)	General population	12272	0.60	0.6(0.47,0.76)	7.75
Qureshi et al. (2021)	General population	9119	2.20	2.2(1.91,2.53)	7.74
Rennert et al. (2022)	General population	4544	2.24	2.24(1.83,2.72)	7.71
Armstrong et al. (2021)	Special risk group	6079	2.60	2.6(2.21,3.03)	7.73
Ando et al. (2022)	General population	165320	3.70	3.7(3.61,3.79)	7.77
Turbett et al. (2023)	General population	1569	4.14	4.14(3.21,5.25)	7.61
Bean et al. (2021)	General population	1669	4.49	4.49(3.55,5.6)	7.62
Sheehan et al. (2021)	General population	1278	4.93	4.93(3.81,6.26)	7.58
Rivelli et al. (2022)	Health service providers	2625	5.94	5.94(5.07,6.92)	7.67
**Overall (I^2=99.8%, p<0.001)**			2.68	2.68(1.14,4.85)	
**South America**					
Freire-Neto et al. (2022)	General population	58097	0.29	0.29(0.25,0.33)	7.76
Guedes et al. (2023)	Health service providers	4199	0.79	0.79(0.54,1.1)	7.71
Silva et al. (2023)	General population	1371	2.33	2.33(1.6,3.28)	7.59
**Overall (I^2=. %, p= .)**			0.93	0.93(0.23,2.07)	

Table S6f: Incidence proportions of SARS CoV-2 reinfection generated from random effects model by background characteristics by World Bank country income group.

Study	Country	Population Type	Sample Size	Incidence (%)	95% CI	Weight
**High Income Country**						
Kim et al. (2021)	USA	General population	50327	0.08	0.08 (0.06,0.11)	1.82
Goldberg et al. (2022)	Israel	General population	498954	0.18	0.18 (0.17,0.19)	1.83
Nordstrom et al. (2022)	Sweden	General population	1477887	0.19	0.19 (0.18,0.2)	1.83
Gazit et al. (2022)	Israel	General population	46035	0.23	0.23 (0.19,0.28)	1.82
Pilz et al. (2021)	Austria	General population	14840	0.27	0.27 (0.19,0.37)	1.79
Gallais et al. (2021)	France	Health service providers	345	0.29	0.29 (0.01,1.6)	0.95
Arriba et al. (2023)	Spain	General population	110726	0.31	0.31 (0.28,0.34)	1.83
Flacco et al. (2021)	Italy	General population	7173	0.33	0.33 (0.21,0.5)	1.75
Mensah et al. (a) (2022)	UK	Mixed	3860054	0.37	0.37 (0.36,0.38)	1.83
Shaheen et al. (2022)	Saudi Arabia	General population	35288	0.37	0.37 (0.31,0.44)	1.82
Nguyen et al. (2022)	France	General population	55338	0.40	0.4 (0.35,0.46)	1.82
Nielsen et al. (2022)	Denmark	General population	502792	0.42	0.42 (0.4,0.44)	1.83
Lumley et al. (2021)	UK	Health service providers	466	0.43	0.43 (0.05,1.54)	1.09
Abu-Raddad et al. (2022)	Qatar	General population	364147	0.54	0.54 (0.52,0.56)	1.83
Berec et al. (2022)	Czech	General population	1999315	0.54	0.54 (0.53,0.55)	1.83
Rosenberg et al. (2022)	Unites States	General population	12272	0.60	0.6 (0.47,0.76)	1.79
Hønge et al. (2022)	Denmark	General population	3806	0.60	0.6 (0.38,0.91)	1.69
Hansen et al. (2021)	Denmark	General population	11068	0.65	0.65 (0.51,0.82)	1.78
Mensah et al. (b) (2022)	UK	General population	688418	0.68	0.68 (0.66,0.7)	1.83
Al-Otaiby et al. (2022)	Saudi Arabia	General population	556750	0.80	0.8 (0.78,0.82)	1.83
Michlmayr et al. (2022)	Denmark	General population	245084	0.81	0.81 (0.77,0.84)	1.83
Peghin et al. (2021)	Italy	General population	546	1.10	1.1 (0.4,2.38)	1.16
Attauabi et al. (2021)	Denmark	Special risk group	315	1.27	1.27 (0.35,3.22)	0.91
Yuguero et al. (2022)	Spain	General population	972	1.44	1.44 (0.79,2.4)	1.38
Jeffery-Smith et al. (2021)	UK	Special risk group	656	1.52	1.52 (0.73,2.79)	1.23
Abu-Raddad et al. (2021)	Qatar	General population	15808	1.54	1.54 (1.35,1.74)	1.80
Hammerman et al. (2022)	Israel	General population	149032	1.69	1.69 (1.63,1.76)	1.83
Sheikh et al. (2022)	UK	General population	150351	1.80	1.8 (1.73,1.87)	1.83
Hall et al. (2021)	UK	Health service providers	8278	1.87	1.87 (1.59,2.19)	1.76
Ronchini et al. (2022)	Italy	Health service providers	266	1.88	1.88 (0.61,4.33)	0.83
Qureshi et al. (2021)	USA	General population	9119	2.20	2.2 (1.91,2.53)	1.77
Rennert et al. (2022)	Unites States	General population	4544	2.24	2.24 (1.83,2.72)	1.71

Armstrong et al. (2021)	USA	Special risk group	6079	2.60	2.6 (2.21,3.03)	1.74
Cohen et al. (2023)	Israel	General population	1004	2.69	2.69 (1.78,3.89)	1.39
Konka et al. (2021)	Poland	General population	422	3.08	3.08 (1.65,5.21)	1.04
Cocchio et al. (2022)	Italy	General population	5552	3.24	3.24 (2.79,3.74)	1.73
Pecoraro et al. (2022)	Italy	General population	35692	3.52	3.52 (3.34,3.72)	1.82
Ando et al. (2022)	Unites States	General population	165320	3.70	3.7 (3.61,3.79)	1.83
Turbett et al. (2023)	Unites States	General population	1569	4.14	4.14 (3.21,5.25)	1.52
Bean et al. (2021)	USA	General population	1669	4.49	4.49 (3.55,5.6)	1.54
Sheehan et al. (2021)	USA	General population	1278	4.93	4.93 (3.81,6.26)	1.47
Masia et al. (2022)	Spain	Special risk group	150	5.33	5.33 (2.33,10.24)	0.59
Comelli et al. (2021)	Italy	Health service providers	160	5.63	5.63 (2.6,10.41)	0.61
Rivelli et al. (2022)	USA	Health service providers	2625	5.94	5.94 (5.07,6.92)	1.63
Al Haddad et al. (2022)	Kuwait	General population	421	7.13	7.13 (4.86,10.02)	1.04
García-Abellán et al. (2021)	Spain	General population	146	11.64	11.64 (6.93,17.99)	0.57
**Overall (I^2=99.8%, p<0.001)**			**11103059**	**1.33**	**1.33 (1.13,1.54)**	
**Non High Income Country**						
Aslaner et al. (2022)	Turkey	General population	104281	0.07	0.07 (0.06,0.09)	1.83
Temiz et al. (2022)	Turkey	General population	14511	0.08	0.08 (0.04,0.14)	1.79
Arslan et al. (2021)	Turkey	General population	32607	0.08	0.08 (0.05,0.12)	1.81
Erbas et al. (2023)	Turkey	General population	8840	0.12	0.12 (0.06,0.22)	1.77
Akpan et al. (2022)	Liberia	General population	5459	0.20	0.2 (0.1,0.36)	1.73
Freire-Neto et al. (2022)	Brazil	General population	58097	0.29	0.29 (0.25,0.33)	1.82
Salehi-Vaziri et al. (2021)	Iran	General population	1492	0.34	0.34 (0.11,0.78)	1.51
Arslan et al. (2022)	Turkey	General population	58811	0.70	0.7 (0.63,0.77)	1.82
Islamoglu et al. (2021)	Turkey	General population	1665	0.78	0.78 (0.42,1.33)	1.54
Guedes et al. (2023)	Brazil	Health service providers	4199	0.79	0.79 (0.54,1.1)	1.70
Tavakoli et al. (2023)	Iran	General population	213768	0.83	0.83 (0.79,0.87)	1.83
Özüdogru et al. (2023)	Turkey	General population	23495	1.00	1 (0.88,1.14)	1.81
Nisha et al. (2022)	India	Health service providers	350	1.14	1.14 (0.31,2.9)	0.96
Dhumal et al. (2022)	India	Health service providers	408	1.47	1.47 (0.54,3.17)	1.03
Silva et al. (2023)	Brazil	General population	1371	2.33	2.33 (1.6,3.28)	1.49
Malhotra et al. (2022)	India	Health service providers	4953	2.50	2.5 (2.09,2.98)	1.72
Reddy et al. (2023)	India	Health service providers	1150	4.87	4.87 (3.7,6.28)	1.43
Rahman et al. (2022)	Bangladesh	Health service providers	731	5.20	5.2 (3.7,7.07)	1.27
**Overall (I^2=99.2%, p<0.001)**			**536188**	**0.83**	**0.83 (0.55,1.18)**	

Table S6g: Incidence proportions of SARS CoV-2 reinfection generated from random effects model by background characteristics by study mid-year.

Study/ Mid-year	Country	Population Type	Sample Size	Incidence (%)	95% CI	Weight
**2020**						
Aslaner et al. (2022)	Turkey	General population	104281	0.07	0.07 (0.06,0.09)	1.83
Temiz et al. (2022)	Turkey	General population	14511	0.08	0.08 (0.04,0.14)	1.79
Kim et al. (2021)	USA	General population	50327	0.08	0.08 (0.06,0.11)	1.82
Arslan et al. (2021)	Turkey	General population	32607	0.08	0.08 (0.05,0.12)	1.81
Erbas et al. (2023)	Turkey	General population	8840	0.12	0.12 (0.06,0.22)	1.77
Goldberg et al. (2022)	Israel	General population	498954	0.18	0.18 (0.17,0.19)	1.83
Nordstrom et al. (2022)	Sweden	General population	1477887	0.19	0.19 (0.18,0.2)	1.83
Akpan et al. (2022)	Liberia	General population	5459	0.20	0.2 (0.1,0.36)	1.73
Gazit et al. (2022)	Israel	General population	46035	0.23	0.23 (0.19,0.28)	1.82
Pilz et al. (2021)	Austria	General population	14840	0.27	0.27 (0.19,0.37)	1.79
Gallais et al. (2021)	France	Health service providers	345	0.29	0.29 (0.01,1.6)	0.95
Arriba et al. (2023)	Spain	General population	110726	0.31	0.31 (0.28,0.34)	1.83
Flacco et al. (2021)	Italy	General population	7173	0.33	0.33 (0.21,0.5)	1.75
Shaheen et al. (2022)	Saudi Arabia	General population	35288	0.37	0.37 (0.31,0.44)	1.82
Mensah et al. (a) (2022)	UK	Mixed	3860054	0.37	0.37 (0.36,0.38)	1.83
Nguyen et al. (2022)	France	General population	55338	0.40	0.4 (0.35,0.46)	1.82
Nielsen et al. (2022)	Denmark	General population	502792	0.42	0.42 (0.4,0.44)	1.83
Lumley et al. (2021)	UK	Health service providers	466	0.43	0.43 (0.05,1.54)	1.09
Abu-Raddad et al. (2022)	Qatar	General population	364147	0.54	0.54 (0.52,0.56)	1.83
Rosenberg et al. (2022)	Unites States	General population	12272	0.60	0.6 (0.47,0.76)	1.79
Hønge et al. (2022)	Denmark	General population	3806	0.60	0.6 (0.38,0.91)	1.69
Hansen et al. (2021)	Denmark	General population	11068	0.65	0.65 (0.51,0.82)	1.78
Mensah et al. (b) (2022)	UK	General population	688418	0.68	0.68 (0.66,0.7)	1.83
Arslan et al. (2022)	Turkey	General population	58811	0.70	0.7 (0.63,0.77)	1.82
Islamoglu et al. (2021)	Turkey	General population	1665	0.78	0.78 (0.42,1.33)	1.54
Michlmayr et al. (2022)	Denmark	General population	245084	0.81	0.81 (0.77,0.84)	1.83
Tavakoli et al. (2023)	Iran	General population	213768	0.83	0.83 (0.79,0.87)	1.83
Peghin et al. (2021)	Italy	General population	546	1.10	1.1 (0.4,2.38)	1.16
Nisha et al. (2022)	India	Health service providers	350	1.14	1.14 (0.31,2.9)	0.96
Attauabi et al. (2021)	Denmark	Special risk group	315	1.27	1.27 (0.35,3.22)	0.91
Yuguero et al. (2022)	Spain	General population	972	1.44	1.44 (0.79,2.4)	1.38
Dhumal et al. (2022)	India	Health service providers	408	1.47	1.47 (0.54,3.17)	1.03

Jeffery-Smith et al. (2021)	UK	Special risk group	656	1.52	1.52 (0.73,2.79)	1.23
Abu-Raddad et al. (2021)	Qatar	General population	15808	1.54	1.54 (1.35,1.74)	1.80
Hall et al. (2021)	UK	Health service providers	8278	1.87	1.87 (1.59,2.19)	1.76
Ronchini et al. (2022)	Italy	Health service providers	266	1.88	1.88 (0.61,4.33)	0.83
Qureshi et al. (2021)	USA	General population	9119	2.20	2.2 (1.91,2.53)	1.77
Silva et al. (2023)	Brazil	General population	1371	2.33	2.33 (1.6,3.28)	1.49
Malhotra et al. (2022)	India	Health service providers	4953	2.50	2.5 (2.09,2.98)	1.72
Armstrong et al. (2021)	USA	Special risk group	6079	2.60	2.6 (2.21,3.03)	1.74
Konka et al. (2021)	Poland	General population	422	3.08	3.08 (1.65,5.21)	1.04
Cocchio et al. (2022)	Italy	General population	5552	3.24	3.24 (2.79,3.74)	1.73
Pecoraro et al. (2022)	Italy	General population	35692	3.52	3.52 (3.34,3.72)	1.82
Ando et al. (2022)	Unites States	General population	165320	3.70	3.7 (3.61,3.79)	1.83
Turbett et al. (2023)	Unites States	General population	1569	4.14	4.14 (3.21,5.25)	1.52
Bean et al. (2021)	USA	General population	1669	4.49	4.49 (3.55,5.6)	1.54
Reddy et al. (2023)	India	Health service providers	1150	4.87	4.87 (3.7,6.28)	1.43
Sheehan et al. (2021)	USA	General population	1278	4.93	4.93 (3.81,6.26)	1.47
Rahman et al. (2022)	Bangladesh	Health service providers	731	5.20	5.2 (3.7,7.07)	1.27
Masia et al. (2022)	Spain	Special risk group	150	5.33	5.33 (2.33,10.24)	0.59
Comelli et al. (2021)	Italy	Health service providers	160	5.63	5.63 (2.6,10.41)	0.61
Rivelli et al. (2022)	USA	Health service providers	2625	5.94	5.94 (5.07,6.92)	1.63
Al Haddad et al. (2022)	Kuwait	General population	421	7.13	7.13 (4.86,10.02)	1.04
García-Abellán et al. (2021)	Spain	General population	146	11.64	11.64 (6.93,17.99)	0.57
**Overall (I^2=99.8%, p<0.001)**			**8690968**	**1.20**	**1.2 (1.02,1.4)**	
**2021**						
Freire-Neto et al. (2022)	Brazil	General population	58097	0.29	0.29 (0.25,0.33)	1.82
Salehi-Vaziri et al. (2021)	Iran	General population	1492	0.34	0.34 (0.11,0.78)	1.51
Berec et al. (2022)	Czech	General population	1999315	0.54	0.54 (0.53,0.55)	1.83
Guedes et al. (2023)	Brazil	Health service providers	4199	0.79	0.79 (0.54,1.1)	1.70
Al-Otaiby et al. (2022)	Saudi Arabia	General population	556750	0.80	0.8 (0.78,0.82)	1.83
Özüdogru et al. (2023)	Turkey	General population	23495	1.00	1 (0.88,1.14)	1.81
Hammerman et al. (2022)	Israel	General population	149032	1.69	1.69 (1.63,1.76)	1.83
Sheikh et al. (2022)	UK	General population	150351	1.80	1.8 (1.73,1.87)	1.83
Rennert et al. (2022)	Unites States	General population	4544	2.24	2.24 (1.83,2.72)	1.71
Cohen et al. (2023)	Israel	General population	1004	2.69	2.69 (1.78,3.89)	1.39
**Overall (I^2=99.8%, p<0.001)**			**2948279**	**1.06**	**1.06 (0.74,1.44)**	

Table S6h: Incidence proportions of SARS CoV-2 reinfection generated from random effects model by background characteristics by study design.

Study	Country	Population Type	Sample Size	Incidence (%)	95% CI	Weight
**Prospective**						
Gallais et al. (2021)	France	Health service providers	345	0.29	0.29 (0.01,1.6)	0.95
Salehi-Vaziri et al. (2021)	Iran	General population	1492	0.34	0.34 (0.11,0.78)	1.51
Lumley et al. (2021)	UK	Health service providers	466	0.43	0.43 (0.05,1.54)	1.09
Mensah et al. (b) (2022)	UK	General population	688418	0.68	0.68 (0.66,0.7)	1.83
Islamoglu et al. (2021)	Turkey	General population	1665	0.78	0.78 (0.42,1.33)	1.54
Guedes et al. (2023)	Brazil	Health service providers	4199	0.79	0.79 (0.54,1.1)	1.70
Peghin et al. (2021)	Italy	General population	546	1.10	1.1 (0.4,2.38)	1.16
Attauabi et al. (2021)	Denmark	Special risk group	315	1.27	1.27 (0.35,3.22)	0.91
Jeffery-Smith et al. (2021)	UK	Special risk group	656	1.52	1.52 (0.73,2.79)	1.23
Sheikh et al. (2022)	UK	General population	150351	1.80	1.8 (1.73,1.87)	1.83
Hall et al. (2021)	UK	Health service providers	8278	1.87	1.87 (1.59,2.19)	1.76
Ronchini et al. (2022)	Italy	Health service providers	266	1.88	1.88 (0.61,4.33)	0.83
Silva et al. (2023)	Brazil	General population	1371	2.33	2.33 (1.6,3.28)	1.49
Cohen et al. (2023)	Israel	General population	1004	2.69	2.69 (1.78,3.89)	1.39
Reddy et al. (2023)	India	Health service providers	1150	4.87	4.87 (3.7,6.28)	1.43
Rahman et al. (2022)	Bangladesh	Health service providers	731	5.20	5.2 (3.7,7.07)	1.27
Masia et al. (2022)	Spain	Special risk group	150	5.33	5.33 (2.33,10.24)	0.59
Rivelli et al. (2022)	USA	Health service providers	2625	5.94	5.94 (5.07,6.92)	1.63
García-Abellán et al. (2021)	Spain	General population	146	11.64	11.64 (6.93,17.99)	0.57
**Overall (I^2=99%, p<0.001)**			**864174**	**1.96**	**1.96 (1.43,2.57)**	
**Retrospective**						
Aslaner et al. (2022)	Turkey	General population	104281	0.07	0.07 (0.06,0.09)	1.83
Temiz et al. (2022)	Turkey	General population	14511	0.08	0.08 (0.04,0.14)	1.79
Kim et al. (2021)	USA	General population	50327	0.08	0.08 (0.06,0.11)	1.82
Arslan et al. (2021)	Turkey	General population	32607	0.08	0.08 (0.05,0.12)	1.81
Erbas et al. (2023)	Turkey	General population	8840	0.12	0.12 (0.06,0.22)	1.77
Goldberg et al. (2022)	Israel	General population	498954	0.18	0.18 (0.17,0.19)	1.83
Nordstrom et al. (2022)	Sweden	General population	1477887	0.19	0.19 (0.18,0.2)	1.83
Akpan et al. (2022)	Liberia	General population	5459	0.20	0.2 (0.1,0.36)	1.73
Gazit et al. (2022)	Israel	General population	46035	0.23	0.23 (0.19,0.28)	1.82
Pilz et al. (2021)	Austria	General population	14840	0.27	0.27 (0.19,0.37)	1.79

Freire-Neto et al. (2022)	Brazil	General population	58097	0.29	0.29 (0.25,0.33)	1.82
Arriba et al. (2023)	Spain	General population	110726	0.31	0.31 (0.28,0.34)	1.83
Flacco et al. (2021)	Italy	General population	7173	0.33	0.33 (0.21,0.5)	1.75
Mensah et al. (a) (2022)	UK	Mixed	3860054	0.37	0.37 (0.36,0.38)	1.83
Shaheen et al. (2022)	Saudi Arabia	General population	35288	0.37	0.37 (0.31,0.44)	1.82
Nguyen et al. (2022)	France	General population	55338	0.40	0.4 (0.35,0.46)	1.82
Nielsen et al. (2022)	Denmark	General population	502792	0.42	0.42 (0.4,0.44)	1.83
Abu-Raddad et al. (2022)	Qatar	General population	364147	0.54	0.54 (0.52,0.56)	1.83
Berec et al. (2022)	Czech	General population	1999315	0.54	0.54 (0.53,0.55)	1.83
Rosenberg et al. (2022)	Unites States	General population	12272	0.60	0.6 (0.47,0.76)	1.79
Hønge et al. (2022)	Denmark	General population	3806	0.60	0.6 (0.38,0.91)	1.69
Hansen et al. (2021)	Denmark	General population	11068	0.65	0.65 (0.51,0.82)	1.78
Arslan et al. (2022)	Turkey	General population	58811	0.70	0.7 (0.63,0.77)	1.82
Al-Otaiby et al. (2022)	Saudi Arabia	General population	556750	0.80	0.8 (0.78,0.82)	1.83
Michlmayr et al. (2022)	Denmark	General population	245084	0.81	0.81 (0.77,0.84)	1.83
Tavakoli et al. (2023)	Iran	General population	213768	0.83	0.83 (0.79,0.87)	1.83
Özüdogru et al. (2023)	Turkey	General population	23495	1.00	1 (0.88,1.14)	1.81
Nisha et al. (2022)	India	Health service providers	350	1.14	1.14 (0.31,2.9)	0.96
Yuguero et al. (2022)	Spain	General population	972	1.44	1.44 (0.79,2.4)	1.38
Dhumal et al. (2022)	India	Health service providers	408	1.47	1.47 (0.54,3.17)	1.03
Abu-Raddad et al. (2021)	Qatar	General population	15808	1.54	1.54 (1.35,1.74)	1.80
Hammerman et al. (2022)	Israel	General population	149032	1.69	1.69 (1.63,1.76)	1.83
Qureshi et al. (2021)	USA	General population	9119	2.20	2.2 (1.91,2.53)	1.77
Rennert et al. (2022)	Unites States	General population	4544	2.24	2.24 (1.83,2.72)	1.71
Malhotra et al. (2022)	India	Health service providers	4953	2.50	2.5 (2.09,2.98)	1.72
Armstrong et al. (2021)	USA	Special risk group	6079	2.60	2.6 (2.21,3.03)	1.74
Konka et al. (2021)	Poland	General population	422	3.08	3.08 (1.65,5.21)	1.04
Cocchio et al. (2022)	Italy	General population	5552	3.24	3.24 (2.79,3.74)	1.73
Pecoraro et al. (2022)	Italy	General population	35692	3.52	3.52 (3.34,3.72)	1.82
Ando et al. (2022)	Unites States	General population	165320	3.70	3.7 (3.61,3.79)	1.83
Turbett et al. (2023)	Unites States	General population	1569	4.14	4.14 (3.21,5.25)	1.52
Bean et al. (2021)	USA	General population	1669	4.49	4.49 (3.55,5.6)	1.54
Sheehan et al. (2021)	USA	General population	1278	4.93	4.93 (3.81,6.26)	1.47
Comelli et al. (2021)	Italy	Health service providers	160	5.63	5.63 (2.6,10.41)	0.61
Al Haddad et al. (2022)	Kuwait	General population	421	7.13	7.13 (4.86,10.02)	1.04
**Overall (I^2=99.8%, p<0.001)**			**10775073**	**0.97**	**0.97 (0.81,1.14)**	

Table S6i: Incidence proportions of SARS CoV-2 reinfection generated from random effects model by background characteristics by sample size.

Study	Country	Popuilation Type	Sample Size	Incidence (%)	95% CI	Weight
**<400**	country					
Gallais et al. (2021)	France	Health service providers	345	0.29	0.29 (0.01,1.6)	0.95
Nisha et al. (2022)	India	Health service providers	350	1.14	1.14 (0.31,2.9)	0.96
Attauabi et al. (2021)	Denmark	Special risk group	315	1.27	1.27 (0.35,3.22)	0.91
Ronchini et al. (2022)	Italy	Health service providers	266	1.88	1.88 (0.61,4.33)	0.83
Masia et al. (2022)	Spain	Special risk group	150	5.33	5.33 (2.33,10.24)	0.59
Comelli et al. (2021)	Italy	Health service providers	160	5.63	5.63 (2.6,10.41)	0.61
García-Abellán et al. (2021)	Spain	General population	146	11.64	11.64 (6.93,17.99)	0.57
**Overall (I^2=87.4%, p<0.001)**			**1732**	**2.93**	**2.93 (1.01,5.71)**	
**400-999**						
Lumley et al. (2021)	UK	Health service providers	466	0.43	0.43 (0.05,1.54)	1.09
Peghin et al. (2021)	Italy	General population	546	1.10	1.1 (0.4,2.38)	1.16
Yuguero et al. (2022)	Spain	General population	972	1.44	1.44 (0.79,2.4)	1.38
Dhumal et al. (2022)	India	Health service providers	408	1.47	1.47 (0.54,3.17)	1.03
Jeffery-Smith et al. (2021)	UK	Special risk group	656	1.52	1.52 (0.73,2.79)	1.23
Konka et al. (2021)	Poland	General population	422	3.08	3.08 (1.65,5.21)	1.04
Rahman et al. (2022)	Bangladesh	Health service providers	731	5.20	5.2 (3.7,7.07)	1.27
Al Haddad et al. (2022)	Kuwait	General population	421	7.13	7.13 (4.86,10.02)	1.04
**Overall (I^2=89.4%, p<0.001)**			**4622**	**2.29**	**2.29 (1.12,3.84)**	

**>=1000**						
Aslaner et al. (2022)	Turkey	General population	104281	0.07	0.07 (0.06,0.09)	1.83
Temiz et al. (2022)	Turkey	General population	14511	0.08	0.08 (0.04,0.14)	1.79
Kim et al. (2021)	USA	General population	50327	0.08	0.08 (0.06,0.11)	1.82
Arslan et al. (2021)	Turkey	General population	32607	0.08	0.08 (0.05,0.12)	1.81
Erbas et al. (2023)	Turkey	General population	8840	0.12	0.12 (0.06,0.22)	1.77
Goldberg et al. (2022)	Israel	General population	498954	0.18	0.18 (0.17,0.19)	1.83
Nordstrom et al. (2022)	Sweden	General population	1477887	0.19	0.19 (0.18,0.2)	1.83
Akpan et al. (2022)	Liberia	General population	5459	0.20	0.2 (0.1,0.36)	1.73
Gazit et al. (2022)	Israel	General population	46035	0.23	0.23 (0.19,0.28)	1.82
Pilz et al. (2021)	Austria	General population	14840	0.27	0.27 (0.19,0.37)	1.79
Freire-Neto et al. (2022)	Brazil	General population	58097	0.29	0.29 (0.25,0.33)	1.82
Arriba et al. (2023)	Spain	General population	110726	0.31	0.31 (0.28,0.34)	1.83
Salehi-Vaziri et al. (2021)	Iran	General population	1492	0.34	0.34 (0.11,0.78)	1.51
Flacco et al. (2021)	Italy	General population	7173	0.33	0.33 (0.21,0.5)	1.75
Shaheen et al. (2022)	Saudi Arabia	General population	35288	0.37	0.37 (0.31,0.44)	1.82
Mensah et al. (a) (2022)	UK	Mixed	3860054	0.37	0.37 (0.36,0.38)	1.83
Nguyen et al. (2022)	France	General population	55338	0.40	0.4 (0.35,0.46)	1.82
Nielsen et al. (2022)	Denmark	General population	502792	0.42	0.42 (0.4,0.44)	1.83
Abu-Raddad et al. (2022)	Qatar	General population	364147	0.54	0.54 (0.52,0.56)	1.83
Berec et al. (2022)	Czech	General population	1999315	0.54	0.54 (0.53,0.55)	1.83
Rosenberg et al. (2022)	Unites States	General population	12272	0.60	0.6 (0.47,0.76)	1.79

Hønge et al. (2022)	Denmark	General population	3806	0.60	0.6 (0.38,0.91)	1.69
Hansen et al. (2021)	Denmark	General population	11068	0.65	0.65 (0.51,0.82)	1.78
Mensah et al. (b) (2022)	UK	General population	688418	0.68	0.68 (0.66,0.7)	1.83
Arslan et al. (2022)	Turkey	General population	58811	0.70	0.7 (0.63,0.77)	1.82
Guedes et al. (2023)	Brazil	Health service providers	4199	0.79	0.79 (0.54,1.1)	1.70
Islamoglu et al. (2021)	Turkey	General population	1665	0.78	0.78 (0.42,1.33)	1.54
Al-Otaiby et al. (2022)	Saudi Arabia	General population	556750	0.80	0.8 (0.78,0.82)	1.83
Michlmayr et al. (2022)	Denmark	General population	245084	0.81	0.81 (0.77,0.84)	1.83
Tavakoli et al. (2023)	Iran	General population	213768	0.83	0.83 (0.79,0.87)	1.83
Özüdogru et al. (2023)	Turkey	General population	23495	1.00	1 (0.88,1.14)	1.81
Abu-Raddad et al. (2021)	Qatar	General population	15808	1.54	1.54 (1.35,1.74)	1.80
Hammerman et al. (2022)	Israel	General population	149032	1.69	1.69 (1.63,1.76)	1.83
Sheikh et al. (2022)	UK	General population	150351	1.80	1.8 (1.73,1.87)	1.83
Hall et al. (2021)	UK	Health service providers	8278	1.87	1.87 (1.59,2.19)	1.76
Qureshi et al. (2021)	USA	General population	9119	2.20	2.2 (1.91,2.53)	1.77
Rennert et al. (2022)	Unites States	General population	4544	2.24	2.24 (1.83,2.72)	1.71
Silva et al. (2023)	Brazil	General population	1371	2.33	2.33 (1.6,3.28)	1.49
Malhotra et al. (2022)	India	Health service providers	4953	2.50	2.5 (2.09,2.98)	1.72
Armstrong et al. (2021)	USA	Special risk group	6079	2.60	2.6 (2.21,3.03)	1.74
Cohen et al. (2023)	Israel	General population	1004	2.69	2.69 (1.78,3.89)	1.39
Cocchio et al. (2022)	Italy	General population	5552	3.24	3.24 (2.79,3.74)	1.73
Pecoraro et al. (2022)	Italy	General population	35692	3.52	3.52 (3.34,3.72)	1.82

Ando et al. (2022)	Unites States	General population	165320	3.70	3.7 (3.61,3.79)	1.83
Turbett et al. (2023)	Unites States	General population	1569	4.14	4.14 (3.21,5.25)	1.52
Bean et al. (2021)	USA	General population	1669	4.49	4.49 (3.55,5.6)	1.54
Reddy et al. (2023)	India	Health service providers	1150	4.87	4.87 (3.7,6.28)	1.43
Sheehan et al. (2021)	USA	General population	1278	4.93	4.93 (3.81,6.26)	1.47
Rivelli et al. (2022)	USA	Health service providers	2625	5.94	5.94 (5.07,6.92)	1.63
**Overall (I^2=99.8%, p<0.001)**			**11632893**	**1.02**	**1.02 (0.87,1.19)**	

Table S7: Incidence rates of SARS CoV-2 reinfection per 10,000 days of observation after the initial infection generated from random effects model.

Study	Country	Population Type	Sample Size	Incidence Rate	95% CI	% Weight
Al Haddad et al. (2022)	Kuwait	General population	30	0.102	0.1 (0.07,0.15)	11.76
Gallais et al. (2021)	France	Health service providers	1	0.11	0.11 (0.02,0.78)	8.95
Abu-Raddad et al. (2021)	Qatar	General population	243	0.16	0.16 (0.14,0.18)	11.79
Nordstrom et al. (2022)	Sweden	General population	2808	0.17	0.17 (0.16,0.18)	11.80
Rosenberg et al. (2022)	Unites States	General population	74	0.4	0.4 (0.32,0.5)	11.59
Hall et al. (2021)	UK	Health service providers	155	0.76	0.76 (0.65,0.89)	11.44
Rahman et al. (2022)	Bangladesh	Health service providers	38	1.56	1.56 (1.14,2.14)	7.59
Malhotra et al. (2022)	India	Health service providers	124	1.99	1.99 (1.67,2.37)	9.29
Rivelli et al. (2022)	USA	Health service providers	156	3.55	3.55 (3.03,4.15)	7.02
Hammerman et al. (2022)	Israel	General population	2519	10.21	10.21 (9.82,10.62)	8.76
**Overall (I^2=99.9%, p<0.001, Tau^2=0.119)**				**1.64**	**1.64 (1.41,1.87)**	

Table S8a: Pooled proportion of ICU admission (with 95% CI) among SARS-CoV-2 reinfected cases generated from random effects model with zero events.

Study	Country	Population Type	Sample Size	Incidence (%)	95% CI	Weight
Sheehan et al. (2021)	USA	General population	62	0.00	0 (0,5.78)	6.35
Bean et al. (2021)	USA	General population	75	0.00	0 (0,4.8)	6.91
Temiz et al. (2022)	Turkey	General population	11	0.00	0 (0,28.49)	2.07
Mensah et al. (a) (2022)	UK	Mixed	13960	0.01	0.01 (0,0.04)	11.88
Mensah et al. (b) (2022)	UK	General population	2343	0.17	0.17 (0.05,0.44)	11.66
Al-Otaiby et al. (2022)	Saudi Arabia	General population	4454	0.76	0.76 (0.53,1.07)	11.79
Rahman et al. (2022)	Bangladesh	Health service providers	38	2.63	2.63 (0.07,13.81)	4.92
Arslan et al. (2022)	Turkey	General population	408	2.70	2.7 (1.35,4.77)	10.52
Nguyen et al. (2022)	France	General population	209	2.87	2.87 (1.06,6.14)	9.46
Aslaner et al. (2022)	Turkey	General population	78	5.13	5.13 (1.41,12.61)	7.02
Turbett et al. (2023)	Unites States	General population	65	6.15	6.15 (1.7,15.01)	6.49
Shaheen et al. (2022)	Saudi Arabia	General population	132	11.36	11.36 (6.5,18.05)	8.44
Kim et al. (2021)	USA	General population	14	21.43	21.43 (4.66,50.8)	2.49
**Overall (I^2=94.7%, p<0.001, Tau^2=0.02)**				**1.31**	**1.31 (0.29,2.83)**	

Table S8b: Pooled proportion of ICU admission (with 95% CI) among SARS-CoV-2 reinfected cases generated from random effects model without zero events.

Study	Country	Population Type	Sample Size	Incidence (%)	95% CI	Weight
Mensah et al. (a) (2022)	UK	Mixed	13960	0.01	0.01 (0,0.04)	13.96
Mensah et al. (b) (2022)	UK	General population	2343	0.17	0.17 (0.05,0.44)	13.70
Al-Otaiby et al. (2022)	Saudi Arabia	General population	4454	0.76	0.76 (0.53,1.07)	13.85
Rahman et al. (2022)	Bangladesh	Health service providers	38	2.63	2.63 (0.07,13.81)	5.88
Arslan et al. (2022)	Turkey	General population	408	2.70	2.7 (1.35,4.77)	12.39
Nguyen et al. (2022)	France	General population	209	2.87	2.87 (1.06,6.14)	11.17
Aslaner et al. (2022)	Turkey	General population	78	5.13	5.13 (1.41,12.61)	8.34
Turbett et al. (2023)	Unites States	General population	65	6.15	6.15 (1.7,15.01)	7.72
Shaheen et al. (2022)	Saudi Arabia	General population	132	11.36	11.36 (6.5,18.05)	9.99
Kim et al. (2021)	USA	General population	14	21.43	21.43 (4.66,50.8)	3.00
**Overall (I^2=96%, p<0.001, Tau^2=0.019)**				**1.98**	**1.98 (0.65,3.82)**	

Table S9a: Pooled proportion of mortality (with 95% CI) among SARS-CoV-2 reinfected cases generated from random effects model with zero events.

Study	Country	Population Type	Sample Size	Incidence (%)	95% CI	Weight
Temiz et al. (2022)	Turkey	General population	11	0.00	0 (0,28.49)	1.69
Arriba et al. (2023)	Spain	General population	340	0.00	0 (0,1.08)	6.08
Abu-Raddad et al. (2021)	Qatar	General population	54	0.00	0 (0,6.6)	4.12
Guedes et al. (2023)	Brazil	Health service providers	33	0.00	0 (0,10.58)	3.32
Gazit et al. (2022)	Israel	General population	108	0.00	0 (0,3.36)	5.09
Mensah et al. (b) (2022)	UK	General population	2343	0.00	0 (0,0.16)	6.59
Al-Otaiby et al. (2022)	Saudi Arabia	General population	4454	0.09	0.09 (0.02,0.23)	6.63
Goldberg et al. (2022)	Israel	General population	894	0.11	0.11 (0,0.62)	6.44
Nielsen et al. (2022)	Denmark	General population	2115	0.28	0.28 (0.1,0.62)	6.58
Nguyen et al. (2022)	France	General population	209	0.96	0.96 (0.12,3.41)	5.75
Arslan et al. (2022)	Turkey	General population	408	1.96	1.96 (0.85,3.83)	6.17
Pilz et al. (2021)	Austria	General population	40	2.50	2.5 (0.06,13.16)	3.64
Mensah et al. (a) (2022)	UK	Mixed	13960	2.77	2.77 (2.51,3.06)	6.67
Qureshi et al. (2021)	USA	General population	63	3.17	3.17 (0.39,11)	4.36
Arslan et al. (2021)	Turkey	General population	27	3.70	3.7 (0.09,18.97)	3.00
Aslaner et al. (2022)	Turkey	General population	78	3.85	3.85 (0.8,10.83)	4.67
Flacco et al. (2021)	Italy	General population	24	4.17	4.17 (0.11,21.12)	2.81
Turbett et al. (2023)	Unites States	General population	65	4.62	4.62 (0.96,12.9)	4.41
Kim et al. (2021)	USA	General population	14	7.14	7.14 (0.18,33.87)	2.00
Erbas et al. (2023)	Turkey	General population	11	9.09	9.09 (0.23,41.28)	1.69
Armstrong et al. (2021)	USA	Special risk group	156	12.82	12.82 (8.01,19.1)	5.49
Yuguero et al. (2022)	Spain	General population	14	14.29	14.29 (1.78,42.81)	2.00
Attauabi et al. (2021)	Denmark	Special risk group	4	25.00	25 (0.63,80.59)	0.78
**Overall (I^2=95.7%, p<0.001, Tau^2=0.03)**				**0.71**	**0.71 (0.02,2.01)**	

Table S9b: Pooled proportion of mortality (with 95% CI) among SARS-CoV-2 reinfected cases generated from random effects model without zero events.

Study	Country	Population Type	Sample Size	Incidence (%)	95% CI	Weight
Al-Otaiby et al. (2022)	Saudi Arabia	General population	4454	0.09	0.09 (0.02,0.23)	9.13
Goldberg et al. (2022)	Israel	General population	894	0.11	0.11 (0,0.62)	8.86
Nielsen et al. (2022)	Denmark	General population	2115	0.28	0.28 (0.1,0.62)	9.05
Nguyen et al. (2022)	France	General population	209	0.96	0.96 (0.12,3.41)	7.89
Arslan et al. (2022)	Turkey	General population	408	1.96	1.96 (0.85,3.83)	8.48
Pilz et al. (2021)	Austria	General population	40	2.50	2.5 (0.06,13.16)	4.95
Mensah et al. (a) (2022)	UK	Mixed	13960	2.77	2.77 (2.51,3.06)	9.18
Qureshi et al. (2021)	USA	General population	63	3.17	3.17 (0.39,11)	5.94
Arslan et al. (2021)	Turkey	General population	27	3.70	3.7 (0.09,18.97)	4.06
Aslaner et al. (2022)	Turkey	General population	78	3.85	3.85 (0.8,10.83)	6.37
Flacco et al. (2021)	Italy	General population	24	4.17	4.17 (0.11,21.12)	3.80
Turbett et al. (2023)	Unites States	General population	65	4.62	4.62 (0.96,12.9)	6.01
Kim et al. (2021)	USA	General population	14	7.14	7.14 (0.18,33.87)	2.71
Erbas et al. (2023)	Turkey	General population	11	9.09	9.09 (0.23,41.28)	2.29
Armstrong et al. (2021)	USA	Special risk group	156	12.82	12.82 (8.01,19.1)	7.53
Yuguero et al. (2022)	Spain	General population	14	14.29	14.29 (1.78,42.81)	2.71
Attauabi et al. (2021)	Denmark	Special risk group	4	25.00	25 (0.63,80.59)	1.05
**Overall (I^2=95.8%, p<0.001, Tau^2=0.03)**				**1.58**	**1.58 (0.29,3.53)**	

Table S10: Proportions of reinfected cases according to their severity level, and supplemental oxygenation and mechanical ventilation requirements.

Study	Study Design	Country	Study Mid-year	Mild (%)	Moderate (%)	Severe (%)	Moderate -severe (%)	Supple-mental Oxygen (%)	Mechanical ventilation requirement (1%)	Sample size (Reinfected cases)
**General population**										
Abu-Raddad et al. (2021)	Retrospective	Qatar	2020	100.00	-	-	-	-	-	243
Garcia-Abellan et al. (2021)	Prospective	Spain	2020	-	-	-	9.60	-	-	17
Peghin et al. (2021)	Prospective	Italy	2020	83.30	-	-	-	-	-	6
Salehi-Vaziri et al. (2021)	Prospective	Iran	2020	-	100.00	-	-	-	-	5
Shaheen et al. (2022)	Retrospective	Saudi Arabia	2020	68.90	-	-	4.03	-	-	132
Al Haddad et al. (2022)	Retrospective	Kuwait	2020	31.00	-	13.80	-	-	-	29
Al-Otaiby et al. (2022)	Retrospective	Saudi Arabia	2021	-	-	0.70	-	-	-	4454
Aslaner et al. (2022)	Retrospective	Turkey	2020	89.74	6.41	3.85	-	-	-	78
Erbas et al. (2023)	Retrospective	Turkey	2020	54.55	9.09	18.18	-	-	-	11
Goldberg et al. (2022)	Retrospective	Israel	2020	-	-	1.79	-	-	-	894
Bean et al. (2021)	Retrospective	USA	2020	-	-	-	-	-	0.00	75
Kim et al. (2021)	Retrospective	USA	2021	-	-	-	-	-	14.30	14
Qureshi et al. (2021)	Retrospective	USA	2020	-	-	-	-	-	0.00	63
Sheehan et al. (2021)	Retrospective	USA	2020	-	-	-	-	-	0.00	62
**Health Service Providers**										
Rahman et al. (2022)	Prospective	Bangladesh	2020	-	-	2.63	-	2.63	-	38
Guedes et al. (2023)	Prospective	Brazil	2021	100.00	-	-	-	-	-	33
Reddy et al. (2023)	Prospective	India	2021	94.64	3.57	1.79	-	-	-	56
**Special risk group**										
Jeffery-Smith et al. (2021)	Prospective	UK	2020	25.00	-	-	-	-	-	4
Attauabi et al. (2021)	Prospective	Denmark	2020	75.00	-	25.00	-	-	-	4

Figure S2: Sensitivity analysis of hospital admission and mortality proportions (with 95% CI) among SARS-CoV-2 reinfected cases generated from random effects.

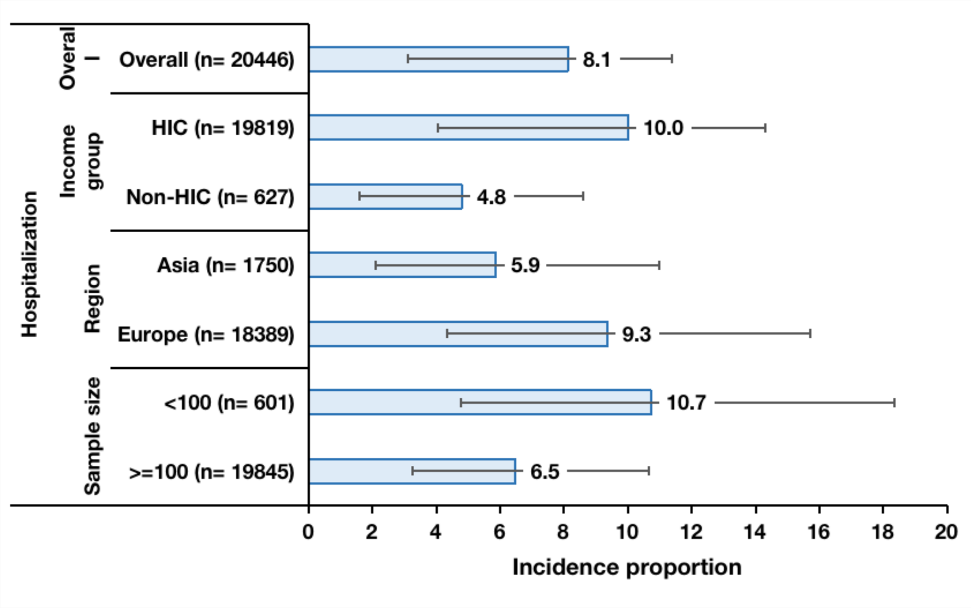

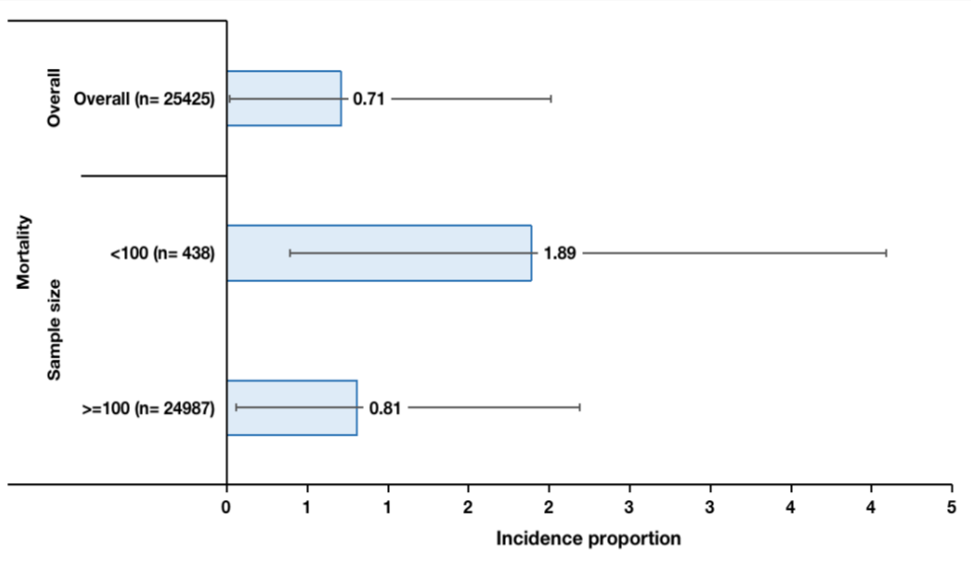

Table S11: Sensitivity analysis of SARS-CoV-2 reinfection incidence proportions (with 95% CI) of all prospective studies based on population type generated from random effects.

Study	Country	Sample Size	Incidence (%)	95% CI	Weight
**General Population**					
Salehi-Vaziri et al. (2021)	Iran	1492	0.34	0.34 (0.11,0.78)	5.85
Mensah et al. (b) (2022)	UK	688418	0.68	0.68 (0.66,0.7)	6.45
Islamoglu et al. (2021)	Turkey	1665	0.78	0.78 (0.42,1.33)	5.90
Peghin et al. (2021)	Italy	546	1.10	1.1 (0.4,2.38)	5.03
Sheikh et al. (2022)	UK	150351	1.80	1.8 (1.73,1.87)	6.44
Silva et al. (2023)	Brazil	1371	2.33	2.33 (1.6,3.28)	5.80
Cohen et al. (2023)	Israel	1004	2.69	2.69 (1.78,3.89)	5.59
Garcia-Abellan et al. (2021)	Spain	146	11.64	11.64 (6.93,17.99)	3.14
**Overall (I^2=99.5%, p<0.001)**			1.59	1.59 (0.95,2.39)	
**Health Service Provider**					
Gallais et al. (2021)	France	345	0.29	0.29 (0.01,1.6)	4.46
Lumley et al. (2021)	UK	466	0.43	0.43 (0.05,1.54)	4.85
Guedes et al. (2023)	Brazil	4199	0.79	0.79 (0.54,1.1)	6.22
Hall et al. (2021)	UK	8278	1.87	1.87 (1.59,2.19)	6.33
Ronchini et al. (2022)	Italy	266	1.88	1.88 (0.61,4.33)	4.09
Reddy et al. (2023)	India	1150	4.87	4.87 (3.7,6.28)	5.69
Rahman et al. (2022)	Bangladesh	731	5.20	5.2 (3.7,7.07)	5.33
Rivelli et al. (2022)	USA	2625	5.94	5.94 (5.07,6.92)	6.09
**Overall (I^2=97%, p<0.001)**			2.25	2.25 (1.03,3.89)	
**Special Risk Group**					
Attauabi et al. (2021)	Denmark	315	1.27	1.27 (0.35,3.22)	4.33
Jeffery-Smith et al. (2021)	UK	656	1.52	1.52 (0.73,2.79)	5.22
Masia et al. (2022)	Spain	150	5.33	5.33 (2.33,10.24)	3.19
**Overall (I^2=. %, p= .)**			2.16	2.16 (0.69,4.32)	

Figure S3: Leave-one-out analysis for incidence proportions of SARS CoV-2 reinfection for 64 studies included in the meta-analysis.

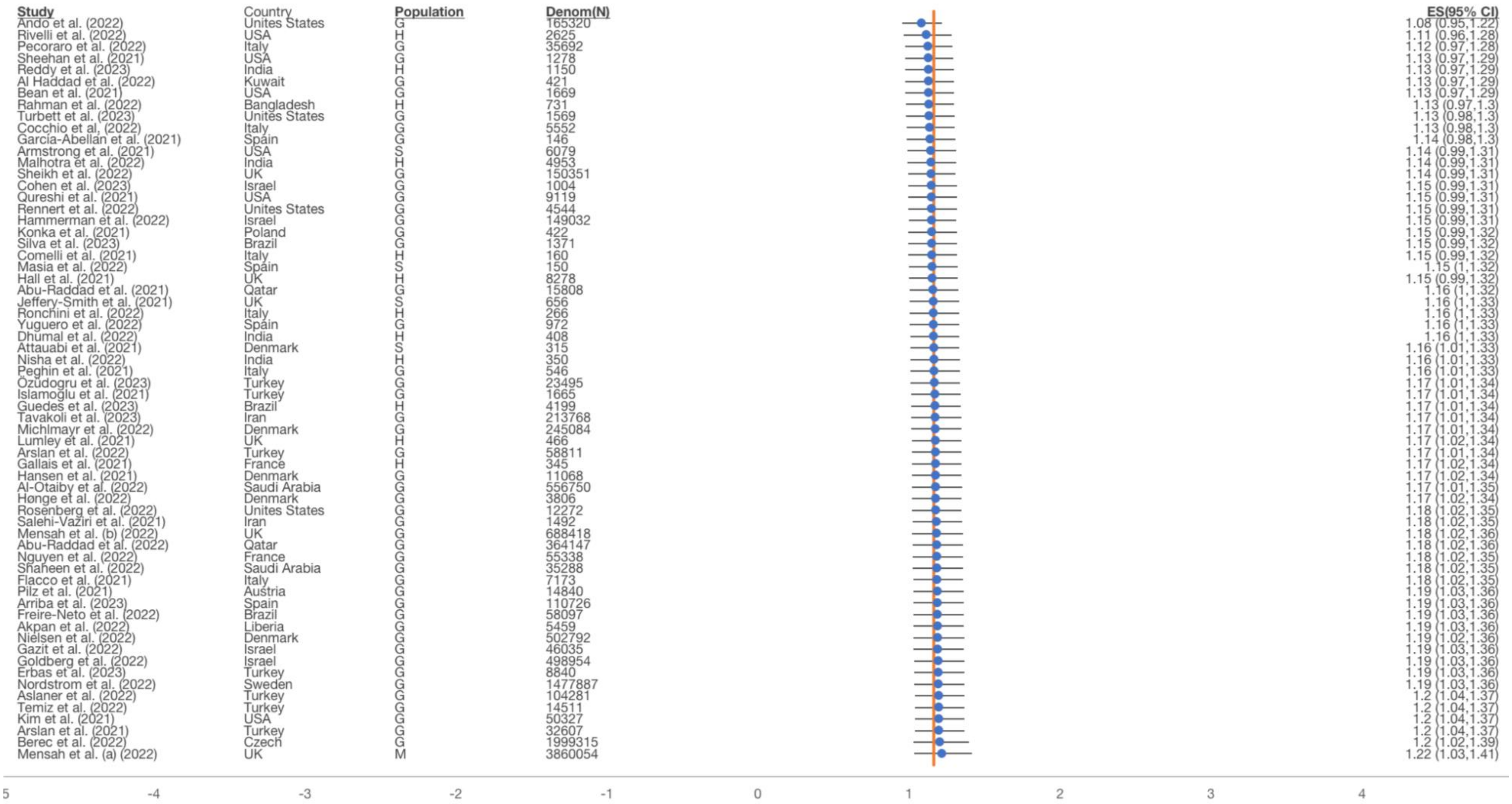

G: General Population, H: Health Service Provider, M: Mixed Population, S: Special Risk Group (Long Term Care Facilities residents and persons with autoimmune diseases).

Table S12a: Checklist for Quality assessment and their results for studies assessed using JBI checklist for Prevalence studies.

No.	Study/ Criteria ☐Yes ☐No ☐Unclear ☐Not applicable	1. Was the sample frame appropriate to address the target population?	2. Were study participants sampled in an appropriate way?	3. Was the sample size adequate?	4. Were the study subjects and the setting described in detail?	5. Was the data analysis conducted with sufficient coverage of the identified sample?	6. Were valid methods used for the identification of the condition?	7. Was the condition measured in a standard, reliable way for all participants?	8. Was there appropriate statistical analysis?	9. Was the response rate adequate, and if not, was the low response rate managed appropriately?
1	Garcia-Abellan et al.	Yes	Yes	Yes	Yes	Yes	Yes	Yes	Yes	N/A
2	Akpan et al.	Yes	Yes	Yes	Yes	Yes	Yes	Yes	Yes	N/A
3	Mensah et al.	Yes	Yes	Yes	Yes	Yes	Yes	Yes	Yes	N/A
4	Kim et al.	Yes	Yes	Yes	Yes	Yes	Yes	Yes	Yes	N/A
5	Nisha et al.	Yes	Yes	Yes	Yes	Yes	Yes	Yes	Yes	N/A
6	Arslan et al.	Yes	Yes	Yes	Yes	Yes	Yes	Yes	Yes	N/A
7	Hønge et al.	Yes	Yes	Yes	Yes	Yes	Yes	Yes	Yes	N/A
8	Comelli et al.	Yes	Yes	Yes	Yes	Yes	Yes	Yes	Yes	N/A
9	Flacco et al.	Yes	Yes	Yes	Yes	Yes	Yes	Yes	Yes	N/A
10	Sheehan et al.	Yes	Yes	Yes	Yes	Yes	Yes	Yes	Yes	N/A
11	Jeffery-Smith et al.	Yes	Yes	Yes	Yes	Yes	Yes	Yes	Yes	N/A
12	Qureshi et al.	Yes	Yes	Yes	Yes	Yes	Yes	Yes	Yes	N/A
13	Abu-Raddad et al.	Yes	Yes	Yes	Yes	Yes	Yes	Yes	Yes	N/A
14	Armstrong et al.	Yes	Yes	Yes	Yes	Yes	Yes	Yes	Yes	N/A
15	Mensah et al.	Yes	Yes	Yes	Yes	Yes	Yes	Yes	Yes	N/A
16	Konka et al.	Yes	Yes	Yes	Yes	Yes	Yes	Yes	Yes	N/A
17	Salehi-Vaziri et al.	Yes	Yes	Yes	Yes	Yes	Yes	Yes	Yes	N/A
18	Pilz et al.	Yes	Yes	Yes	Yes	Yes	Yes	Yes	Yes	N/A
19	Dhumal et al.	Yes	Yes	Yes	Yes	Yes	Yes	Yes	Yes	N/A
20	Nguyen et al.	Yes	Yes	Yes	Yes	Yes	Yes	Yes	Yes	N/A
21	Rivelli et al.	Yes	Yes	Yes	Yes	Yes	Yes	Yes	Yes	N/A

22	Arslan et al.	Yes	Yes	Yes	Yes	Yes	Yes	Yes	Yes	N/A
23	Shaheen et al.	Yes	Yes	Yes	Yes	Yes	Yes	Yes	Yes	N/A
24	Turbett et al.	Yes	Yes	Yes	No	No	Yes	Yes	Yes	N/A
25	Temiz et al.	Yes	Yes	Yes	No	No	Yes	Yes	Yes	N/A

Table S12b: Checklist for Quality assessment and their results for studies assesed using JBI checklist for Cohort studies.

No.	Study/Criteria ☐Yes ☐ No ☐ Unclear ☐ Not applicable	1. Were the two groups similar and recruited from the same population?	2. Were the exposures measured similarly to assign people to both exposed and unexposed groups?	3. Was the exposure measured in a valid and reliable way?	4. Were confounding factors identified?	5. Were strategies to deal with confounding factors stated?	6. Were the groups/participants free of the outcome at the start of the study (or at the moment of exposure)?	7. Were the outcomes measured in a valid and reliable way?	8. Was the follow up time reported and sufficient to be long enough for outcomes to occur?	9. Was follow up complete, and if not, were the reasons to loss to follow up described and explored?	10. Were strategies to address incomplete follow up utilized?	11. Was appropriate statistical analysis used?
1	Hansen et al.	Yes	Yes	Yes	Yes	Yes	Yes	Yes	Yes	Yes	Yes	Yes
2	Hammerman et al.	Yes	Yes	Yes	Yes	Yes	Yes	Yes	Yes	Yes	Yes	Yes
3	Bean et al.	Yes	Yes	Yes	Yes	Yes	No	Yes	No	Yes	No	Yes
4	Nordstrom et al.	Yes	Yes	Yes	Yes	Yes	Yes	Yes	Yes	Yes	Yes	Yes
5	Rahman et al.	Yes	Yes	Yes	No	No	Yes	Yes	Yes	Yes	Yes	Yes
6	Sheikh et al.	Yes	No	Yes	Yes	Yes	No	Yes	Yes	Yes	Yes	Yes
7	Lumley et al.	Yes	Yes	Yes	Yes	Yes	Yes	Yes	Yes	Yes	Yes	Yes
8	Gallais et al.	Yes	Yes	Yes	Yes	Yes	Yes	Yes	Yes	Yes	Yes	Yes
9	Peghin et al.	Yes	Yes	Yes	Yes	Yes	Yes	No	Yes	No	Yes	Yes

10	Ronchini et al.	Yes	Yes	Yes	Yes	Yes	Yes	Yes	Yes	Yes	Yes	Yes
11	Islamoglu et al.	Yes	Yes	Yes	Yes	Yes	Yes	Yes	Yes	Yes	Yes	Yes
12	Hall et al.	Yes	Yes	Yes	Yes	Yes	Yes	Yes	Yes	No	Yes	Yes
13	Malhotra et al.	Yes	Yes	Yes	Yes	Yes	Yes	No	Yes	Yes	Yes	Yes
14	Abu-Raddad et al.	Yes	Yes	Yes	Yes	Yes	Yes	Yes	Yes	Yes	Yes	Yes
15	Al-Otaiby et al.	Yes	Yes	Yes	Yes	Yes	Yes	Yes	Yes	Yes	Yes	Yes
16	Al Haddad et al.	Yes	Yes	Yes	Yes	Yes	Yes	Yes	Yes	Yes	Yes	Yes
17	Ando et al.	Yes	Yes	Yes	Yes	Yes	Yes	Yes	Yes	Yes	Yes	Yes
18	Aslaner et al.	Yes	Yes	Yes	Yes	Yes	Yes	Yes	Yes	Yes	Yes	Yes
19	Attauabi et al.	Yes	Yes	Yes	Yes	Yes	Yes	Yes	Yes	Yes	Yes	Yes
20	Berec et al.	Yes	Yes	Yes	Yes	Yes	Yes	Yes	Yes	Yes	Yes	Yes
21	Cocchio et al.	Yes	Yes	Yes	No	No	Yes	Yes	Yes	Yes	Yes	Yes
22	Cohen et al.	Yes	Yes	Yes	No	No	Yes	Yes	Yes	Yes	Yes	Yes
23	Arriba et al.	Yes	No	Yes	Yes	Yes	Yes	Yes	Yes	Yes	Yes	Yes
24	Erbas et al.	Yes	Yes	Yes	No	No	Yes	Yes	Yes	Yes	Yes	Yes
25	Flacco et al.	Yes	Yes	Yes	Yes	No	Yes	Yes	Yes	Yes	Yes	Yes

26	Freire-Neto et al.	Yes	Yes	Yes	No	No	Yes	Yes	Yes	Yes	Yes	Yes
27	Gazit et al.	Yes	Yes	Yes	Yes	Yes	Yes	Yes	Yes	Yes	Yes	Yes
28	Goldberg et al.	Yes	Yes	Yes	No	No	Yes	Yes	Yes	Yes	Yes	Yes
29	Guedes et al.	Yes	Yes	Yes	Yes	No	Yes	Yes	Yes	No	No	Yes
30	Michlmayr et al.	Yes	Yes	Yes	Yes	Yes	Yes	Yes	Yes	Yes	Yes	Yes
31	Silva et al.	Yes	Yes	Yes	Yes	Yes	Yes	Yes	Yes	No	Yes	Yes
32	Tavakoli et al.	Yes	Yes	Yes	Yes	Yes	Yes	Yes	Yes	No	No	Yes
33	Pecoraro et al.	Yes	Yes	Yes	No	No	Yes	Yes	Yes	Yes	Yes	Yes
34	Masia et al.	Yes	Yes	Yes	Yes	Yes	Yes	Yes	Yes	Yes	Yes	Yes
35	Rosenberg et al.	Yes	No	Yes	Yes	No	Yes	Yes	Yes	No	No	Yes
36	Rennert et al.	Yes	No	Yes	Yes	Yes	Yes	Yes	Yes	No	No	Yes
37	Nielsen et al.	Yes	Yes	Yes	Yes	Yes	Yes	Yes	Yes	Yes	Yes	Yes
38	Yuguero et al.	Yes	Yes	Yes	Yes	No	Yes	Yes	No	No	No	Yes
39	Özüdogru et al.	Yes	No	Yes	No	No	Yes	No	No	No	No	Yes
40	Reddy et al.	Yes	Unclear	Yes	No	No	Yes	Yes	Yes	Unclear	No	Yes
